# Supplementary material for: The invisible plant technology of Prehistoric Southeast Asia: Indirect evidence for basket and rope making at Tabon Cave, Philippines, 39–33,000 years ago
Source: PLoS One. 2023 Jun 30;18(6):e0281415. doi: 10.1371/journal.pone.0281415 (PMC10313078; doi:10.1371/journal.pone.0281415)
Supplement: S1 File — (PDF) [file pone.0281415.s001.pdf]

Supporting information

to:

Xhauflair et al, The invisible plant technology of Prehistoric Southeast Asia: Indirect evidence for basket and rope making at Tabon Cave, Philippines, 39-33,000 years ago.

Content:

1- The concept of *chaîne opératoire* ……………………………………..p.2
2- Experimental variables recorded……………………………………..p.3
3- Use-wear and retouch attributes recorded……………………………p.8
4- Details of the experiments: variables and use-wear…………..p.14
5- Lithic artefacts from Tabon Cave studied in the frame of this research:…………..p.59-60

The concept of *chaîne opératoire* that we refer to in this study

We used the concept of *chaîne opératoire* as developed in Anthropology of Technology to describe technological activities involving wild plants that we witnessed in the field during the ethnoarchaeology part of this investigation.
This concept was developed in the field of Anthropology of Technology and later borrowed by archaeologists (Balfet, 1991; Cresswell, 2010; Karlin et al., 1991; Lemonnier, 1992)(Cresswell, 2010- 1st ed. 1976; Balfet, 1991; Karlin et al., 1991; Lemonnier, 1992). Although it is implicit in the work of Mauss ((Mauss, 2002 - 1st ed. 1947) and explicitly mentioned by Leroi-Gourhan (Leroi-Gourhan, 1964 t.1: 164), its first definition was given by the ethnographer Cresswell (2010-1st ed. 1976: 25, our translation from French): "A *chaîne opératoire* is a series of operations that transform a raw material into a product, the latter being either a consumer good or a tool". This notion is a powerful theoretical tool of analysis that allows decomposing a technical act into a sequence of operations, the latter being the basic units of action on the matter. The technical atom, according to Balfet (Balfet, 1975) is the gesture. Using this concept, we transcribed the technical activities that we witnessed in ethnographic context into diagrams based on the articulation offered by Sigaut (Sigaut, 1987): Material state n -----> Operation -----> Material state n+1.

References

Balfet, H., 1975. Technologie. Eléments d'ethnologie 2, 44–79.
Balfet, H., Centre national de la recherche scientifique, 1991. Observer l'action technique : des chaînes opératoires, pour quoi faire? Éd. du Centre national de la recherche scientifique, Paris.
Cresswell, R., 2010. Techniques et culture : les bases d'un programme de travail. Techniques & Culture. Revue semestrielle d'anthropologie des techniques 20–45.
Karlin, C., BODU, P., Pelegrin, J., 1991. Processus techniques et chaînes opératoires: Comment les préhistoriens s' approprient un concept élaboré par les ethnologues. Observer l'action technique: des chaînes opératoires, pour quoi faire 101–117.
Lemonnier, P., 1992. Elements for an anthropology of technology, Anthropological papers - University of Michigan. Museum of Anthropology, ISSN 0076-8367. Museum of anthropology University of Michigan, Ann Arbor, Mich., Etats-Unis.
Leroi-Gourhan, A., 1964. Le geste et la parole. Albin Michel, Paris, France.
Mauss, M., Paulme, D., 2002. Manuel d'ethnographie. Payot & Rivages, Paris, France.
Sigaut, F., 1987. Des idées pour observer. Techniques et culture.

Experimental variables recorded

The fields relative to the tool are based on the experimental recording form established and used by Pawlik (1995).

The different experimental variables recorded are the following:

GENERAL INFORMATION

-The duration of the experiment

-The date

-The weather during the experiment: 1-raining 2-dry

ABOUT THE CONTACT MATERIAL

-The taxonomic ID of the plant

-Its freshness: 1-fresh 2-dry. A plant was considered to be fresh up to 24 hours after its procurement. It was considered to be dry starting 3 months after the collection in the case of *Calamus merrillii*, *Pterospermum diversifolium* and *Dinochloa luconiae* which are thicker. Culms of *Schizostachyum* cf. *lima* were bought already dry in a little shop in Quezon circle in Manila. They were processed three weeks after the purchase.

-The part of the plant which was worked

-Its dimensions

-Its degree of hardness: 1-soft 2-semi-soft 3-semi-hard 4-hard (Odell, 1980; Odell & Odell-Vereecken, 1980). This indication is the fruit of HX subjective perception which however gives an idea of the relative level of hardness of the 15 plants processed.

-Its degree of moisture: 1-very dry 2-rather dry 3-semi moist 4-very moist. Similarly, this field does not provide an absolute degree of water content in the plant but informs about the experimenter subjective perception of the moisture.

-Its degree of penetrability: 1-Difficult to penetrate 2-Medium to penetrate 3-Highly penetrable (Rots, 2010). It gives information about the penetrability HX experienced while performing a task. Its degree varies from one taxon to another, but also within the same plant, depending on the part worked and the way of the motion to the fibers.

ABOUT THE EXPERIMENTAL TOOL

-The tool ID

-The type of flake: 1-side struck/short flake (the technological width is larger than the technological length) 2-end struck/long flake (the technological length is larger than the technological width).

-The amount of cortex, meaning here the external surface of the blocks at the time of their collection: 1- absent 2- <1/3 3- <2/3 4->2/3

-*The degree of fragmentation*: 1-non fragmented 2-proximal fragment 3-distal fragment 4-siret 5-incomplete width 6-incomplete length 7-multiple fractures. This field refers here to the fragmentation that occurred during the knapping process and is an indicator of the amount of internal cracks present in raw the material.

-*Measurements*. These include the technological length and width (in the case of flakes), maximum length and width and the maximum thickness (for all stone tools).

The *technological length* of a flake is taken from the striking platform to the distal edge, following the debitage axis. The *technological width* is taken perpendicularly to the debitage axis.

To measure the *maximum length and width*, the tool was oriented to fit within the smallest possible rectangle. The longest width was placed down, constituting the base. The axis perpendicular to it, from the base to the tip of the tool was measured as the maximum length. The maximum width was measured following the axis perpendicular to the maximum length. (See Figure a)

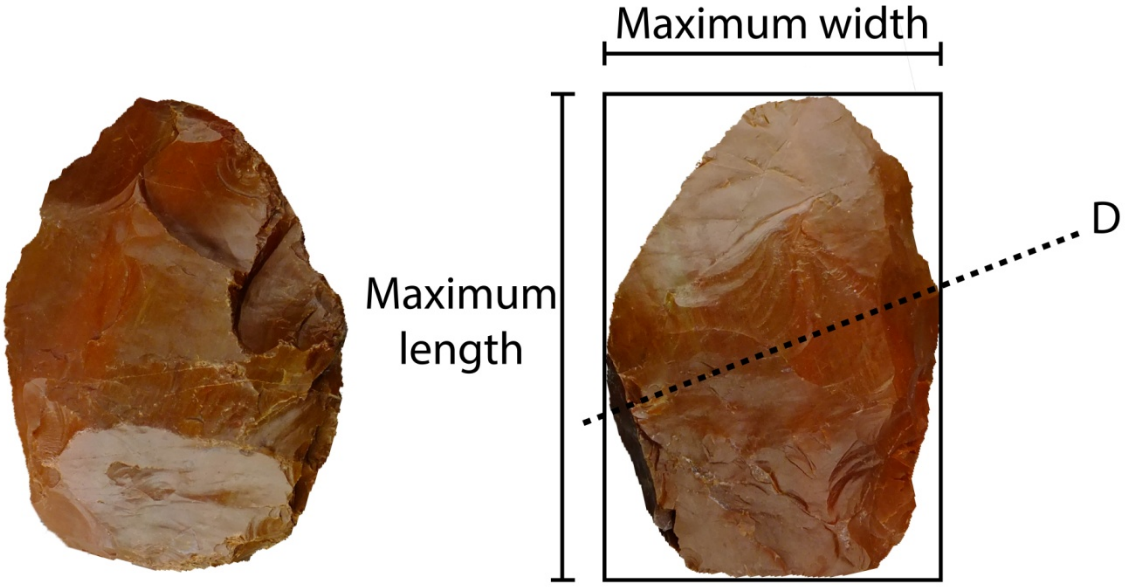

Figure a: Measure of the maximum length and width of a flake. The axis D corresponds to the debitage axis.

The *maximum thickness* is the maximum depth of the tool. In the case of flakes, it was measured perpendicular to the plane of percussion and for other tools perpendicular to the plane of the lower face (Keeley, 1980).

-*The location of the used area* (It was also reported on a picture of the experimental tool in the field)

-*The length of the used area*

-*The edge angle of the used area.* The minimum and the maximum angle measured with a goniometer.

-*The edge profi*le: 1- biplane 2- biconvex 3-biconcave 4-plano-convex 5- plano-concave 6- convexo-concave 7-convexo-plan 8-concavo-plan 9-concavo-convex (Van Gijn, 1989)

-*The edge form*: 1- straight 2-convex 3-concave 4-irregular (See Figure b)

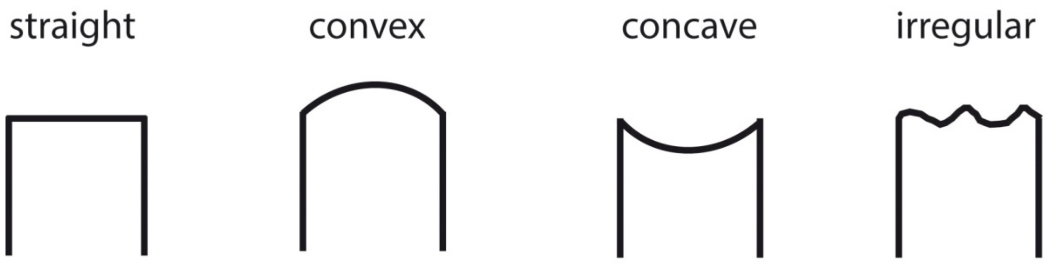

Figure b: Different types of edge form. View of a tool, dorsal/upper face upwards, seen from above.

-*The edge torsion:* 1-none 2-minimal 3-slight 4-strong (See Figure c)

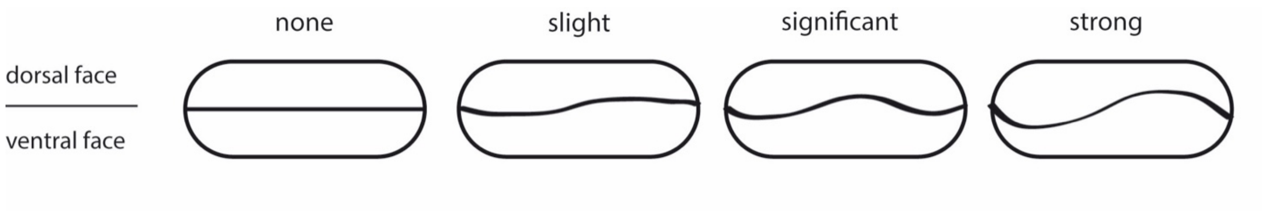

Figure c: Degree of edge torsion. Front view of the active edge.

-*Number of edge torsion(s)* (if any): 1-one 2-several (See Figure d)

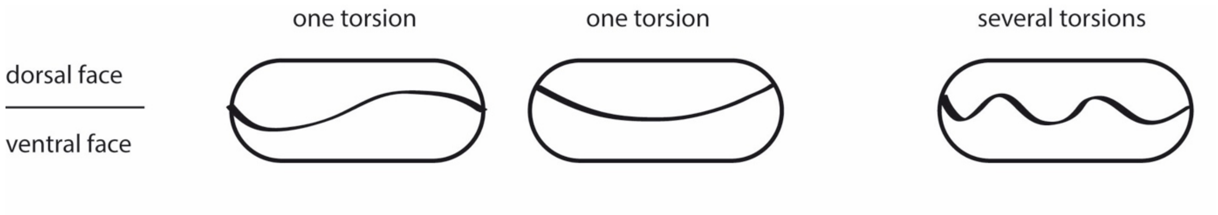

Figure d: Number of edge torsion(s). Front view of the active edge.

<u>ABOUT THE MOTION(S)</u>

-*The part of the tool which is handled* (It was also reported on a picture of the experimental tool in the field)

-*The mode of prehensility*: 1-bare hands 2-hands protected with gloves. None of the experimental tool was hafted.

-*The type of motion*: 1-adzing 2-striking 3-scraping 4-grooving 5-perforating 6-drilling 7-sawing 8-chopping 9-shaving 10-splitting 11-thinning down, or other

-The type of percussion: 1-dynamic 2-resting (translation made by Lemonnier, 1992 of the categories established by Leroi-Gourhan, 1971 – 1st ed. 1943).

-*The face of the tool in contact with the raw material:* 1-dorsal 2-ventral 3-symmetrical. Symmetrical means here that both faces were in contact with the worked material, simultaneously but also one after the other (ex: splitting).

-*The contact angle* (this field refer to the angle formed by the tool and the worked material)*:* 1-0-29° 2-30-59° 3-60-89° 4-90°

-*Direction of the motion to the active edge*: 1-longitudinal 2-transversal 3-crosswise (See Figure e)

-*The way of the motion*: 1-unidirectional 2-bidirectional (See Figure e)

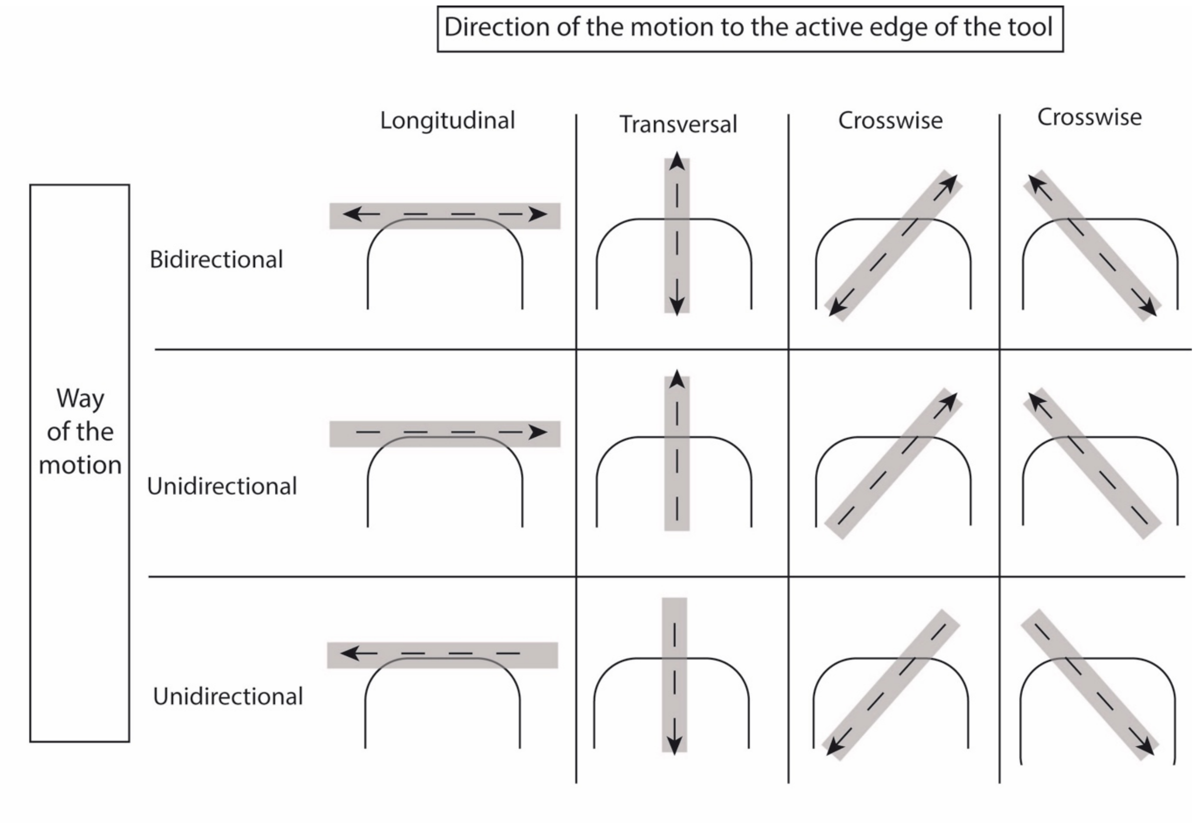

Figure e: Directions of the motion to the active edge of the tool and ways of the motion. The half rectangles represent stone tools seen from above, dorsal face upwards. The wide grey lines containing arrows represent the directions and the ways of the motion.

-*The direction of the motion to the fibers of the plant*: 1-longitudinal 2-transversal 3-crosswise (See Figure f)

-The tool efficiency to achieve the task

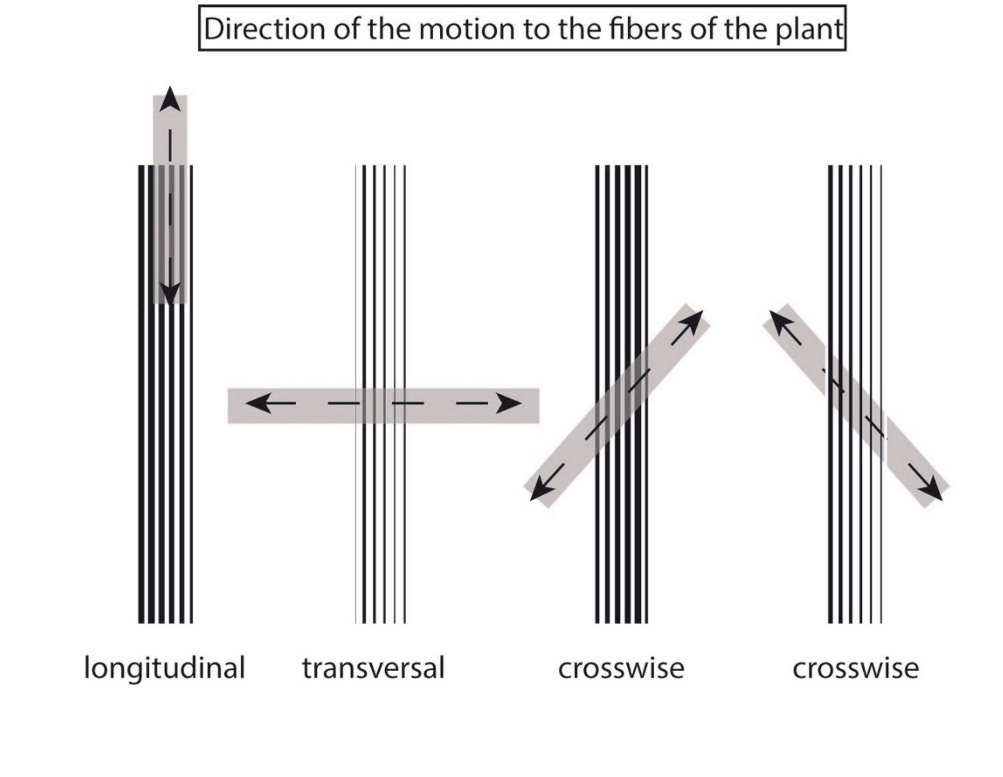

Figure f: Direction of the motion to the fibers of the plant. The sets of parallel lines represent plant fibers. The wide grey lines containing arrows represent the direction of the motion.

Use-wear and retouch attributes recorded

The different attributes recorded on stone tools are based on the recording form used by Pawlik (1995), with new additions and modifications. When the fields are based on other sources, these are indicated.

In addition, we recorded basic information about the archaeological artefacts (e.g. measurements, edge profile, edge angle) following the protocol described for the experimental tools (See the experimental variables section).

<u>NAKED EYE OBSERVATIONS</u>

-*Breakage*: 1-visible 2-non visible. Breakage refers her to very large mostly non-conchoidal fractures, rather angular and probably following the internal cracks of the rock.

-*Scarring*: 1-visible 2-non visible

-*Gloss*: 1-visible 2-non visible. Gloss refers to a shiny surface visible with naked eye. It usually corresponds to polish when the area is observed under high-power microscope.

-*Rounding*: 1-visible 2-non visible.

<u>LOW POWER OBSERVATION</u>

<u>Scars and notches</u>

-*Micro-scars visibility*: 1-present 2-absent

-*Amount of micro-scars*: 1-none 2-a few 3-a medium amount 4-many

-*Distribution on the two face of the tool*: 1-predominant dorsal 2-predominant ventral 3-alternating 4-both faces 5-undetermined (See Figure a)

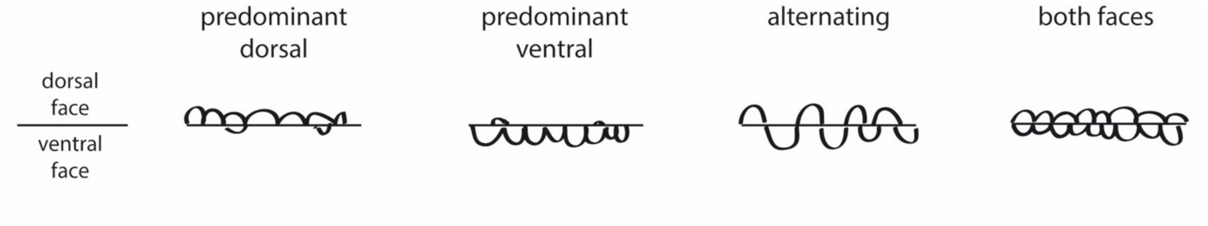

Figure g: Distribution of the scars on the two faces of a tool.

-*Location:* 1-all along the edge 2-localised

-*Distribution*: 1-continuous 2-wide 3-one patch 4-distinct patches 3-distinct patches, wide in between 4-overlapping (Classification modified after Rots, 2010 and Pawlik, 1995)

-*Second edge row* a second generation of micro-scars): 1-none 2-interrupted 3-developped

-*Scar direction to the edge 1 and 2* (the most and the second most common directions): 1-perpendicular 2-transverse (oblique) unidirectional 3-transverse (oblique) bidirectional 4-

perpendicular and transverse (oblique) unidirectional 5-perpendicular and transverse (oblique) bidirectional

-*Macro-striations associated with the scars/notches*: 1-present 2-absent

-*Crushing*: 1-present 2-absent

-*Scars/notches edge:* 1-neat 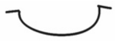 2-slightly irregular 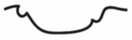 3-very irregular 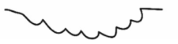

-*Scars/nocthes inner surface 1*: 1-even 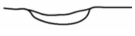 2-uneven 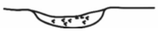

-*Scars/notches inner surface 2:* 1- faceted 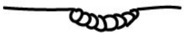 2-non-faceted 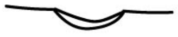

-*Negative bulb (conchoidal fracture)*: 1-present 2-absent

-*Morphology of the scars 1* (the most and the second most common morphology): 1-semi-circular 2-quadrangular 3-trapezoidal 4-triangular 5-half-moon 6-irregular (after Gonzalez Urquijo & Ibañez Estévez, 1994)

-*Initiation 1* (the most common initiation): 1- narrow 2-wide (Rots, 2010)

Proximal cross-section 1 and 2 (=cross-section of scars initiation – the most and the second most common): 1-crescent-break 2-break-shallow 3-shallow 4-steep

Distal cross-section 1 (=cross-section of scars termination – the most and the second most common): 1-crescent-break 2-feathered 3-step 4-hinge

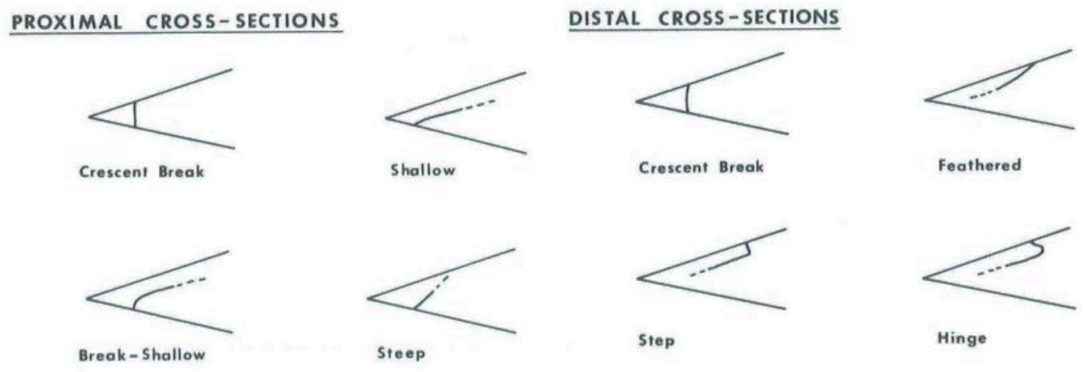

(After Tringham et al., 1974; Odell, 1977; Ho Ho Committee, 1979; Vaughan, 1985)

-*Scar width 1 and 2* (the most and the second most common width): 1-small: <0,5mm 2-medium size: 0,5-1mm 3-large: 1-2mm 4-very large: >2mm

-*Scar depth 1* and 2(the most and the second most common depth): 1-0,1-0,3mm 2-0,4-0,6mm 3-0,7-0,9mm 4-1,0-1,2mm 5-1,3-1,5mm 6-1,6-1,8mm

-*Width of largest notch/scar:*

-*Width of smallest notch/scar:*

-*Length of longest notch:*

-*Length of shortest notch:*

The measure of the depth of a scar was taken from its initiation to its termination, following the fracture axis (micro-scars present a conchoidal fracture, like the ventral face of a flake and removals). The measure of the width was taken in perpendicular to the fracture axis, where the scar is the widest.

Rounding

-*Position*: 1-on the very edge 2-ventral face 3-dorsal face 4-both faces 5-on ridges

-*Intensity*: 1-weak 2-medium 3-strong

-*Distribution*: 1-continuous 2-disontinuous

-*Shape*: 1-rounded 2-flat 3-faceted 4-rounded with a meplat (See Figure b)

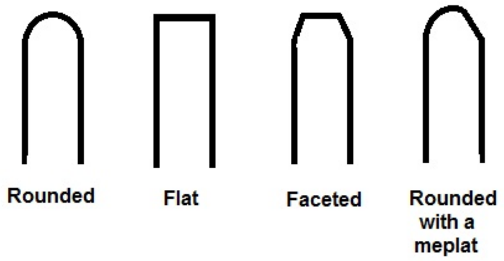

Figure h: Rounding shapes.

Macro-striations

-*Visibility*: 1-visible 2-non-visible

-*Direction to the edge*: 1-parallel 2-perpendicular 3-diagonal 4-diagonal crossed

HIGH POWER OBSERVATION

The following attributes were described in three different forms per tool: one for the ventral face, one for the dorsal face and one for the very edge (when relevant).

Micro-polish

-*Polish reflexivity* (the brilliance of the polish): 1-Dull 2-Greasy 3-Metallic 4-Bright (after Jensen, 1994; Pawlik, 1995 and Rots, 2010)

Although this assessment is subjective according to some authors (Gassin, 1996; Rots, 2010) we consider that it is valid to give an idea of the relative brightness of the polish created by different materials on stone tools made in the same raw material and studied by the same analyst.

-*Polish distribution*: 1-isolated spots 2-immediate edge 3-areal cover

-*Polish intrusion*: 1-Marginal (1-100µm) 2-Medium (100-500µm) 3-Invasive (>500µm) (after Jensen, 1994. The size of the different categories of polish intrusion are based on my corpus)

-*Degree of linkage 1 and 2* (the predominant linkage and the second most common): 1-scattered 2-linked 3-covering (Plisson, 1985; Jensen, 1994)

-*Polish topography 1 and 2*(the predominant linkage and the second most common topography): 1-slightly affected surface 2-domed polish 3-flat polish 4-fluted or rippled polish 5-fluid polish (After Plisson, 1985 and Jensen, 1994)

-*Polish texture*: 1-rough 2-smooth (Jensen, 1994)

-*Regularity of the limit between the polished zone and the unaffected one*: 1-regular 2-irregular (See Figure c)

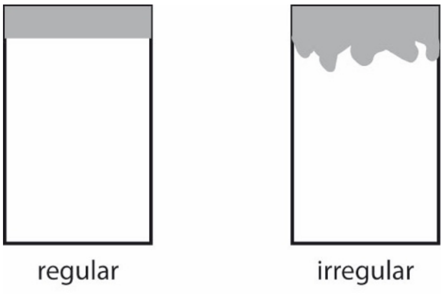

Figure i: Regularity of the limit between the polished zone and the unaffected one.

-*Appearance of the limit between the polished zone and the unaffected one*: 1-clear 2-progressive (Based on Gassin, 1996)

-*Amount of micro-pits*: 1-none 2-few 3-numerous

-*Polish bevel*: 1-present 2-absent

This field refers to the very edge which becomes fat due to heavy abrasion, forming a sort of bevel to the tool. It often corresponds to flat rounding (visible at low magnification, under a stereomicroscope and sometimes even to the naked eye).

-*Direction of the polish to the edge*: 1-undetermined 2-longitudinal 3-transversal 4-transverse

Striations

Mansur-Franchomme (1986) established a typology of striations based widely on observations undertaken with Scanning Electron Microscope. We conducted our analyses of micro-wear with optical microscopes and at lower magnifications than Mansur-Franchomme (5X to 200X vs. 450X, 900X, thousands of times). It was therefore difficult to use her classification, not knowing how some of the striation types she describes would look like at relatively low magnifications under a reflexive microscope. Besides, some of her types, such as "*stries comblées*", meaning filled striations, imply that the silica-gel theory of polish formation is accepted. We preferred to describe attributes of the striations observed rather than to employ *a priori* (or even *a posteriori*) types. The following fields are based on the work of Pawlik (1995, recording form), Keeley (1980), Plisson (1985),Vaughan (1985),

Mansur-Franchomme (1986), Van Gijn (1989), Gonzalez Urquijo & Ibañez Estévez (1994), Jensen (1994), Gassin, 1996; Borel (2010, 2012) and Rots (2010) and on our corpus.

-*Amount*: 1-none 2-one 3-a few 4-medium 5-many

-*Morphology*: 1-straight 2-curved 3-both

-*Width*: 1-narrow < 5µm 2- medium 5-10µm 3- large >10µm

-*Length*: 1-short: <50µm 2-medium 50-200µm 3-long >200µm

-*Continuity*: 1-continuous 2-interrupted 3-doted

- *Reflexivity*: 1- dark 2- bright

-*Depth*: 1-deep 2-shallow 3-superficial (Vaughan, 1985)/striation-like polish (Rots, 2010)

The term superficial has been used by different authors to define both grooves of a small depth (for instance Mansur-Franchomme, 1986) and linear polish (for instance Vaughan, 1985). To avoid any confusion, we chose to use the word shallow to characterize the striae of shallow depth and superficial / striation-like polish for elongated polish.

- *Individuality*: 1-separated 2-brush-stroke (See Figure d)

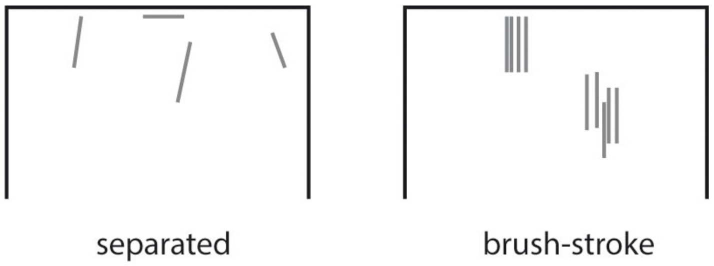

Figure j: Separated and brush-stroke striations.

-*Direction to the cutting edge*: 1-parallel 2-perpendicular 3-diagonal 4-diagonal crossed 5-random

References

Borel, A., 2010. Formes et fonctions au sein des industries lithiques de la fin du Pléistocène et du début de l'Holocène en Asie du Sud-Est: un nouvel apport à la compréhension des comportements humains. Ph.D. Thesis, Muséum national d'Histoire naturelle, France.

Borel, A., 2012., Formes et fonctions au sein des industries lithiques de la fin du Pléistocène et du début de l'Holocène en Asie du Sud-Est, un nouvel apport à la compréhension des comportements humains. BAR. Archeopress, Oxford.

Gassin, B., 1996. Evolution socio-économique dans le Chasséen de la grotte de l'Eglise supérieure (Var). Apport de l'analyse fonctionnelle des industries lithiques. CNRS Editions, Paris.

Gonzales Urquijo, J.E., Ibañez Estevez, J.J., 1994. Metodología de análisis funcional de instrumentos tallados en sílex. Universidad de Deusto ; Deustuko Unibertsitatea.

Ho Ho Committee, 1979. The Ho Ho classification and nomenclature committee report. Lithic Use-Wear Analysis. Academic Press, New York, 134-158.

Jensen, H. J., 1994. Flint tools and plant working: hidden traces of Stone Age technology: a use wear study of some Danish Mesolithic and TRB implements. Aarhus University Press, Denmark.

Keeley, L. H., 1980. Experimental Determination of Stone Tools. A Microwear Analysis. The University of Chicago Press, Chicago.

Lemonnier, P., 1992. Elements for an anthropology of technology. Museum of anthropology, University of Michigan, Ann Arbor.

Leroi-Gourhan, A., 1971 – 1st ed. 1943. L'homme et la matière. Ed. A. Michel, Paris.

Mansur-Franchomme, M. E., 1986. Microscopie du matériel lithique préhistorique : traces d'utilisation, altérations naturelles, accidentelles et technologiques : exemples de Patagonie. CNRS, Paris.

Odell, G. H., 1977. The application of micro-wear analysis to the lithic component of an entire prehistoric settlement: methods, problems and functional reconstructions. PhD Thesis, Harvard University.

Odell, G. H., 1980. Butchering with stone tools: some experimental results. Lithic Technology 9, 39-48.

Odell, G. H., Odell-Vereecken, F., 1980. Verifying the Reliability of Lithic Use-Wear Assessments by "Blind Tests". The Low-Power Approach. Journal of Field Archaeology 7, 87–120.

Pawlik, A. F., 1995. Die mikroskopische Analyse von Steingeräten: Experimente – Auswertungsmethoden – Artefaktanalyse. Urgeschichtliche Materialhefte 10, Archaeologica Venatoria, Tübingen.

Plisson, H., 1985. Etude fonctionelle des outillages lithiques prehistoriques par l'analyse des micro-usures, recherche methodologique et archeologique. Ph.d. Thesis, Université de Paris I, France.

Rots, V., 2010. Prehension and hafting traces on flint tools: a methodology. Leuven University Press.

Tringham, R., Cooper, G., Odell, G., Voytek, B., Whitman, A., 1974. Experimentation in the Formation of Edge Damage: A New Approach to Lithic Analysis. Journal of Field Archaeology 1, 171–196.

Van Gijn, A. L., 1989. The wear and tear of flint principles of functional analysis applied to Dutch neolithic assemblages. Analecta Praehistorica Leidensia 22, Institute of Prehistory, University of Leiden.

Vaughan, P., 1985. Use-wear analysis of flake stone tools. University of Arizona Press.

Details of the experiments: variables and resulting use-wear

1- **Thinning**
2- **Scraping the epidermis**

Videos of the experiments can be watched on www.plantuseinseasia.net

***Schizostachyum* cf. *lima* – Thinning** - See video EXP-19

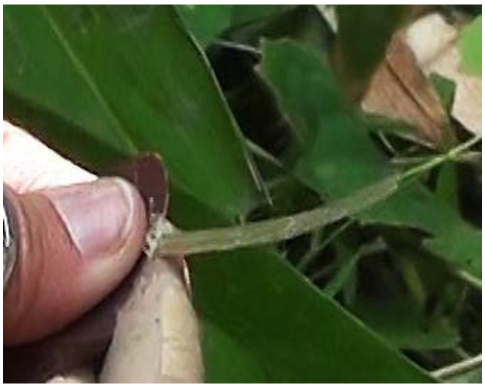

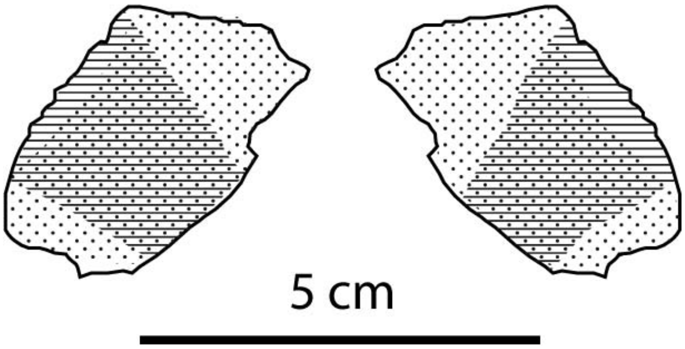

Time: **10 minutes**	Date: **8-25-1012**	Weather: **dry**

Contact material	Freshness: **fresh**			Degree of moisture: **rather dry**	
	Part of the plant: **inner fibers of bamboo culm segments**			Dimension: **L:30-50cm, w:5mm, h:2mm**	
	Degree of hardness: **semi-soft**			Degree of penetrability: **medium to penetrate**	
Experimental tool	ID: **EX12-20**	Blank form: **flake**	Type of flake: **short flake**	Cortex: **absent**	Fragmentation: **non fragmented**
	Technological length: **31mm**		Technological width: **40mm**	Maximum length: **36mm**	Maximum width: **31mm** / Maximum thickness: **4,4mm**
	Location of used area: **left edge**		Type of used area: **unretouched**	Length of used area: **30,6mm**	Edge angle: **19°**
	Edge profile: **biconcave**		Edge form: **straight**	Edge torsion: **none**	Number of torsions: -
	Reason for this choice: **The thinness of this flake, its small size and its straight edge with a low angle. All these characteristics make it sharp and able to slide between two layers of fibers.**				
Motion(s)	Part of the tool handled: **right part**			Mode of prehensility: **hands then hands protected with gloves (after cutting myself)**	
	Type of motion: **thinning down**			Type of percussion: **resting**	
	Contact face: **Both faces but mostly the dorsal.** (As the two faces are very flat, while focusing on my task, I switched without noticing it.)			Contact angle: **0-59°**	
	Direction of the motion to the active edge: **transversal (+crosswise when making a snick, but marginal)**			Direction of the motion to fibers of the plant: **longitudinal**	
	Way of the motion: **unidirectional (+bidirectional when making a snick, but marginal)**			Tool efficiency to achieve the task: **very good**	
Comments	After a few minutes, I discovered that the thinning down motion was more efficient with a higher contact angle. Therefore, it switched from 0-29° to 30-59°.				

MICRO-WEAR

EX12-20

Schizostachyum cf. *lima* (fresh)

Thinning

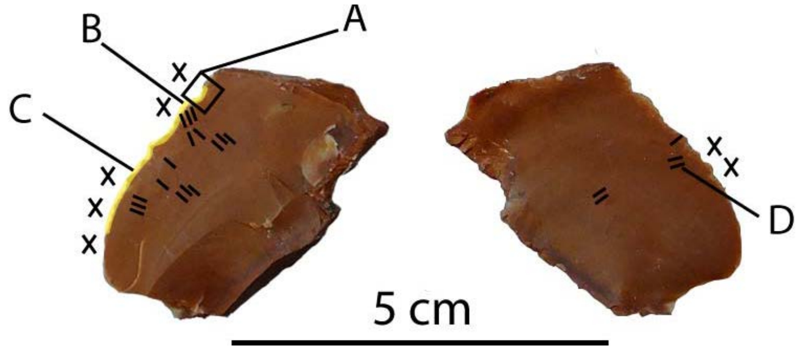

Micro-scars: Many micro-scars are visible all along the edge, predominantly on the dorsal face. Their distribution is continuous and their orientation is perpendicular and transverse bidirectional. They are half-moon shaped for most of them, although some small ones are trapezoidal. Their proximal cross-sections are crescent-break or steep and their distal ones crescent-break and hinge. The width of most of them is medium (0,5 to 1mm) and their depth from 0,4 to 0,6mm, many being 0,1-0,3mm (crescent-break). One is particularly large (picture A) and shows a second edge row, a series of smaller micro-scars on its initiation. It makes it look like a very small notch and is probably due to the fact that the fine bamboo segments were very much in contact with this spot. The shape of this "notch" makes it perfect to corner the fibers to thin down, the action damaging it even more.

Micro-polish: The dorsal face presents a very developed polish on its very edge. It corresponds to a very flat polish cut by dark deep striations perpendicular to the edge or even a fluted polish. It is very marginal, a dull polish affecting the dorsal face itself more intrusively.

Striations: Numerous brush stroke striations, made of alternating striation-like polish and dark grooves, are visible on both faces, predominantly on the dorsal one. They are cross-wise and perpendicular to the edge and form a few sets, parallel between themselves, oriented in the same direction and creating long lines. These long lines correspond to my opinion to the places in contact with the bamboo strips fibers

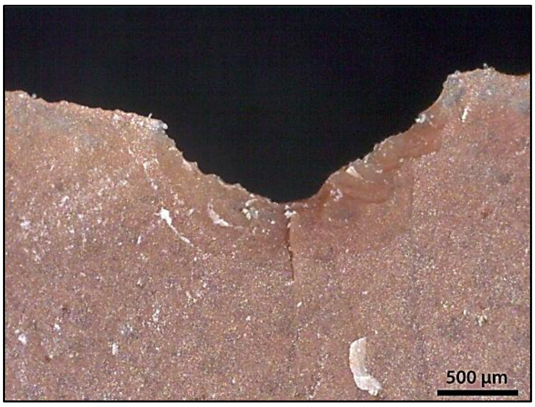

EX12-20A: Large micro-scar with smaller ones on its initiation (second edge row).

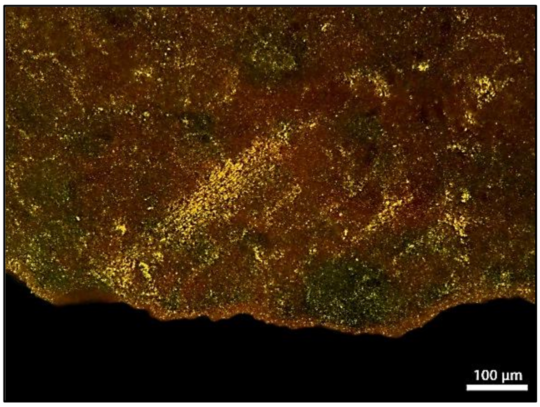

EX12-20B: Brush stroke striations cross-wise to the edge and parallel between themselves.

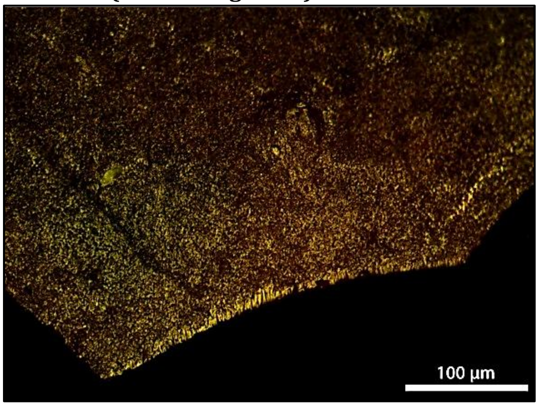

EX12-20C: Very developed polish along the very edge with directional markers perpendicular to it (dark deep micro-striations or undulation of fluted polish).

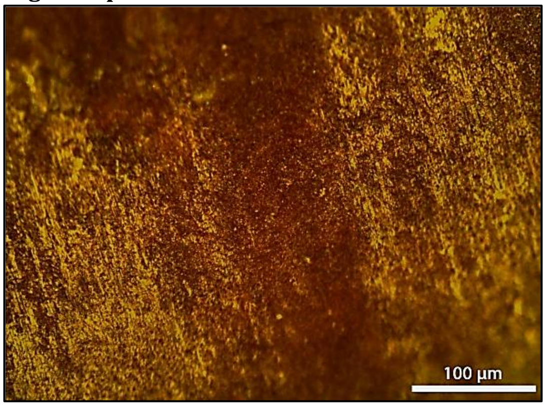

EX12-20D: Brush-stroke micro-striations on a ripple, cross-wise to the edge.

Schizostachyum **cf.** ***lima*** **- Thinning -** See video EXP-20

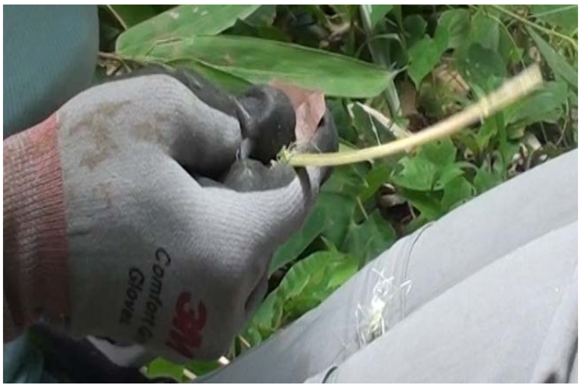

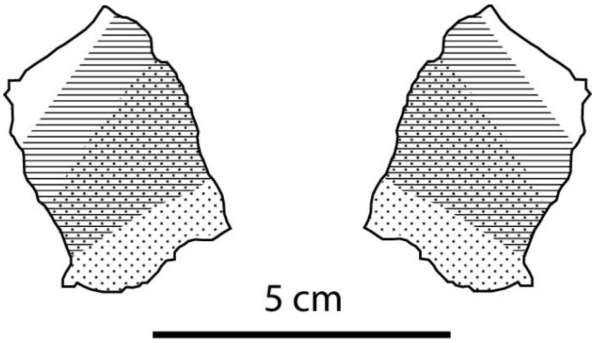

Time: **30 minutes**			Date: **8-26-2012**		Weather: **dry**
Contact material	Freshness: **fresh**			Degree of moisture: **rather dry**	
	Part of the plant: **inner fibers of bamboo culm segments**			Dimension: **L:30-50cm,w:5mm, h:2mm**	
	Degree of hardness: **semi-soft**			Degree of penetrability: **medium to penetrate**	
Experimental tool	ID: **EX12-19**	Blank form: **flake**	Type of flake: **long flake**	Cortex: **absent**	Fragmentation: **non fragmented**
	Technological length: **44mm**		Technological width: **29mm**	Maximum length: **39mm**	Maximum width: **29mm** / Maximum thickness: **4,2mm**
	Location of used area: **right edge**		Type of used area: **unretouched**	Length of used area: **39,3mm**	Edge angle: **19°**
	Edge profile: **plano-concave**		Edge form: **straight**	Edge torsion: **minimal**	Number of torsions: **one**
	Reason for this choice: **The flat ventral face, the straight edge with a low angle. It makes this flake sharp and thin enough to slide between two layers of fibers.**				
Motion(s)	Part of the tool handled: **left part**			Mode of prehensility: **hands protected with gloves**	
	Type of motion: **thinning down**			Type of percussion: **resting**	
	Contact face: **mostly ventral. Both faces are in contact when layers of fibers are taken off (Operation 5B).**			Contact angle: **0-59°**	
	Direction of the motion to the active edge: **transversal (+crosswise when making a snick, but marginal)**			Direction of the motion to fibers of the plant: **longitudinal**	
	Way of the motion: **unidirectional (+bidirectional when making a snick, but marginal)**			Tool efficiency to achieve the task: **very good**	
Comments	-				

MICRO-WEAR

EX12-19

Schizostachyum cf. *lima* (fresh)

Thinning

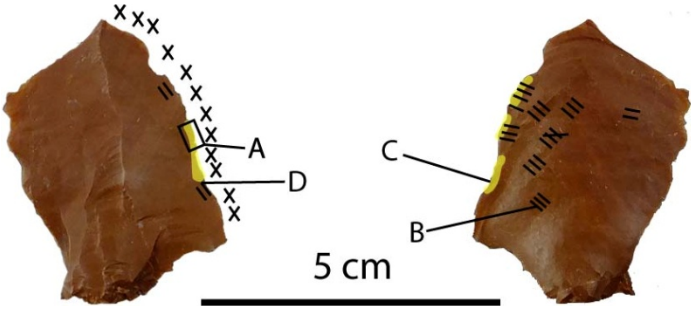

Micro-scars: Many micro-scars are present all along the edge on the dorsal face. Their distribution is continuous and their direction perpendicular to the edge. They are half-moon shaped and crescent-break, a few of them being steep-hinge. Their width is small (<0,5µm) to medium (0,5-1mm) and their depth from 0,1 to 0,3mm.

Micro-polish: The dorsal face exhibits a dull to greasy polish along the edge, intruding perpendicularly and the ventral face a very developed polish at one spot of the very edge. The latter is domed to fluted, the undulations of the latter being perpendicular to the rim. It shows a few micro-pits and its limit is rather clear. It is possible that polish had developed on other parts of the edge as well but have been removed by micro-chips. Other zones of the edge show indeed use-wear in the form of brush-stroke striations (see below and mapping). Further inside the ventral face, long trails of polish can be seen (greasy to metallic). It is slightly affecting the topography of the tool and at some places where it is more developed, it forms domed components. These long polish trails are more or less perpendicular to the edge (perp. and cross-wise) and are actually made of a superposition of brush-stroke striations.

Striations: As mentioned just above, the ventral face shows numerous brush-stroke striations made of alternating striation-like polish and dark grooves which form here long trails of polish, perpendicular to the edge. They can be associated to thinning down the bamboo strips (unidirectional motion transversal to the edge).

The dorsal face presents along the edge a few striation-like polishes parallel to it and to the edge. These striations can be related to snick making (bidirectional motion longitudinal to the edge).

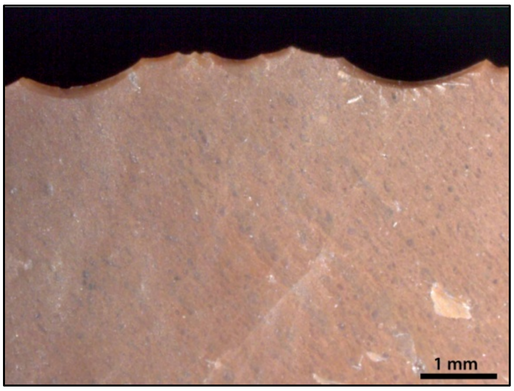

EX12-19A: Crescent-break micro-scars.

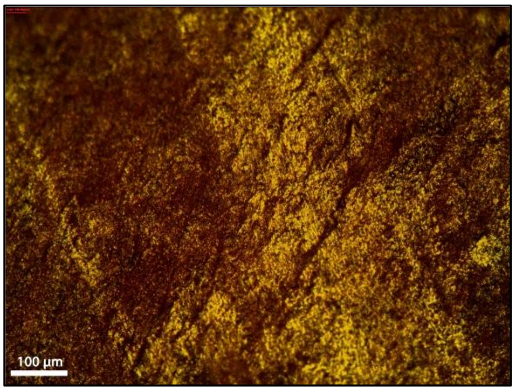

EX12-19B: long trails of polish made of a superposition of brush-stroke striations.

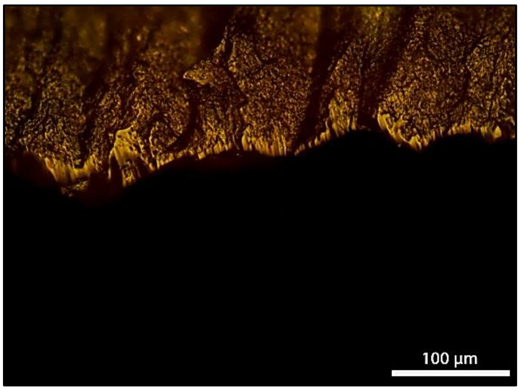

EX12-19C: Fluted polish on the very edge. Its undulations are perpendicular to the rim.

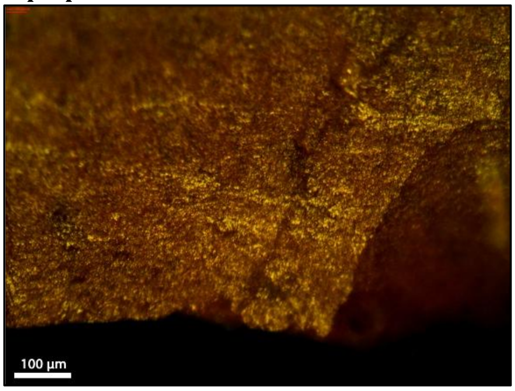

EX12-19D: Striation-like polishes parallel to the edge which are cut by a micro-scar.

Schizostachyum cf. *lima* - **Thinning** - See video EXP-21

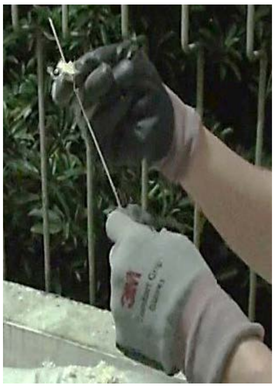

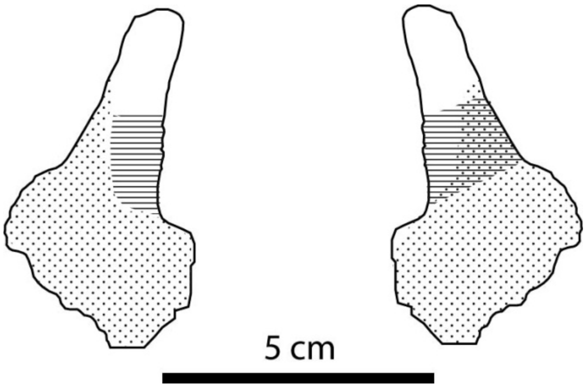

Time: **10 minutes**	Date: **9-7-2012**	Weather: **dry**

Contact material	Freshness: **dry**	Degree of moisture: **very dry**
	Part of the plant: **inner fibers of bamboo culm segments**	Dimension: **L:38cm, l:6mm, h:2mm**
	Degree of hardness: **semi-soft**	Degree of penetrability: **medium to penetrate**

Experimental tool	ID: **EX12-22**	Blank form: **flake**	Type of flake: **long flake**	Cortex: **<1/3**	Fragmentation: **incomplete width**	
	Technological length: **58mm**		Technological width: **33mm**	Maximum length: **50mm**	Maximum width: **35mm**	Maximum thickness: **6mm**
	Location of used area: **distal part of the right edge**		Type of used area: **unretouched**	Length of used area: **34mm**	Edge angle: **34°**	
	Edge profile: **biplane**		Edge form: **straight**	Edge torsion: **minimal**	Number of torsions: **one**	
	Reason for this choice: **The biplane profile of this flake makes it good to slide down the bamboo segment (flat surface as well). Its distal protuberance makes it easy to hold without touching this active part.**					

Motion(s)	Part of the tool handled: **proximal and left part**	Mode of prehensility: **hands protected with gloves**
	Type of motion: **thinning down**	Type of percussion: **resting**
	Contact face: **mostly ventral. Both faces are in contact when layers of fibers are taken off (Operation 5B).**	Contact angle: **0-89°**
	Direction of the motion to the active edge: **transversal (+crosswise when making a snick, but marginal)**	Direction of the motion to fibers of the plant: **longitudinal**
	Way of the motion: **unidirectional (+bidirectional when making a snick, but marginal)**	Tool efficiency to achieve the task: **very good**
Comments	-	

MICRO-WEAR

EX12-22

Schizostachyum cf. *lima* (dry)

Thinning

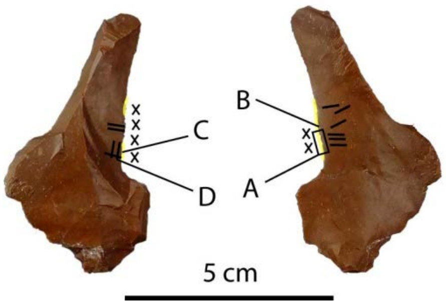

MICRO-SCARS: The active edge presents a few micro-scars, predominantly on dorsal face. They are localized and their distribution is wide. They are perpendicular to the edge and half-moon shaped. Their initiations are wide, crescent-break and break-shallow. Their distal cross-sections are crescent-break and feathered. They are of medium width (0,5-1mm) and very short depth (0,1-0,3mm).

MICRO-POLISH: The most developed polish is located on the very edge. Its topography is flat at some spots and domed at others. Some of these domed components are stretched out and oriented perpendicularly to the edge. The limit of the latter is lace-like than blur, it presents micro-pits and a smooth texture. The two faces show a dull polish.

STRIATIONS: The flake shows different kinds of striations, both perpendicular, related to thinning down and parallel, due to snick making. The spots of flat polish on the very edge present short dark deep and narrow striations, perpendicular to the rim. Both faces show striation-like polishes and brush-stroke striations. The width and length of these are varied, some being very large and long while other ones are of medium width and length.

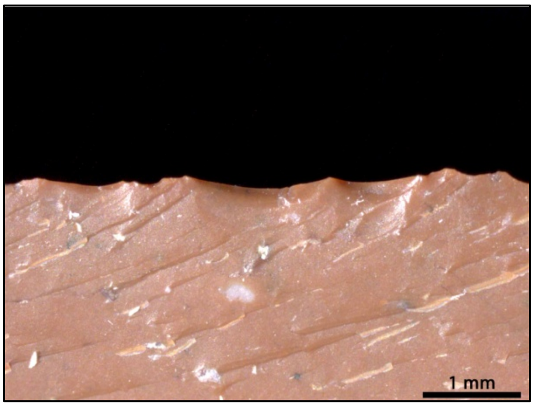

E12-22A: Crescent-break micro-scars.

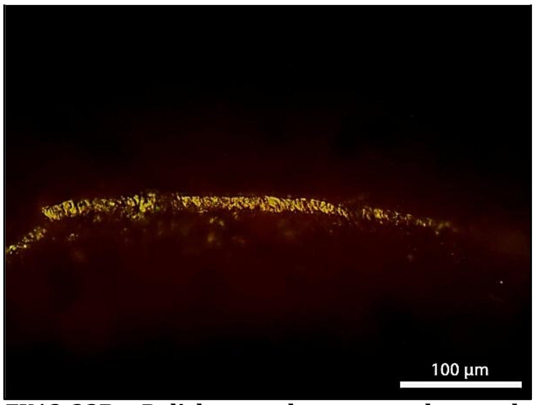

EX12-22B: Polish on the very edge and dark striations perpendicular to it.

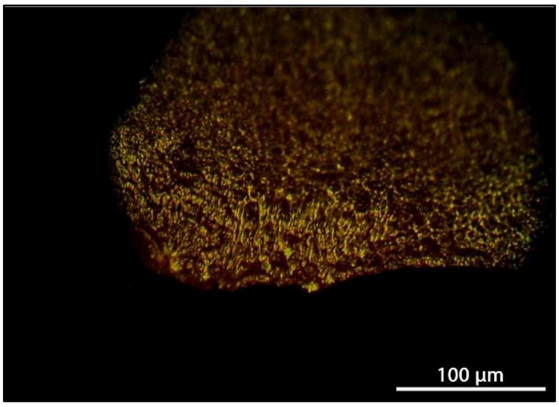

EX12-22C: Domed polish components. Some are stretched out perpendicularly to the edge.

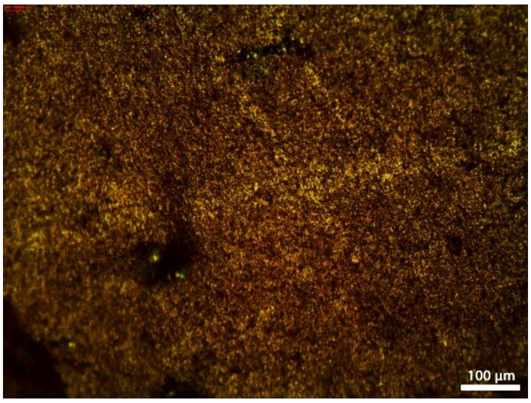

EX12-22D: Striation-like polishes. Some are perpendicular to the edge (thinning down) and other ones parallel to it (snick making).

Schizostachyum cf. *lima* - **Thinning** - See video EXP-22

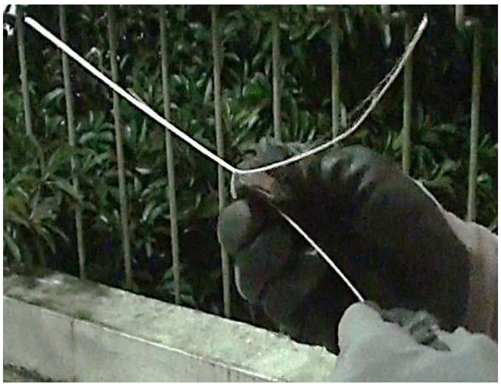

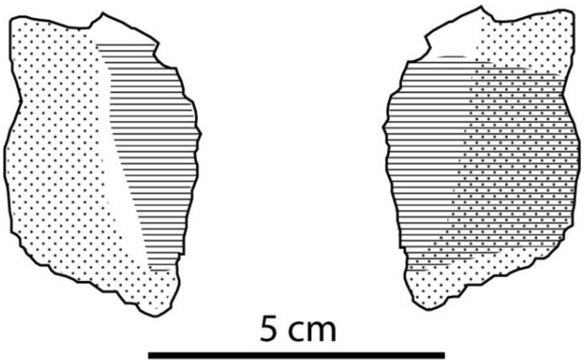

Time: **30 minutes**			Date: **9-7-2012**		Weather: **dry**
Contact material	Freshness: **dry**			Degree of moisture: **very dry**	
	Part of the plant: **inner fibers of bamboo culm segments**			Dimension: **L: 29 to 37cm, l:5mm, h:2mm**	
	Degree of hardness: **semi-soft**			Degree of penetrability: **medium to penetrate**	
Experimental tool	ID: **EX12-21**	Blank form: **flake**	Type of flake: **long flake**	Cortex: **absent**	Fragmentation: **Incomplete length**
	Technological length: **43mm**		Technological width: **31mm**	Maximum length: **41mm**	Maximum width: **36mm** / Maximum thickness: **9mm**
	Location of used area: **right edge**		Type of used area: **unretouched**	Length of used area: **43mm**	Edge angle: **35°**
	Edge profile: **plano-concave**		Edge form: **convex**	Edge torsion: **none**	Number of torsions: -
	Reason for this choice: **because of its thinness, relatively low angle and flat ventral face this flake is able to slide down on the bamboo inner surface.**				
Motion(s)	Part of the tool handled: **left part**			Mode of prehensility: **hands protected with gloves**	
	Type of motion: **thinning down**			Type of percussion: **resting**	
	Contact face: **mostly ventral. Both faces are in contact when layers of fibers are taken off (sub-operation 5B).**			Contact angle: **0 to 89°**	
	Direction of the motion to the active edge: **transversal (+crosswise when making a snick, but marginal)**			Direction of the motion to fibers of the plant: **longitudinal**	
	Way of the motion: **unidirectional (+bidirectional when making a snick, but marginal)**			Tool efficiency to achieve the task: **very good**	
Comments	-				

MICRO-WEAR

EX12-21

Schizostachyum cf. *lima* (dry)

Thinning

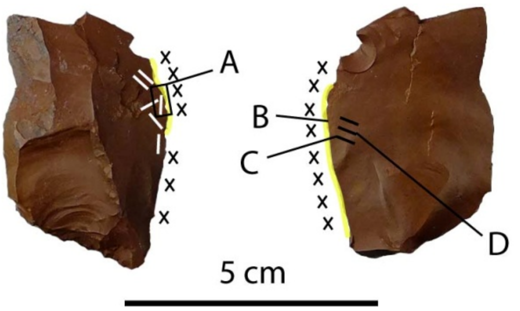

MICRO-SCARS: The flake presents a medium amount of micro-scars on both faces. They are oriented perpendicularly to the edge for most of them. They are half-moon shaped and semi-circular. Their initiations are wide, their cross-section being crescent-break and break-shallow. The first are crescent-break terminated while the second are feathered. The latter are small (<0,5mm on 0,1-0,3mm) and the crescent-break quite large (0,5-1mm and in one case, more than 2mm), their depth being very short (0,1-0,3mm).

MICRO-POLISH: The very edge presents a very bright smooth polish. Its topography is flat, its linkage covering, and its limit very clear. The dorsal and ventral faces show a dull polish of medium intrusion that affects slightly the topography of the rock.

STRIATIONS: The developed flat polish of the very edge shows dark deep short striations of medium width. They are oriented perpendicularly to the edge.
Both faces present brush-stroke and striation-like polish, that quite long and large. They are oriented in diagonal (related to thinning down) and in parallel (related to snick making) to the rim.

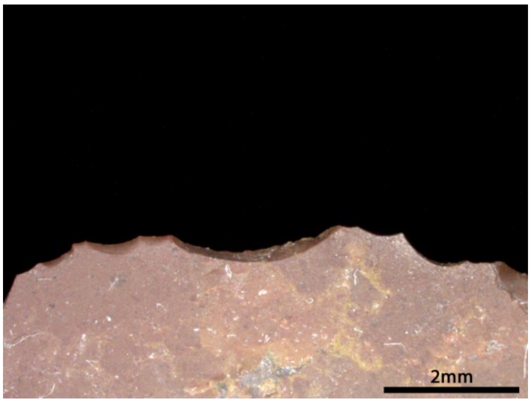

EX12-21A: Crescent-break micro-scars.

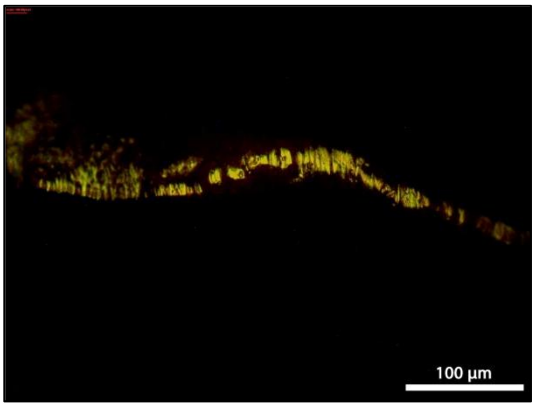

EX12-21B: Bright polish on the very edge and dark striations perpendicular to it.

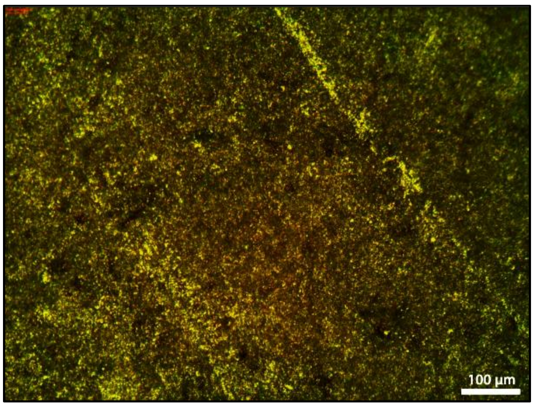

EX12-21C: Long striation-like polishes, cross-wise to the edge and parallel to each other.

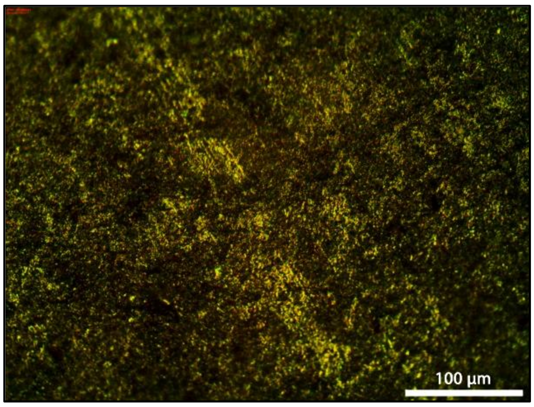

EX12-21D: Brush-stroke striations cross-wise to the edge.

***Dinochloa luconiae* - Thinning-** See video EXP-31

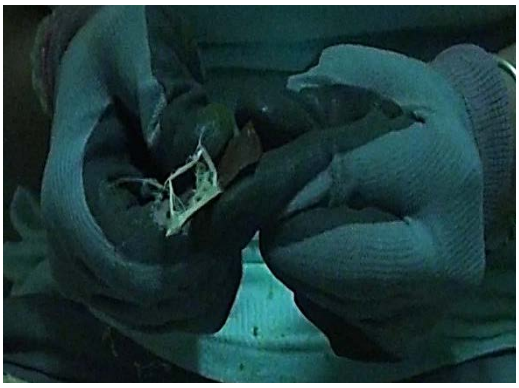

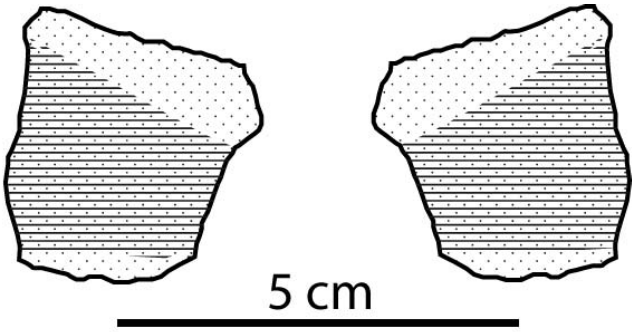

Time: **10 minutes**				Date: **8-26-2012**		Weather: **dry**
Contact material	Freshness: **fresh**			Degree of moisture: **rather moist**		
	Part of the plant: **inner fibers of bamboo stem segments**			Dimension: **L:25-30cm, w:4-9mm, thinckness:4mm**		
	Degree of hardness: **semi-hard**			Degree of penetrability: **medium to penetrate**		
Experimental tool	ID: **EX12-40**	Blank form: **flake**	Type of flake: **short flake**	Cortex: **absent**	Fragmentation: **non fragmented**	
	Technological length: **30mm**		Technological width: **31mm**	Maximum length: **29mm**	Maximum width: **29mm**	Maximum thickness: **9mm**
	Location of used area: **a)left and b)right edges**		Type of used area: **a) and b) unretouched**	Length of used area: **a)26mm b)14mm**	Edge angle: **a) 33° b)42°**	
	Edge profile: **a)biplane b)convexo-concave**		Edge form: **a)irregular b)straight**	Edge torsion: **a)minimal b)none**	Number of torsions: **a)one b)none**	
	Reason for this choice: **The thinness of this flake and its small size make it possible to insert it between two layers of fibers.**					
Motion(s)	Part of the tool handled: **all**			Mode of prehensility: **hands protected with gloves**		
	Type of motion: **thinning down**			Type of percussion: **resting**		
	Contact face: **ventral**			Contact angle: **0-59°**		
	Direction of the motion to the active edge: **transversal (+crosswise when making a snick, but marginal)**			Direction of the motion to fibers of the plant: **longitudinal**		
	Way of the motion: **unidirectional (+bidirectional when making a snick, but marginal)**			Tool efficiency to achieve the task: **good**		
Comments	As the worked material was quite hard, the left hand sometimes helped the right to push the flake upward.					

MICRO-WEAR

EX12-40

Dinochloa luconiae (fresh)

Thinning

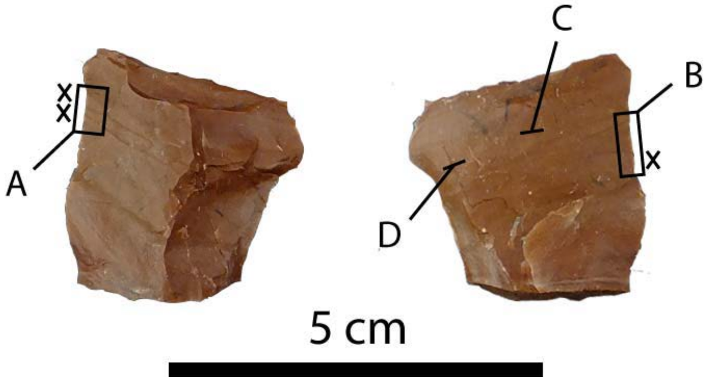

Micro-scars: Only the left edge is affected by a few micro-scars. They are quite localized, distributed in distinct patches. They are predominant on the dorsal face and their orientation is perpendicular and transverse bidirectional to the edge. Their shapes are half-moon and quadrangular. Their initiations are wide, crescent-break and break-shallow while the distal cross-sections are crescent-break or hinge. They are of medium (0,5-1mm) to large (1-2mm) width and small to medium depth (01-0,6mm).

Micro-polish: No polish was observed.

Striations: Only a few striation-like polishes are visible on the ventral face. One is slightly curved while the others are straight. Their length is long to medium and their width medium. They are quite discreet and perpendicular to the edge.

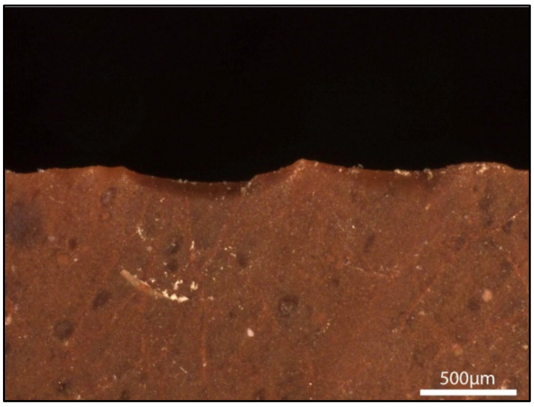

EX12-40A: Two very small micro-scars.

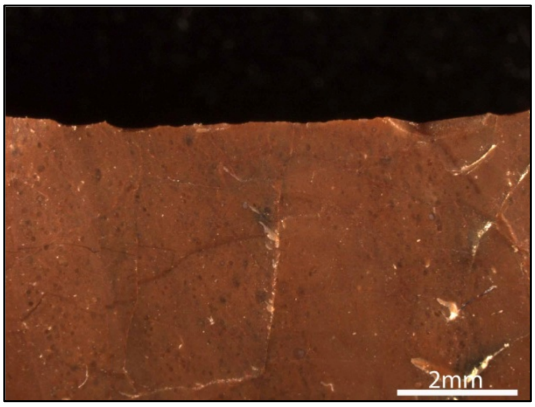

EX12-40B: Almost no micro-scars along the edge.

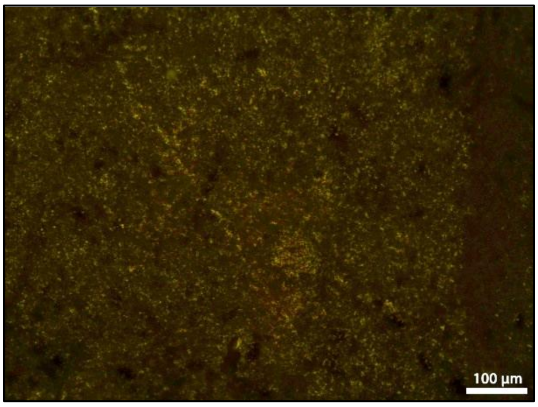

EX12-40C: Striation-like polish perpendicular to the edge.

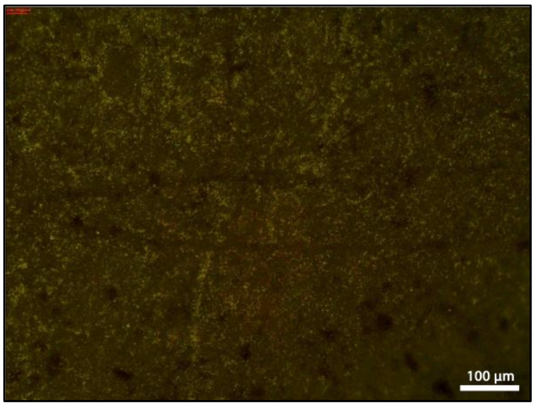

EX12-40D: Striation-like polish perpendicular to the edge.

***Dinochloa luconiae* - Thinning-** See video EXP-32

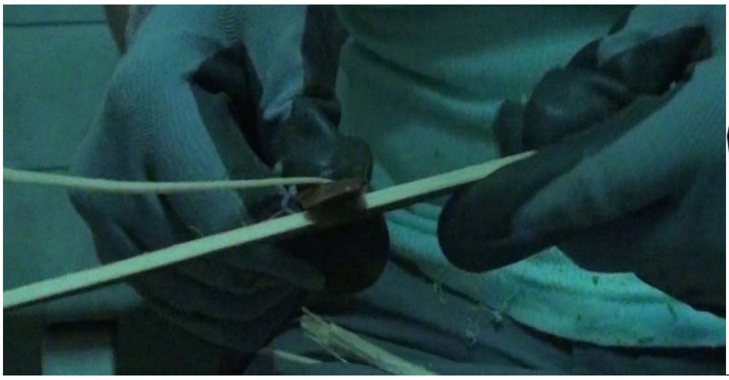

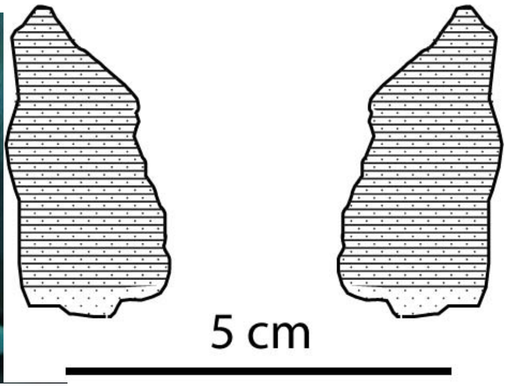

Time: **30min**			Date: **8-26-2012**	Weather: **dry**	
Contact material	Freshness: **fresh**		Degree of moisture: **rather moist**		
	Part of the plant: **inner fibers of bamboo stem segments**		Dimension: **L:25-30cm, w:4-5mm, thinckness:4mm**		
	Degree of hardness: **semi-hard**		Degree of penetrability: **medium to penetrate**		
Experimental tool	ID: **EX12-38**	Blank form: **flake**	Type of flake: **long flake**	Cortex: **<1/3**	Fragmentation: **non fragmented**
	Technological length: **37mm**	Technological width: **19mm**	Maximum length: **37mm**	Maximum width: **19mm**	Maximum thickness: **4mm**
	Location of used area: **a)left and b)right edges**	Type of used area: **a) and b) unretouched**	Length of used area: **a) 31mm b) 28mm**	Edge angle: **a) and b) 28-30°**	
	Edge profile: **a) and b) biplane**	Edge form: **a) and b) irregular**	Edge torsion: **a) and b) none**	Number of torsions: -	
	Reason for this choice: **The thinness of this flake, its small size and its biplane profile and low angles. All these characteristics make it sharp and able to slide between two layers of fibers.**				
Motion(s)	Part of the tool handled: **all**		Mode of prehensility: **hands protected with gloves**		
	Type of motion: **thinning down**		Type of percussion: **resting**		
	Contact face: **both faces**		Contact angle: **0-59°**		
	Direction of the motion to the active edge: **transversal (+crosswise when making a snick, but marginal)**		Direction of the motion to fibers of the plant: **longitudinal**		
	Way of the motion: **unidirectional (+bidirectional when making a snick, but marginal)**		Tool efficiency to achieve the task: **very good**		
Comments	-				

MICRO-WEAR
EX12-38
Dinochloa luconiae (fresh)
Thinning

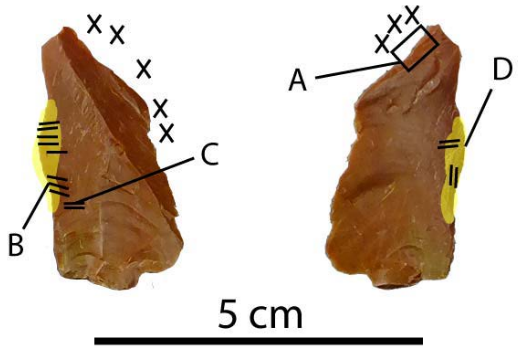

Micro-scars: A few micro-scars are visible on both faces of the right edge. They are distributed in distinct patches, wide in between and half-moon shaped. Their initiations are wide and both cross-sections crescent-break. They are small (<0,5mm) to medium (0,5mm-1mm) and not intrusive (0,1-0,3mm).

Micro-polish: A dull polish is present on both faces of the left edge, its intrusion from the edge being invasive. It is only slightly affecting the topography of the rock and its linkage is scattered. The limit between the polished surface and the unaffected one is blur (progressive) and irregular.
The very edge also shows a dull polish slightly affecting the surface of the rock.
The right part of the dorsal face which is cortical shows a developed polish, but this is due to the history of the block of jasper so it is not considered here.

Striations: Many striations were observed, mostly on the dorsal face, some being striation-like polishes, and others brush-stroke striations, made of alternating striation-like polishes and darker grooves. They are large, of medium length and straight and form large sets. Most are perpendicular and cross-wise to the edge (correlated with thinning down) and a few are parallel to it (correlated with snick making).

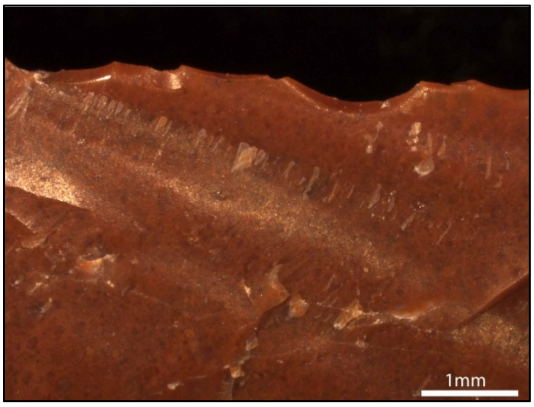

EX12-38A: Crescent-break micro-scars.

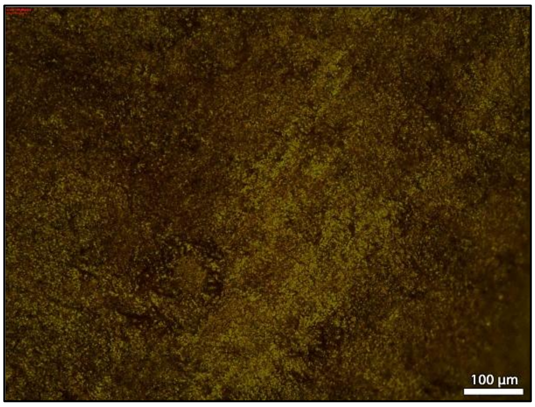

EX12-38B: Brush-stroke striations perpendicular to the edge and dull polish.

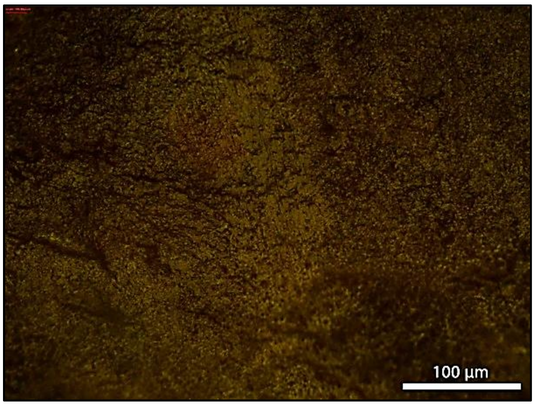

EX12-38C: Detail of brush-stroke striations: alternating striation-like polishes and darker and deeper groves.

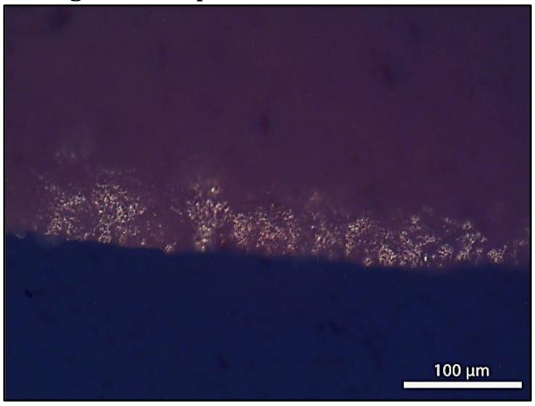

EX12-38D: On the very edge: weak and dull polish slightly affecting the topography of the rock.

Dinochloa luconiae - **Thinning**- See video EXP-39

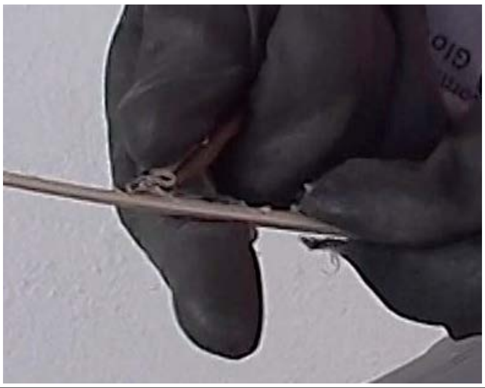

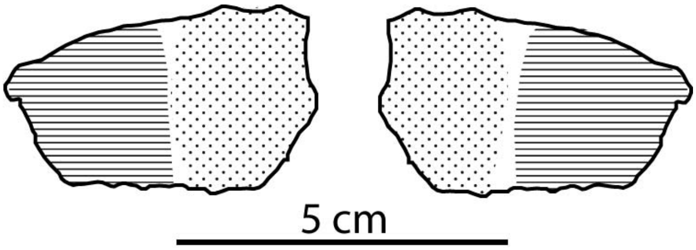

Time: **10 minutes**			Date: **6-24-2013**	Weather: **dry**	
Contact material	Freshness: **dry**		Degree of moisture: **very dry**		
	Part of the plant: **inner fibers of bamboo stem segments**		Dimension: **L:25cm, w:7mm**		
	Degree of hardness: **semi-hard**		Degree of penetrability: **medium to penetrate**		
Experimental tool	ID: **EX12-39**	Blank form: **flake**	Type of flake: **short flake**	Cortex: **absent**	Fragmentation: **proximal fragment**
	Technological length: **22mm**	Technological width: **45mm**	Maximum length: **45mm**	Maximum width: **27mm**	Maximum thickness: **6mm**
	Location of used area: **left edge**	Type of used area: **unretouched**	Length of used area: **13mm**	Edge angle: **27°**	
	Edge profile: **convexo-concave**	Edge form: **straight**	Edge torsion: **minimal**	Number of torsions: **one**	
	Reason for this choice: **because of its thinness, low angled and straight edge this flake is able to slide down on the bamboo inner surface and between two layers of fibers.**				
Motion(s)	Part of the tool handled: **right part**		Mode of prehensility: **hands protected with gloves**		
	Type of motion: **thinning down**		Type of percussion: **resting**		
	Contact face: **ventral**		Contact angle: **0-59°**		
	Direction of the motion to the active edge: **transversal**		Direction of the motion to fibers of the plant: **longitudinal**		
	Way of the motion: **unidirectional**		Tool efficiency to achieve the task: **medium**		
Comments	-				

MICRO-WEAR

EX12-39

Dinochloa luconiae

(dry)

Thinning

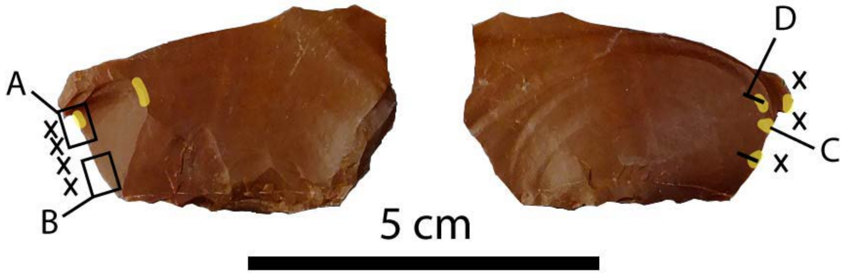

MICRO-SCARS: A few micro-scars are visible, predominantly on dorsal face. They are distributed in distinct patches and their orientation is perpendicular to the edge. They are mostly half-moon although some are quadrangular. Their proximal cross-sections are crescent-break and shallow and the distal ones crescent-break and feathered. Their size is small (<0,5mm wide) to medium (0,5-1mm wide) and their depth short (0,1-0,3mm).

MICRO-POLISH: The tool presents a dull polish on dorsal face, slightly affecting its topography. It is distributed in isolated spots and is invasive with a scattered linkage. The ventral face shows isolated spots of polish as well, but a little more developed. The reflexivity is greasy and the intrusion medium. Its linkage is scattered and the topography mostly only slightly affected, but some areas show smooth domed components. The limit between the polished area and the unaffected one is irregular and progressive.

STRIATIONS: The ventral face shows a few large and long striation-like polishes, some being straight and others curved. They are perpendicular to the edge.

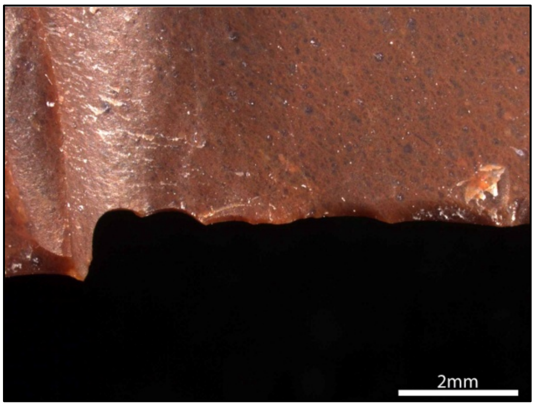

EX12-39A: Small crescent-break micro-scars.

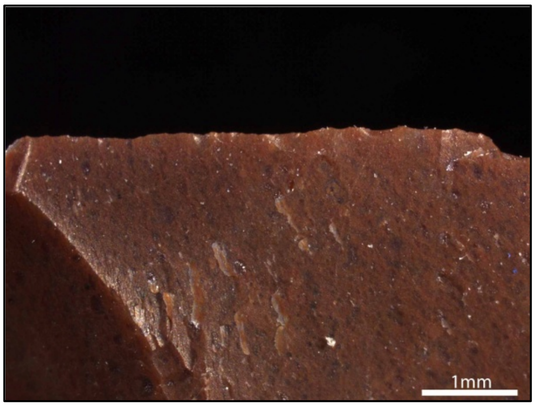

EX12-39B: Very small micro-scars.

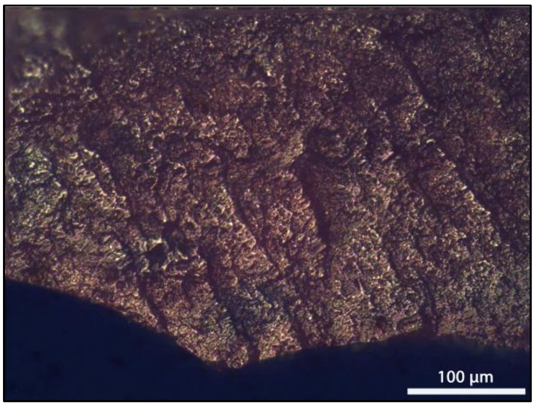

EX12-39C: Domed polish of scattered linkage.

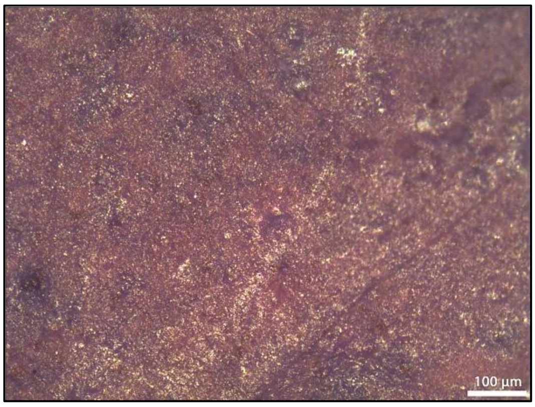

EX12-39D: Curved striation-like polish perpendicular to the edge.

Dinochloa luconiae **- Thinning-** See video EXP-40

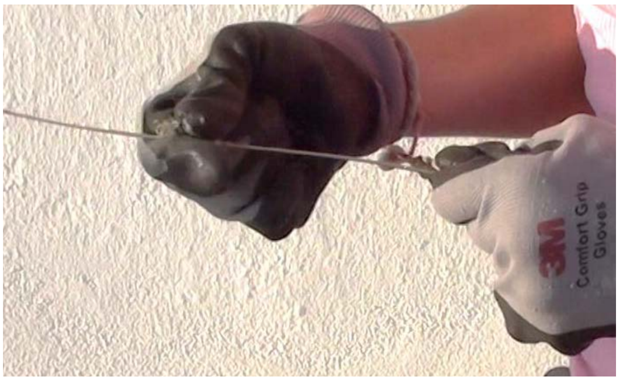

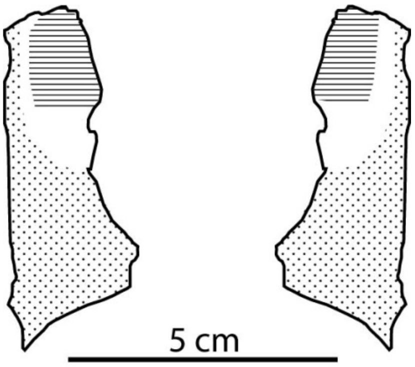

Time: **30 minutes**		Date: **6-24-2013**		Weather: **dry**	
Contact material	Freshness: **dry**			Degree of moisture: **very dry**	
	Part of the plant: **inner fibers of bamboo stem segments**			Dimension: **L:25-50cm, w:3-7mm**	
	Degree of hardness: **semi-hard**			Degree of penetrability: **medium to penetrate**	
Experimental tool	ID: **EX12-37**	Blank form: **flake**	Type of flake: **long flake**	Cortex: **absent**	Fragmentation: **Siret**
	Technological length: **59mm**		Technological width: **21mm**	Maximum length: **50mm**	Maximum width: **27mm** / Maximum thickness: **15mm**
	Location of used area: **distal part of the right edge**		Type of used area: **unretouched**	Length of used area: **16mm**	Edge angle: **16-30°**
	Edge profile: **biplane**		Edge form: **straight**	Edge torsion: **none**	Number of torsions: -
	Reason of this choice: **the straight biplane edge with a low angle. It makes this flake sharp and thin enough to slide between two layers of fibers. The long prehensive part is also an advantage.**				
Motion(s)	Part of the tool handled: **proximal part**			Mode of prehensility: **hands protected with gloves**	
	Type of motion: **thinning down**			Type of percussion: **resting**	
	Contact face: **ventral**			Contact angle: **0-59°**	
	Direction of the motion to the active edge: **transversal**			Direction of the motion to fibers of the plant: **longitudinal**	
	Way of the motion: **unidirectional**			Tool efficiency to achieve the task: **very good**	
Comments	-				

MICRO-WEAR

EX12-37

Dinochloa luconiae (dry)

Thinning

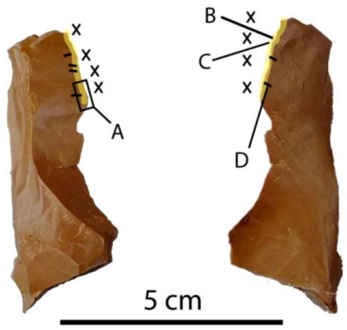

MICRO-SCARS: A few micro-scars are present on both faces but predominantly on the dorsal. They are continuous as well as perpendicular and transverse bidirectional to the edge. Their shapes are half-moon and quadrangular with wide initiations. Their proximal cross-sections are crescent-break and break-shallow and the distal ones crescent-break and hinge. They are of medium (0,5-1mm) to large (1-2mm) width and short depth (0,1-0,3mm).

MICRO-POLISH: Both faces show an invasive greasy polish that is slightly affecting the topography. Its limit is irregular and progressive. Along the very edge on ventral face is a more developed smooth polish, metallic and domed, that intrudes inside the ventral face perpendicularly to the edge. High elevations of the ventral face also show domed polish components. Some are stretched-out, forming lines perpendicular to the edge.

STRIATIONS: Many long brush-stroke pattern striations are visible on both faces. They are formed of striation-like polishes parallel between themselves and perpendicular to the edge. Each of these striation-like polishes is of medium width (5-10µm) but together they form very large sets. They are very superficial and don't affect much the topography of the rock.

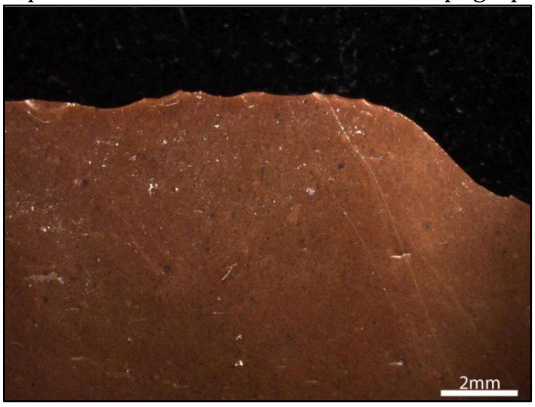

EX12-37A: Small crescent-break micro-scars.

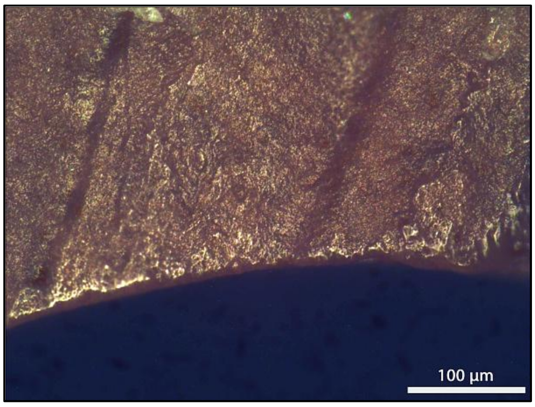

EX12-37B: Marginal polish along the very edge.

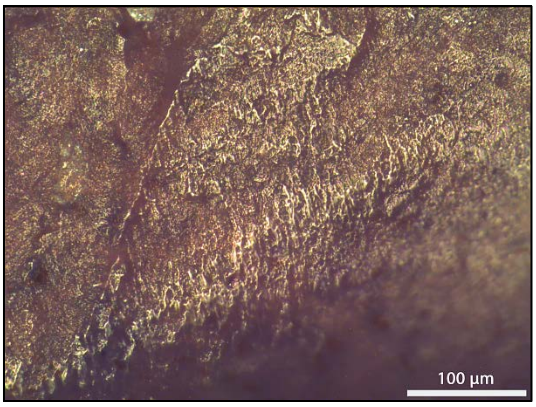

EX12-37C: Stretched-out domed polish components perpendicular to the edge.

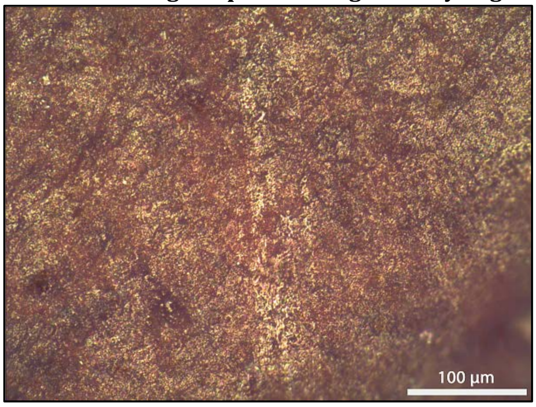

EX12-37D: Brush-stroke striations perpendicular to the edge.

Calamus merrillii - **Thinning**- See video EXP-64

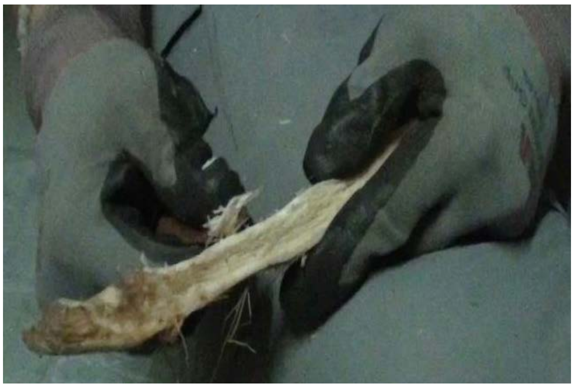

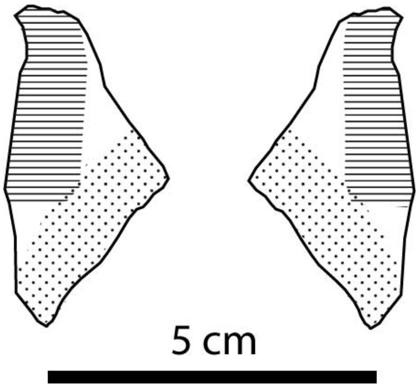

Time: **10 minutes**			Date: **9-1-2012**		Weather: **dry**
Contact material	Freshness: **fresh**			Degree of moisture: **rather dry**	
	Part of the plant: **split segment of caudex**			Dimension: **width: 1,5cm; thickness: 2cm; length: 15cm**	
	Degree of hardness: **demi-hard**			Degree of penetrability: **medium to penetrate**	
Experimental tool	ID: **EX12-55**	Blank form: **flake**	Type of flake: **long flake**	Cortex: **absent**	Fragmentation: **non fragmented**
	Technological length: **45mm**		Technological width: **25mm**	Maximum length: **45mm**	Maximum width: **25mm** / Maximum thickness: **6mm**
	Location of used area: **distal part of left edge**		Type of used area: **unretouched**	Length of used area: **25mm**	Edge angle: **21°**
	Edge profile: **biconcave**		Edge form: **straight**	Edge torsion: **none**	Number of torsions: -
	Reason for this choice: **The thinness of this flake and low edge angle made it look able to be inserted between two layers of fibers.**				
Motion(s)	Part of the tool handled: **right and proximal parts**			Mode of prehensility: **hands protected with gloves**	
	Type of motion: **thinning down**			Type of percussion: **resting**	
	Contact face: **mostly dorsal. Both faces are in contact when the tool is between fibers.**			Contact angle: **0-59°**	
	Direction of the motion to the active edge: **transversal (+crosswise when making a snick, but marginal)**			Direction of the motion to fibers of the plant: **longitudinal**	
	Way of the motion: **unidirectional (+bidirectional when making a snick, but marginal)**			Tool efficiency to achieve the task: **medium**	
Comments	-				

MICRO-WEAR
EX12-55
Calamus merrillii (fresh)
Thinning

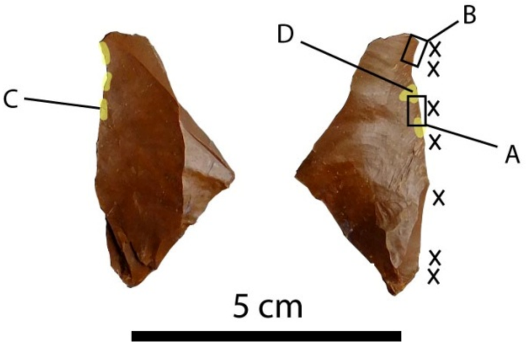

Gloss: A discreet gloss is visible under low-power microscope.
Micro-scars: A few micro-scars are present on ventral face. They are located all along the edge, distributed in distinct patches, wide in between. Their direction to the edge is perpendicular and transverse unidirectional. Their morphology is semi-circular and half-moon with wide initiations. Their proximal cross-sections are steep and crescent-break and the distal ones are feathered and crescent-break. They are small (<0,5mm in width) to medium (0,5-1mm in width) and short (0,1-0,3mm in length).
Micro-polish: The dorsal face shows a dull to greasy marginal polish localized along the very edge. It is only slightly affecting the surface of the rock, its linkage is scattered and the limit between the polished area and the unaffected zone is regular and clear.
The ventral face shows an invasive polish. On the areas where most developed, higher elevations corresponding here to hackles, its topography is domed and its linkage scattered. The polish components are stretched-out forming lines perpendicular to the edge, which might be due either to the motion, either to the morphology of the ventral face (hackles). The limit between the unaffected surface and the polished one is progressive and irregular.

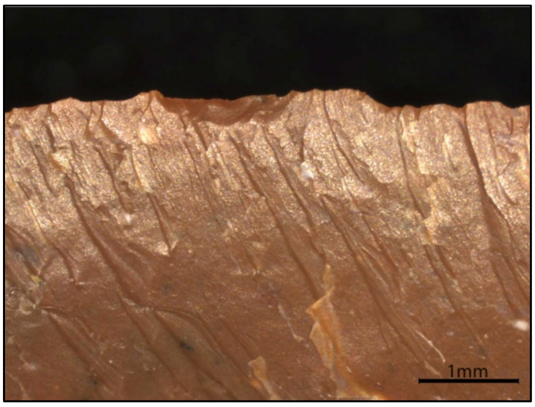

EX12-55A: Micro-scars and gloss on hackles along the edge.

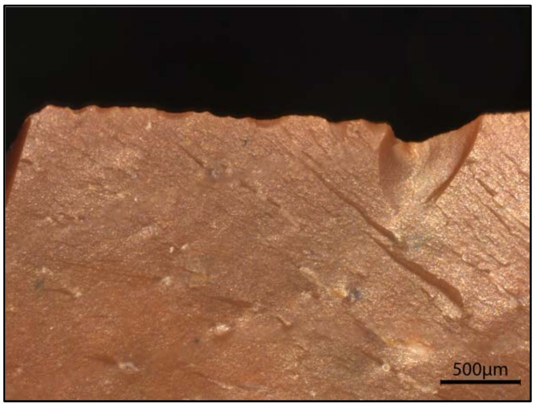

EX12-55B: Small crescent-break micro-scars and one larger break-shallow – step.

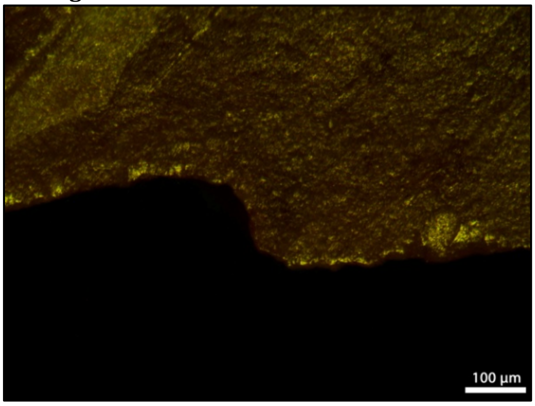

EX12-55C: Polish on the very edge.

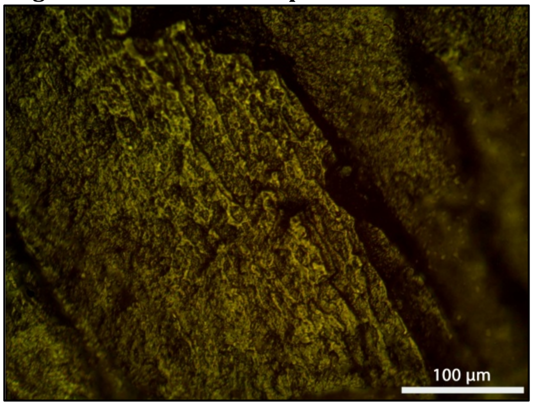

EX12-55D: Domed polish components perpendicular to the edge (due to the motion or to the morphology of the flake – hackles).

Calamus merrillii **-Thinning-** See video EXP-65

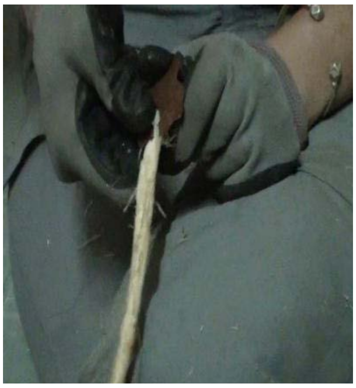

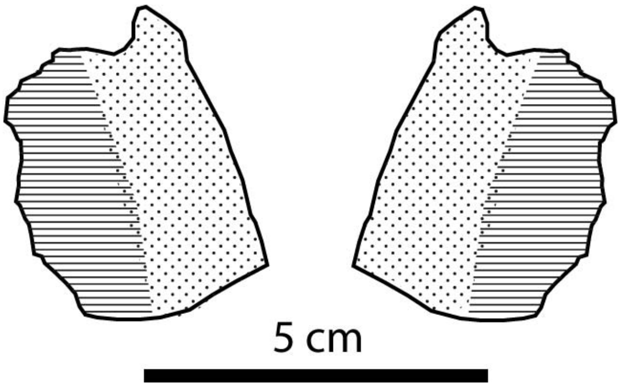

Time: **30 minutes**			Date: **9-1-2012**	Weather: **dry**	
Contact material	Freshness: **fresh**			Degree of moisture: **rather dry**	
	Part of the plant: **split segments of caudex**			Dimension: **width: 1,5cm; thickness: 2cm; length: 15cm**	
	Degree of hardness: **semi-hard**			Degree of penetrability: **medium to penetrate**	
Experimental tool	ID: **EX12-51**	Blank form: **flake**	Type of flake: **long flake**	Cortex: **absent**	Fragmentation: **non fragmented**
	Technological length: **40mm**		Technological width: **41mm**	Maximum length: **40mm**	Maximum width: **33mm**	Maximum thickness: **16mm**
	Location of used area: **left edge**		Type of used area: **unretouched**	Length of used area: **28mm**	Edge angle: **25°**
	Edge profile: **plano-concave**		Edge form: **irregular**	Edge torsion: **minimal**	Number of torsions: -
	Reason for this choice: **The thinness of this flake and low edge angle made it look able to be inserted between two layers of fibers.**				
Motion(s)	Part of the tool handled: **proximal and right parts**			Mode of prehensility: **hands protected with gloves**	
	Type of motion: **thinning down**			Type of percussion: **resting**	
	Contact face: **mostly dorsal. Both faces are in contact when the tool is between fibers.**			Contact angle: **0-59°**	
	Direction of the motion to the active edge: **transversal (+crosswise when making a snick, but marginal)**			Direction of the motion to fibers of the plant: **longitudinal**	
	Way of the motion: **unidirectional (+bidirectional when making a snick, but marginal)**			Tool efficiency to achieve the task: **medium**	
Comments	-				

MICRO-WEAR

EX12-51

Calamus merrillii (fresh)

Thinning

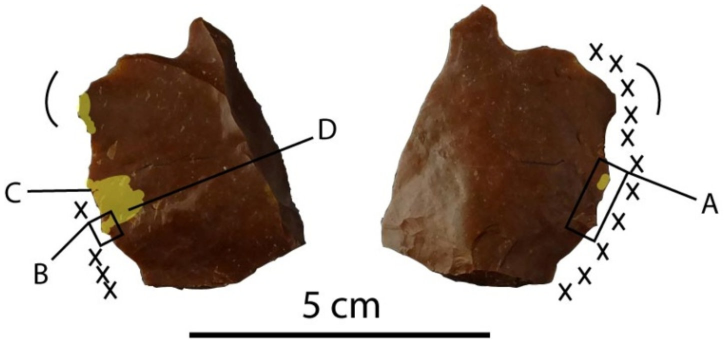

GLOSS: A discreet gloss is visible along the edge under low power microscope.
MICRO-SCARS: A few micro-scars are present predominantly on ventral face. They are distributed in distinct patches, wide in between. Most of them are perpendicular to the edge and half-moon shaped with wide initiation. Their proximal cross-sections are steep and crescent-break and the distal ones are feathered and crescent-break. Some are very large (>2mm in width), others are small (<0,5mm in width) and all are short (0,1-0,3mm in depth).
ROUNDING: A weak discontinuous rounding is visible on both faces. It is rounded with a meplat, the meplat being on the very edge of ventral face.
MICRO-POLISH: Isolated spots of polish are visible on both faces. It is more developed and more invasive on dorsal face where it is metallic and between domed and flat along the terminations of crescent-break micro-scars. Further, it becomes more scatter and is only slightly affecting the surface of the rock. On ventral face, it is dull and undeveloped. The limit between the polished zone and the unaffected surface is progressive and irregular. It appears that there might have been more developed polish along the edge that was removed when micro-chips broke off.

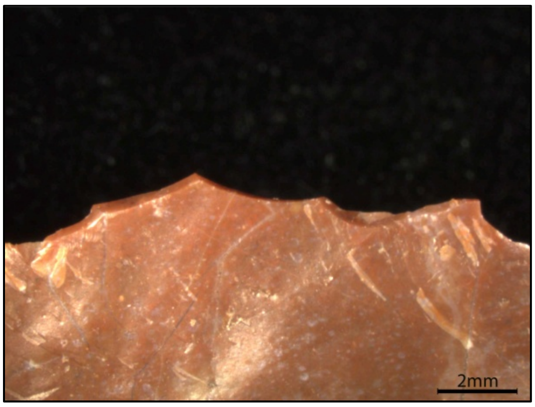

EX12-51A: Crescent-break micro-scars. Gloss on the left micro-chips.

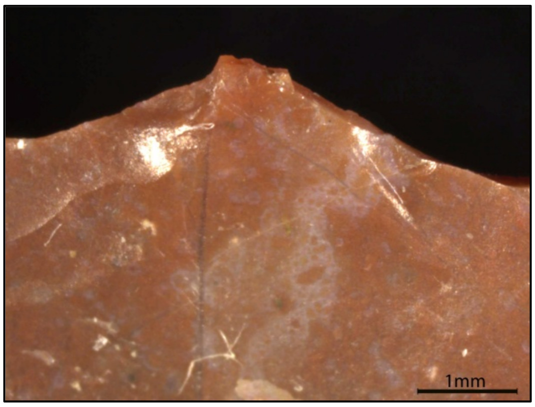

EX12-51B: Gloss along the edge.

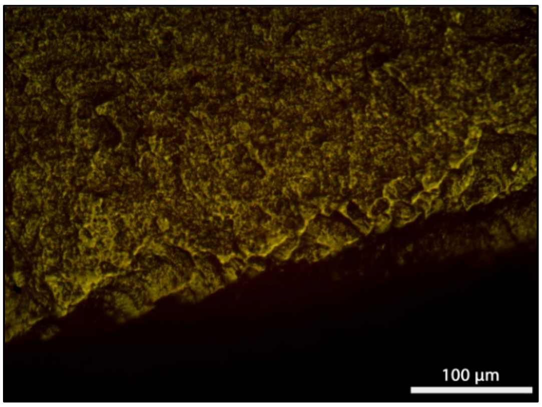

EX12-51C: Polish between domed and flat along the termination of a crescent-break micro-scar.

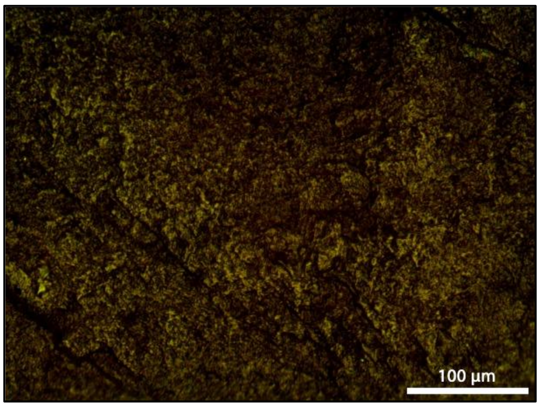

EX12-51D: Polish only slightly affecting the surface affecting the upper parts of the micro-relief.

***Calamus merrillii* - Thinning-** See video EXP-70

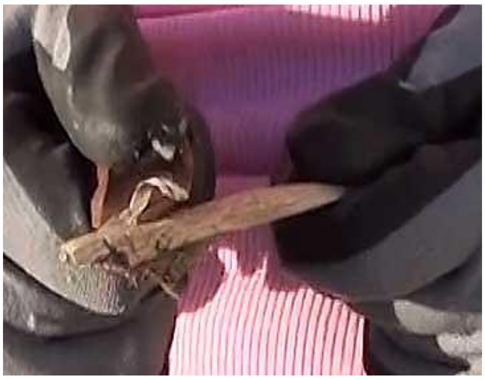

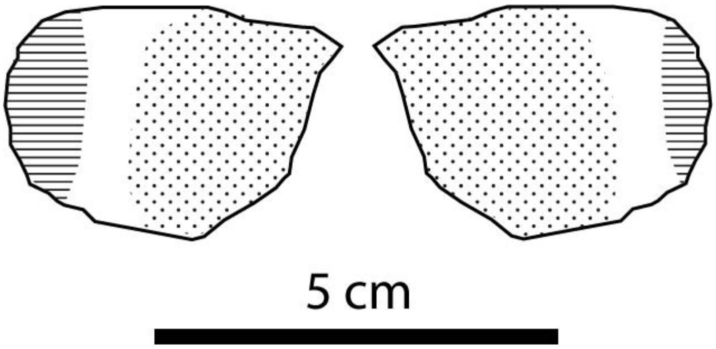

Time: **10 minutes**				Date: **5-12-2013**	Weather: **dry**	
Contact material	Freshness: **dry**			Degree of moisture: **very dry**		
	Part of the plant: **split segment of caudex**			Dimension: **width: 1,5cm; length: 14cm; thickness: 1cm**		
	Degree of hardness: **semi-soft**			Degree of penetrability: **medium to penetrable**		
Experimental tool	ID: **EX12-54**	Blank form: **flake**	Type of flake: **short**	Cortex: **absent**	Fragmentation: **proximal fragment**	
	Technological length: **26mm**		Technological width: **39mm**	Maximum length: **41mm**	Maximum width: **26mm**	Maximum thickness: **10mm**
	Location of used area: **left edge**		Type of used area: **unretouched**	Length of used area: **16mm**	Edge angle: **33°**	
	Edge profile: **biplane**		Edge form: **convex**	Edge torsion: **none**	Number of torsions: -	
	Reason for this choice: **The convex and biplane active edge as well as the flatness of the active part which makes it possible to insert the distal part of the flake between two layers of fibers.**					
Motion(s)	Part of the tool handled: **right part**			Mode of prehensility: **hands protected with gloves**		
	Type of motion: **thinning down**			Type of percussion: **resting**		
	Contact face: **dorsal**			Contact angle: **0-59°**		
	Direction of the motion to the active edge: **transversal**			Direction of the motion to fibers of the plant: **longitudinal**		
	Way of the motion: **unidirectional**			Tool efficiency to achieve the task: **very good**		
Comments	It was way easier to perform this operation on dry rattan than on fresh.					

MICRO-WEAR

EX12-54

Calamus merrillii(dry)

Thinning

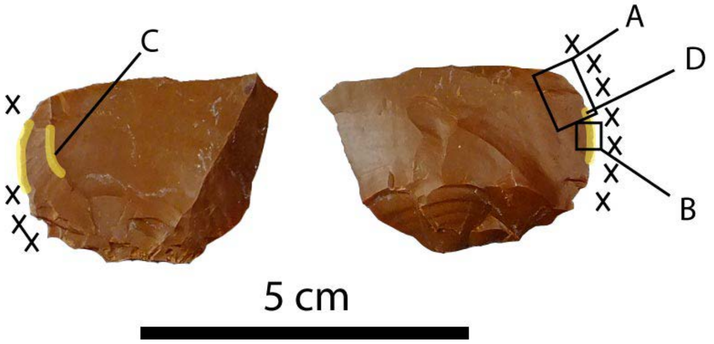

Micro-scars: A medium amount of micro-scars is present, predominantly on ventral face. They are located all along the edge and their distribution is continuous. Their direction to the active edge is perpendicular and their shapes are semi-circular and half-moon with wide initiations. Their proximal cross-sections are crescent-break and steep and the distal ones are crescent-break and feathered. Their width is small (<0,5mm) to medium (0,5-1mm) and their length short (0,1-0,3mm).

Micro-polish: A dull to greasy polish is visible on both faces. It expands in perpendicular from the very edge, being invasive on dorsal face and marginal on ventral. It is only slightly affecting the surface of the rock and its linkage is scarce. The limit between the polished zone and the unaffected one is irregular and progressive.

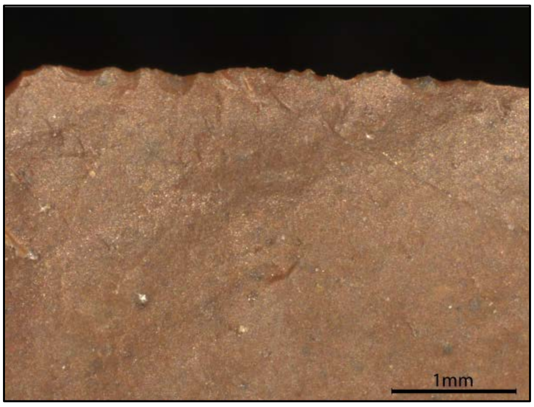

EX12-54A: Small micro-scars along the active edge on ventral face.

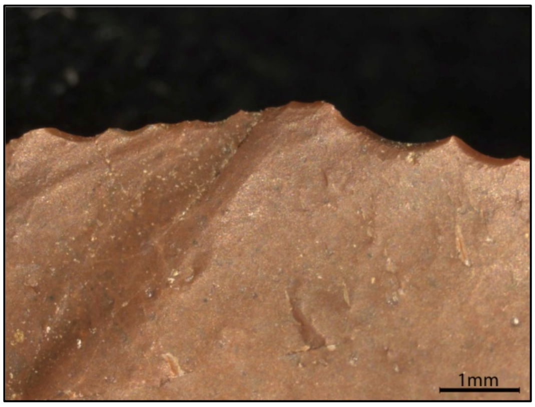

EX12-54B: Half-moon micro-scars.

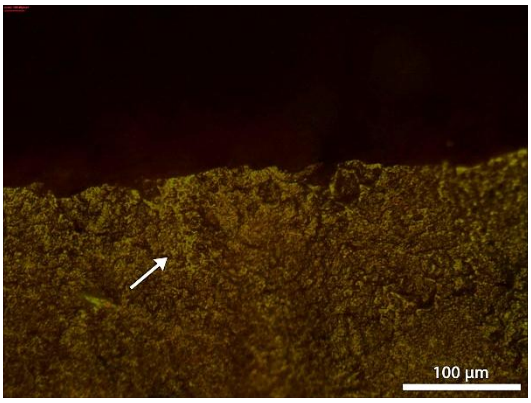

EX12-54C: Dull polish on the very edge and developed perpendicularly to it (see the arrow).

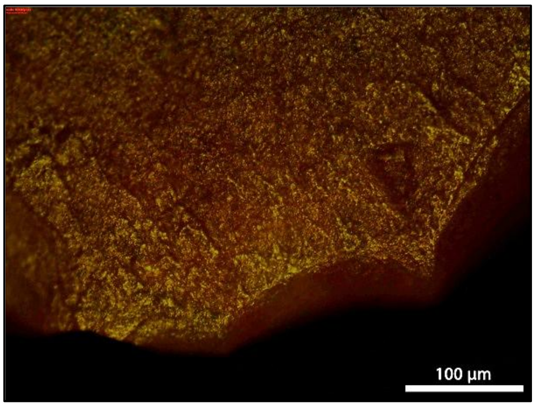

EX12-54D: Micro-polish along the edge and forming lines perpendicular to the edge.

Calamus merrillii - **Thinning**- See video EXP-71

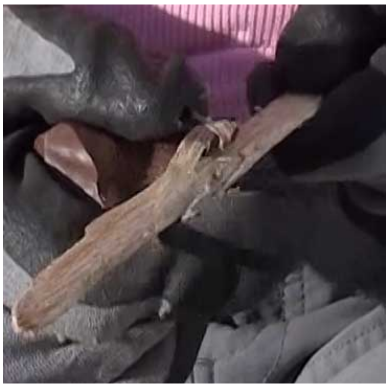

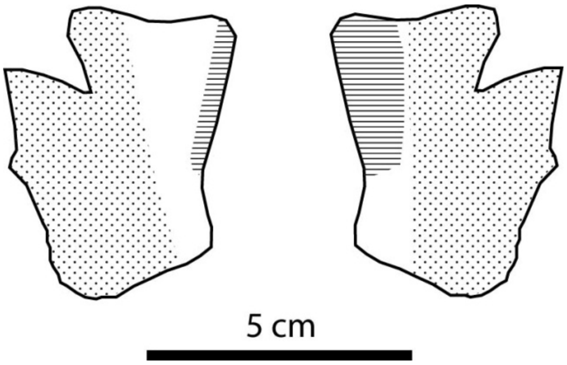

<table>
<tr><td colspan="4">Time: 30 minutes</td><td colspan="2">Date: 5-12-2013</td><td>Weather: dry</td></tr>
<tr><td rowspan="3">Contact material</td><td colspan="3">Freshness: dry</td><td colspan="3">Degree of moisture: very dry</td></tr>
<tr><td colspan="3">Part of the plant: split segment of caudex</td><td colspan="3">Dimension: width: 1,5cm; length: 14cm; thickness: 1cm</td></tr>
<tr><td colspan="3">Degree of hardness: semi-soft</td><td colspan="3">Degree of penetrability: medium to penetrate</td></tr>
<tr><td rowspan="5">Experimental tool</td><td>ID: EX12-56</td><td>Blank form: flake</td><td>Type of flake: long</td><td>Cortex: absent</td><td colspan="2">Fragmentation: non fragmented</td></tr>
<tr><td colspan="2">Technological length: 48mm</td><td>Technological width: 40mm</td><td>Maximum length: 52mm</td><td>Maximum width: 40mm</td><td>Maximum thickness: 16mm</td></tr>
<tr><td colspan="2">Location of used area: distal part of the right edge</td><td>Type of used area: unretouched</td><td>Length of used area: 24mm</td><td colspan="2">Edge angle: 37°</td></tr>
<tr><td colspan="2">Edge profile: biplane</td><td>Edge form: straight</td><td>Edge torsion: none</td><td colspan="2">Number of torsions: -</td></tr>
<tr><td colspan="6">Reason for this choice: The straight biplane and sharp active edge and the large prehensive part, allowing a fluid motion.</td></tr>
<tr><td rowspan="5">Motion(s)</td><td colspan="3">Part of the tool handled: proximal and left parts</td><td colspan="3">Mode of prehensility: hands protected with gloves</td></tr>
<tr><td colspan="3">Type of motion: thinning down</td><td colspan="3">Type of percussion: resting</td></tr>
<tr><td colspan="3">Contact face: ventral</td><td colspan="3">Contact angle: 30-59°</td></tr>
<tr><td colspan="3">Direction of the motion to the active edge: transversal</td><td colspan="3">Direction of the motion to fibers of the plant: longitudinal</td></tr>
<tr><td colspan="3">Way of the motion: unidirectional</td><td colspan="3">Tool efficiency to achieve the task: very good</td></tr>
<tr><td>Comments</td><td colspan="6">It was way easier to perform this operation on dry rattan than on fresh.</td></tr>
</table>

MICRO-WEAR

EX12-56

Calamus merrillii (dry)

Thinning

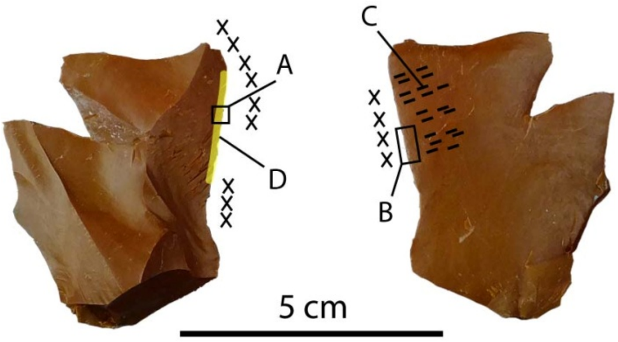

MICRO-SCARS: A medium amount of micro-scars was observed on both faces. They are more numerous and smaller on the dorsal. On the ventral face, they are scarcer but bigger. They are distributed in distinct patches, wide in between and most of them are perpendicular to the edge. Their morphology is semi-circular and quadrangular with wide initiations. Their proximal cross-sections are shallow and steep and the distal ones are feathered. All these micro-chips are small to very small : <0,5mm in width and 0,1-0,3mm in length.

MICRO-POLISH: A smooth and greasy polish was observed on dorsal face along the edge. Its intrusion is medium, its linkage scattered and it is only slightly affecting the topography of the rock, becoming domed at a few places where most developed. The limit between this polished zone and the unaffected surface is irregular and progressive.

Ventral face only presents striation-like polishes – read below.

STRIATIONS: Ventral face shows many brush-stroke striations. They are made of dull striation-like polishes parallel to each other and perpendicular to the edge. They form long and large bright sets indicative of the direction of the motion.

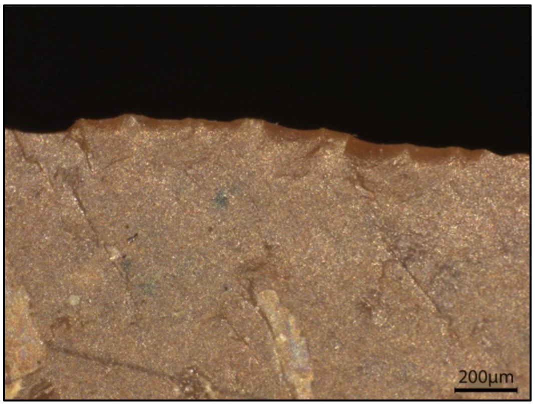

EX12-56A: Small shallow and steep-feathered micro-scars.

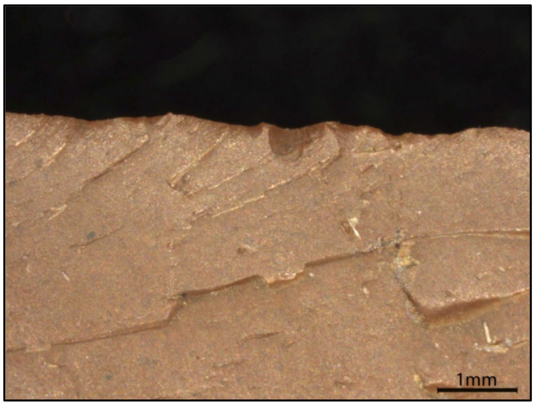

EX12-56B: Small micro-scars.

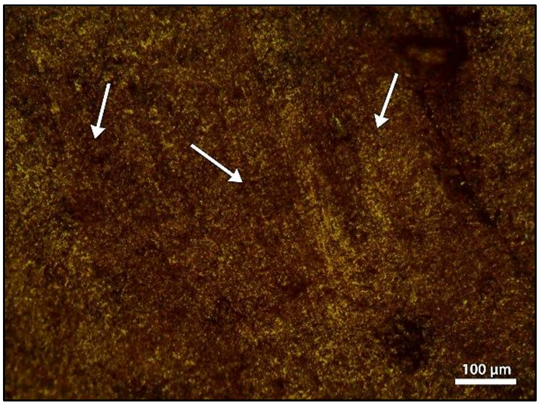

EX12-56C: Dull brush-stroke striations perpendicular to the edge (pointed by arrows).

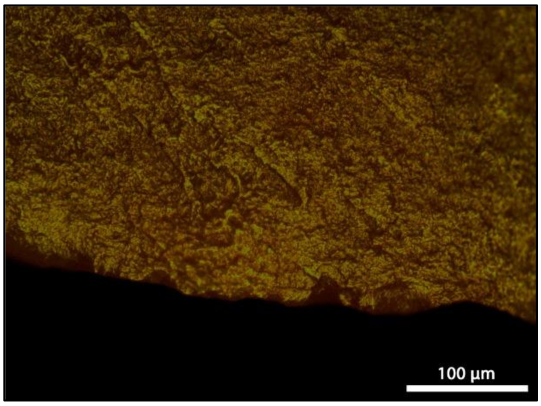

EX12-56D: Greasy polish along the edge.

Arenga pinnata – **Thinning-** See video EXP-82

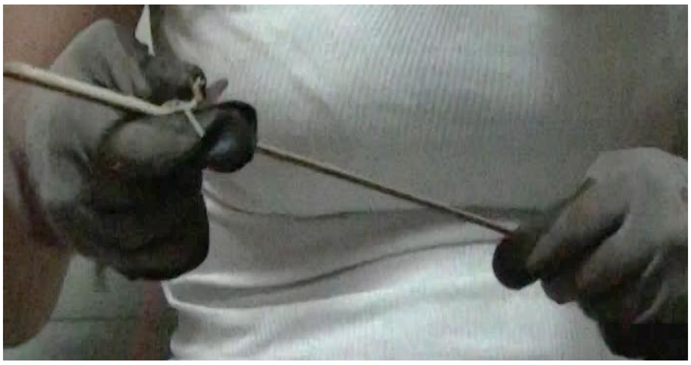

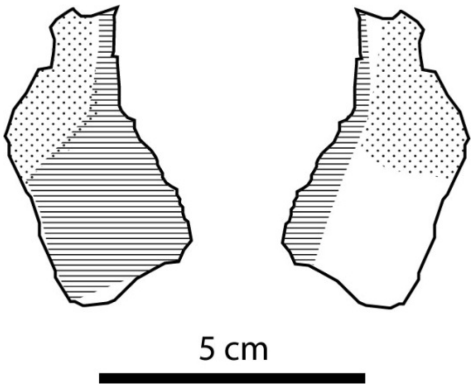

Time: **10 minutes**			Date: **8-31-2012**		Weather: **dry**	
Contact material	Freshness: **fresh (cut 29 hours before)**			Degree of moisture: **rather moist**		
	Part of the plant: **inner part of split leaf petiole segments**			Dimension: length: **length: 70cm; width: 6mm**		
	Degree of hardness: **semi-soft**			Degree of penetrability: **medium to penetrate**		
Experimental tool	ID: **EX12-87**	Blank form: **flake**	Type of flake: **long**	Cortex: **absent**	Fragmentation: **Siret**	
	Technological length: **51mm**		Technological width: **37mm**	Maximum length: **47mm**	Maximum width: **32mm**	Maximum thickness: **7mm**
	Location of used area: **right edge**		Type of used area: **unretouched**	Length of used area: **28mm**	Edge angle: **22°**	
	Edge profile: **biplane**		Edge form: **straight**	Edge torsion: **none**	Number of torsions: -	
	Reason for this choice: **The thinness of this flake and the low edge angle of the active edge make it possible to insert it between two layers of fibers.**					
Motion(s)	Part of the tool handled: **left part**			Mode of prehensility: **hands protected with gloves**		
	Type of motion: **thinning down**			Type of percussion: **resting**		
	Contact face: **dorsal**			Contact angle: **0-59°**		
	Direction of the motion to the active edge: **transversal (+cross-wise when making a snick, but marginal)**			Direction of the motion to fibers of the plant: **longitudinal**		
	Way of the motion: **unidirectional (+bidirectional when making a snick, but marginal)**			Tool efficiency to achieve the task: **good**		
Comments	-					

MICRO-WEAR

EX12-87

Arenga pinnata (fresh)

Thinning

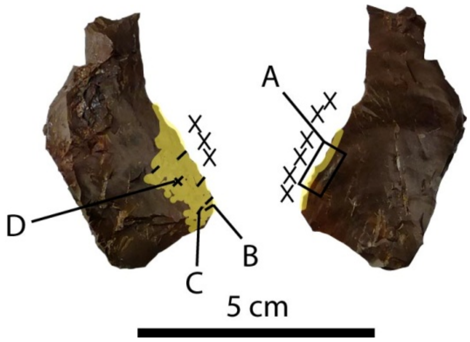

GLOSS: A discreet gloss is visible along the edge under low-power micro-scope.
MICRO-SCARS: A medium amount of micro-scars is present, predominantly on ventral face. Their distribution along the edge is continuous and their direction to the edge perpendicular and transverse bidirectional. Morphologically they are half-moon with wide initiation. Their proximal cross-sections are crescent-break and the distal ones crescent-break and feathered. They are of medium (0,5-1mm) to large (1-2mm) width and short depth (0,1-0,3mm).
MICRO-POLISH: A dull to greasy smooth polish is visible on both faces. It is invasive on dorsal and of medium intrusion on ventral. Its linkage is scattered and its topography only slightly affecting the surface of the rock. The limit between the polished zone and the unaffected area is irregular and progressive.
STRIATIONS: Dorsal face shows numerous brush-stroke striations that are made of striation-like polishes parallel with each other, some of them containing alternating darker narrow grooves. Some present a few domed polish components. They form large sets that are often long but sometimes short (as shown in picture C). They are straight and all orientations to the edge are present: perpendicular, diagonal and parallel, the two first ones being related to thinning down and the last one to snick making.

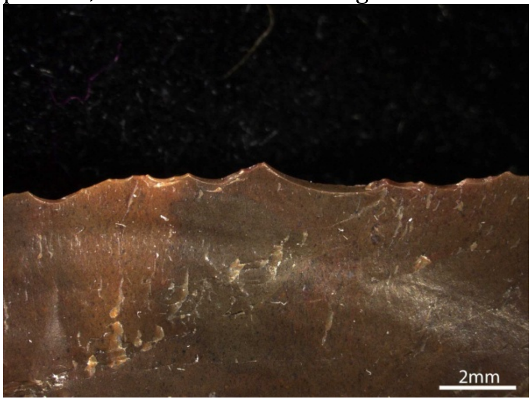

EX12-87A: Small and large crescent-break micro-scars.

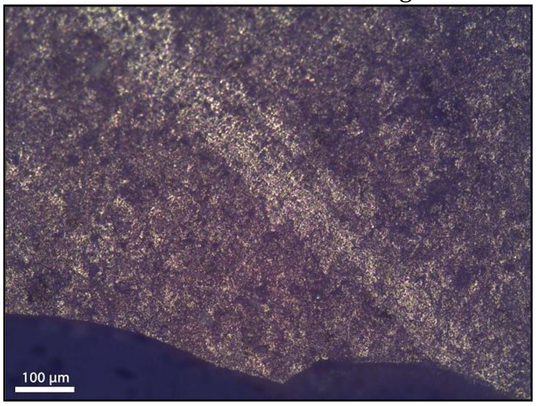

EX12-87B: Long brush-stroke striation diagonal to the edge.

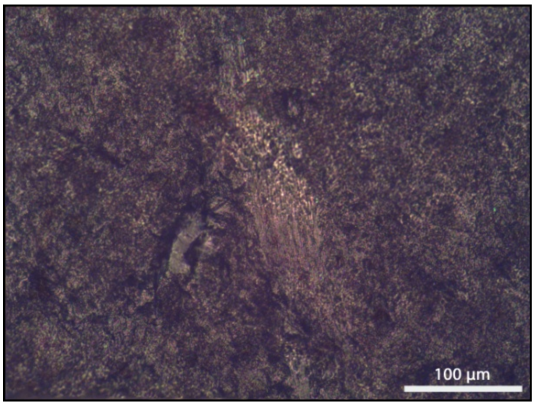

EX12-87C: Brush-stroke striation perpendicular to the edge.

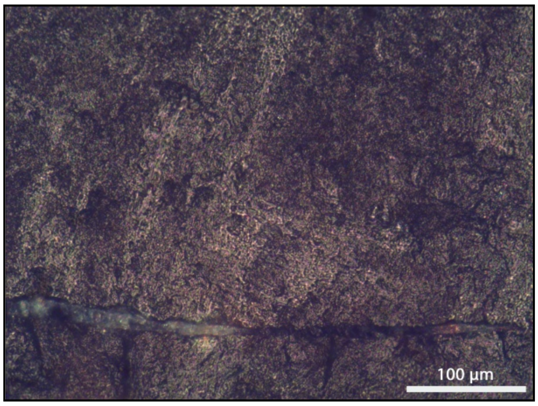

EX12-87D: Brush-stroke striations perpendicular and parallel to the edge, crossing each other. The first are correlated to thinning down, the second to snick making.

Arenga pinnata. – **Thinning-** See video EXP-83

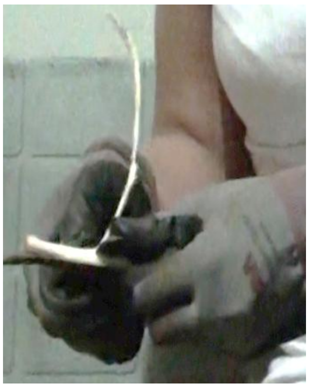

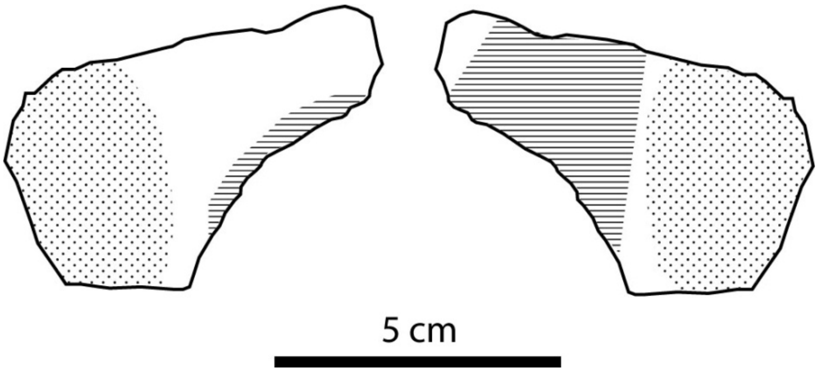

<table>
<tr><td colspan="3">Time: 30 minutes</td><td colspan="2">Date: 8-31-2012</td><td colspan="2">Weather: dry</td></tr>
<tr><td rowspan="3">Contact material</td><td colspan="3">Freshness: fresh (cut 29 hours before)</td><td colspan="3">Degree of moisture: rather moist</td></tr>
<tr><td colspan="3">Part of the plant: inner part of split leaf petiole segments</td><td colspan="3">Dimension: length: length: 70cm; width: 6mm</td></tr>
<tr><td colspan="3">Degree of hardness: semi-soft</td><td colspan="3">Degree of penetrability: medium to penetrate</td></tr>
<tr><td rowspan="5">Experimental tool</td><td>ID: EX12-88</td><td>Blank form: flake</td><td>Type of flake: short</td><td>Cortex: absent</td><td colspan="2">Fragmentation: non fragmented</td></tr>
<tr><td colspan="2">Technological length: 44mm</td><td>Technological width: 63mm</td><td>Maximum length: 64mm</td><td>Maximum width: 44mm</td><td>Maximum thickness: 11mm</td></tr>
<tr><td colspan="2">Location of used area: left edge</td><td>Type of used area: unretouched</td><td>Length of used area: 40mm</td><td colspan="2">Edge angle: 35-45°</td></tr>
<tr><td colspan="2">Edge profile: biplane</td><td>Edge form: concave</td><td>Edge torsion: minimal</td><td colspan="2">Number of torsions: one</td></tr>
<tr><td colspan="6">Reason for this choice: The biplane and concave cutting edge and the large prehensive part.</td></tr>
<tr><td rowspan="5">Motion(s)</td><td colspan="3">Part of the tool handled: right part</td><td colspan="3">Mode of prehensility: hands protected with gloves</td></tr>
<tr><td colspan="3">Type of motion: thinning down</td><td colspan="3">Type of percussion: resting</td></tr>
<tr><td colspan="3">Contact face: ventral</td><td colspan="3">Contact angle: 0-59°</td></tr>
<tr><td colspan="3">Direction of the motion to the active edge: transversal (+cross-wise when making a snick, but marginal)</td><td colspan="3">Direction of the motion to fibers of the plant: longitudinal</td></tr>
<tr><td colspan="3">Way of the motion: unidirectional (+bidirectional when making a snick, but marginal)</td><td colspan="3">Tool efficiency to achieve the task: good</td></tr>
<tr><td>Comments</td><td colspan="6">-</td></tr>
</table>

MICRO-WEAR

EX12-88

Arenga pinnata (fresh)

Thinning

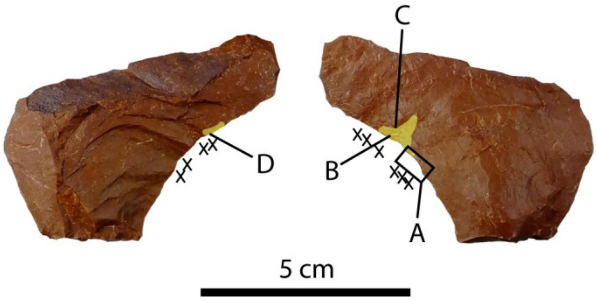

MICRO-SCARS: A medium amount of micro-scars was observed on both faces. They are localized mostly at one very spot, forming a wide notch. This is the part of the edge that was the one mostly in contact with the fibers. The direction of these micro-chips to the edge is perpendicular and transverse bidirectional. Morphologically, they are half-moon and trapezoidal with respectively wide and narrow initiation. Their proximal cross-sections are break-shallow and their distal ones hinge and step. Their width is medium (0,5-1mm) and their depth short for some (0,1-0,3mm) and medium for others (0,7-0,9mm).

MICRO-POLISH: A smooth and metallic polish is present on both faces.

On the ventral, it is localized and invasive, expanding in perpendicular to the edge. On highest elevations it is flat and then becomes domed lower. The polished components are well-linked together and many micro-pits are visible.

On the dorsal, it is distributed in isolated spots and only the highest elevations are polished (scattered linkage), their topography being domed, dotted with a few micro-pits.

On both faces, the limit between the polished area and the unaffected surface is irregular and progressive (lace-like).

STRIATIONS: A few striations perpendicular to the edge are visible on the flat polish components on ventral face. They are narrow, shallow, short and straight and they are slightly darker than the polish.

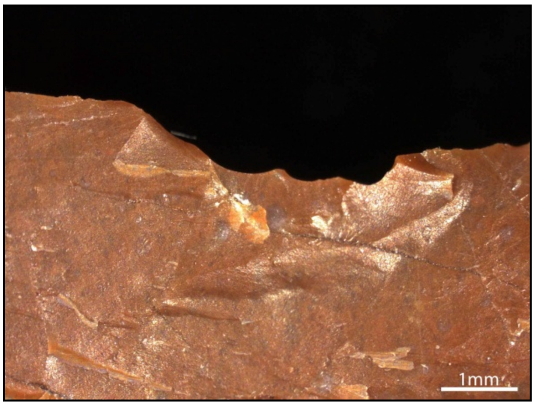

EX12-88A: Several micro-scars forming a large notch on one spot that was very much in contact with the palm fibers.

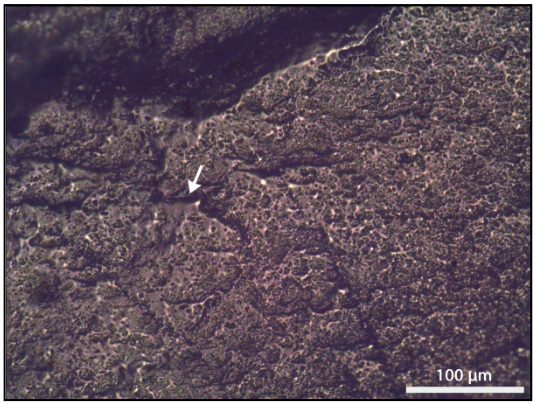

EX12-88B: Polish components that present a flat topography on high elevations with narrow and shallow striations (indicated by the arrow). Domed polish topography further.

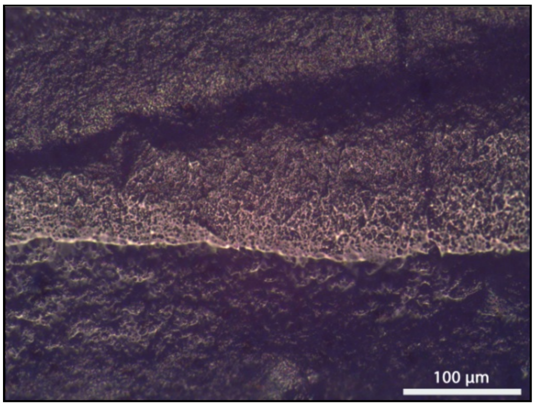

EX12-88C: Flat topography on a ridge. Domed lower, then unaffected surface.

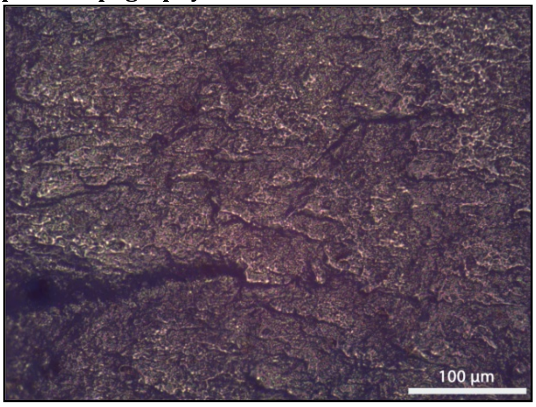

EX12-88D: Domed polish components on highest parts of the relief.

Donax cannaeformis **- Thinning -** See video EXP-98

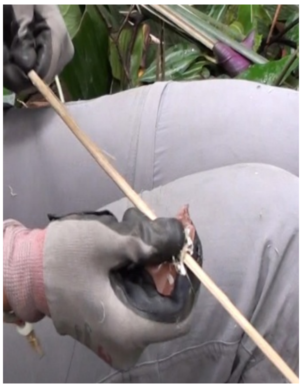

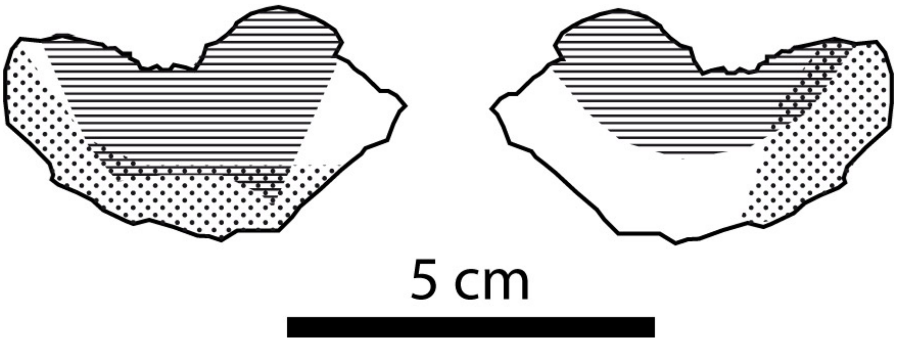

Time: **10 minutes**			Date: **9-2-2012**	Weather: **dry**	
Contact material	Freshness: **fresh**		Degree of moisture: **rather moist**		
	Part of the plant: **inner part of split stem segments**		Dimension: **length: 1m; width: 7mm; thickness: 2-3mm**		
	Degree of hardness: **soft**		Degree of penetrability: **highly penetrable**		
Experimental tool	ID: **EX12-94**	Blank form: **flake**	Type of flake: **short**	Cortex: **absent**	Fragmentation: **distal fragment**
	Technological length: **29mm**	Technological width: **50mm**	Maximum length: **50mm**	Maximum width: **28mm**	Maximum thickness: **15mm**
	Location of used area: **right edge**	Type of used area: **unretouched**	Length of used area: **39mm**	Edge angle: **17-23°**	
	Edge profile: **biplane**	Edge form: **irregular**	Edge torsion: **minimal**	Number of torsions: **one**	
	Reason for this choice: **The low edge angle of the active edge makes it possible to insert it between two layers of fibers.**				
Motion(s)	Part of the tool handled: **left part**		Mode of prehensility: **hands protected with gloves**		
	Type of motion: **thinning down**		Type of percussion: **resting**		
	Contact face: **dorsal**		Contact angle: **0-59°**		
	Direction of the motion to the active edge: **transversal**		Direction of the motion to fibers of the plant: **longitudinal**		
	Way of the motion: **unidirectional**		Tool efficiency to achieve the task: **very good**		
Comments	-				

MICRO-WEAR

EX12-94

Donax cannaeformis

(fresh)

Thinning

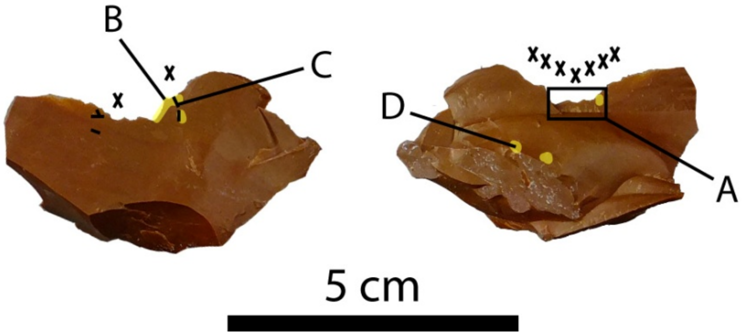

Micro-scars: A medium amount of micro-scars is present predominantly on dorsal face. They are localized and continuous. They are half-moon shaped with wide initiations. Their cross-sections are crescent-break and their width medium (0,5-1mm) to large (1-2mm) while their length is short (0,1-0,3mm). These micro-chips are collected in the concavity of the edge, forming a notch that was useful to wedge the plant fibers to thin them down.

Micro-polish: A smooth but greasy polish is visible on dorsal face. It is distributed in isolated spots and is invasive. Its linkage is scattered and its topography only slightly affecting the surface to slightly domed. Very few micro-pits were observed and the limit between the polished area and the unaffected surface is irregular and progressive (blur). The most developed polish is located along the very edge, on terminations of micro-scars. Elsewhere, it is developed perpendicularly to the edge, in large bands.
On ventral face, only a few polish spots were observed, one of polish expanding perpendicularly to the edge.

Striations: Striation-like polishes diagonal, parallel and perpendicular to the edge were observed. They are straight and of medium length and width.

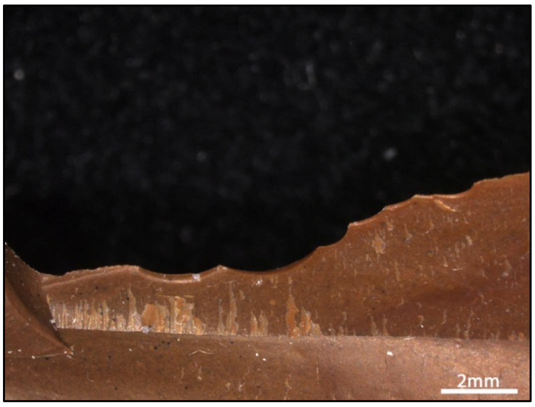

EX12-94A: Crescent-break micro-scars creating a notch.

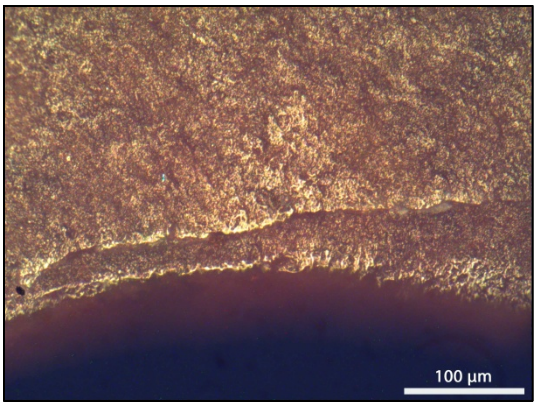

EX12-94B: Polish along the very edge.

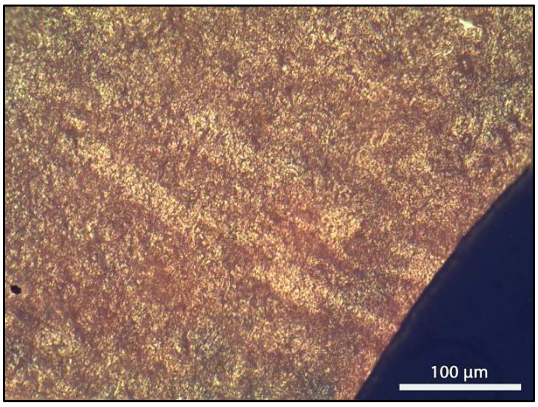

EX12-94C: Bands of polish perpendicular to the edge.

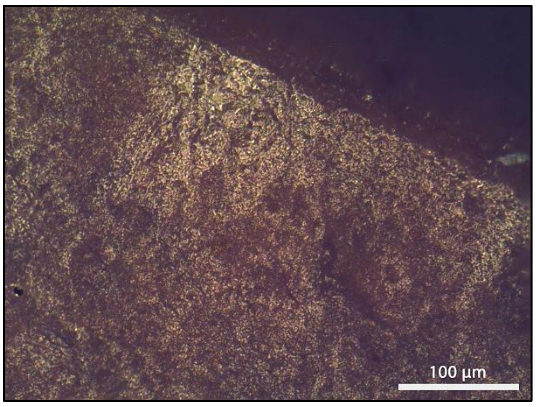

EX12-94D: Polish expanding perpendicularly to the edge.

Donax cannaeformis **- Thinning-** See video EXP-99

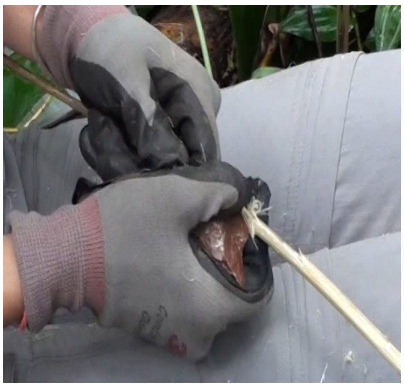

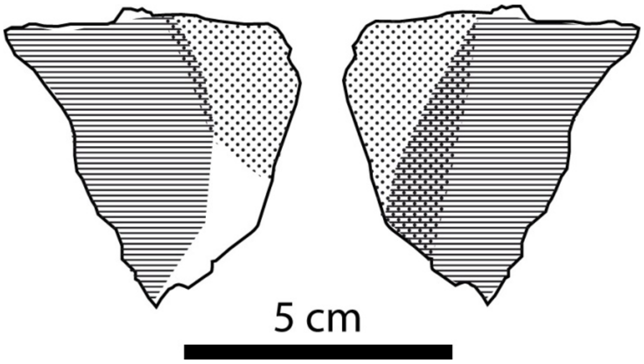

Time: **30 minutes**		Date: **9-2-2012**		Weather: **dry**	
Contact material	Freshness: **fresh**		Degree of moisture: **rather moist**		
	Part of the plant: **inner part of split stem segments**		Dimension: **length: 2m; width: 8mm; thickness: 2-3mm**		
	Degree of hardness: **soft**		Degree of penetrability: **highly penetrable**		
Experimental tool	ID: **EX12-93**	Blank form: **flake**	Type of flake: **short**	Cortex: **<1/3**	Fragmentation: **distal fragment**
	Technological length: **47mm**	Technological width: **48mm**	Maximum length: **49mm**	Maximum width: **47mm**	Maximum thickness: **18mm**
	Location of used area: **left edge**	Type of used area: **unretouched**	Length of used area: **49mm**	Edge angle: **32°**	
	Edge profile: **plano-concave**	Edge form: **irregular**	Edge torsion: **significant**	Number of torsions: **one**	
	Reason for this choice: **The low edge angle of the active edge makes it possible to insert it between two layers of fibers.**				
Motion(s)	Part of the tool handled: **right part**		Mode of prehensility: **hands protected with gloves**		
	Type of motion: **thinning down**		Type of percussion: **resting**		
	Contact face: **ventral**		Contact angle: **0-59°**		
	Direction of the motion to the active edge: **transversal**		Direction of the motion to fibers of the plant: **longitudinal**		
	Way of the motion: **unidirectional**		Tool efficiency to achieve the task: **very good**		
Comments	-				

MICRO-WEAR

EX12-93

Donax cannaeformis (fresh)

Thinning

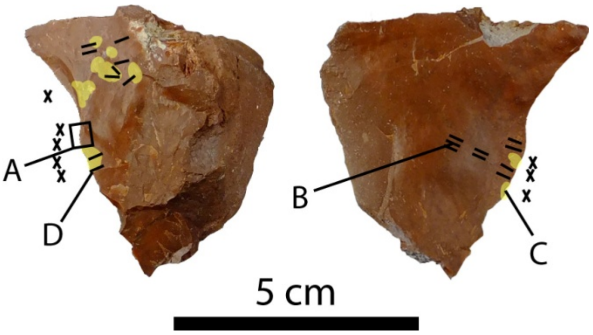

MICRO-SCARS: A few micro-scars are present predominantly on dorsal face. They are localized and mostly perpendicular to the edge. They are trapezoidal and half-moon, with respectively wide and narrow initiations. Their proximal cross-sections are mostly break-shallow and crescent-break and their distal ones step and crescent-break. In size, most are medium (0,5-1m in width) or small (<0,5mm in width) although one is large (1,8mm in width). They are rather short (0,1-0,6mm).

MICRO-POLISH: The very edge shows a very developed smooth and bright polish. It is covering and fluted, the undulations being perpendicular to the edge. It is dotted with a few micro-pits and the limit between the polish and the unaffected surface is progressive.

Ventral face presents a dull to greasy invasive polish. It is scattered and its topography is only slightly affecting the surface to domed. The limit between the polished zone and the unaffected surface is progressive and irregular.

Dorsal face shows a smooth and metallic polish that is scattered to linked. Its topography is mostly domed, although only slightly affecting the surface at some places. Its limit is also progressive and irregular.

STRIATIONS: Both faces show many striation-like polishes that are straight, long and of medium width. They are perpendicular and diagonal to the edge. On dorsal face, dark and deep striations were also observed. They are narrow, straight and perpendicular to the edge.

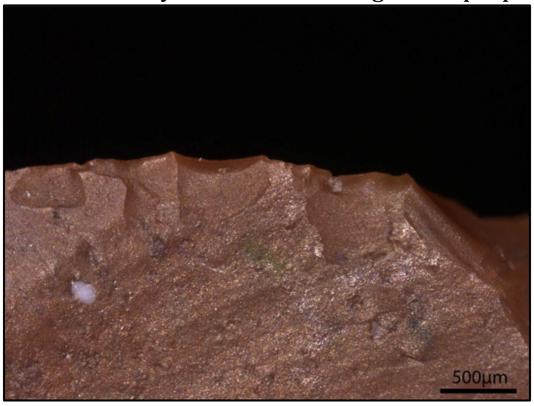

EX12-93A: Small break-shallow initiated micro-scars.

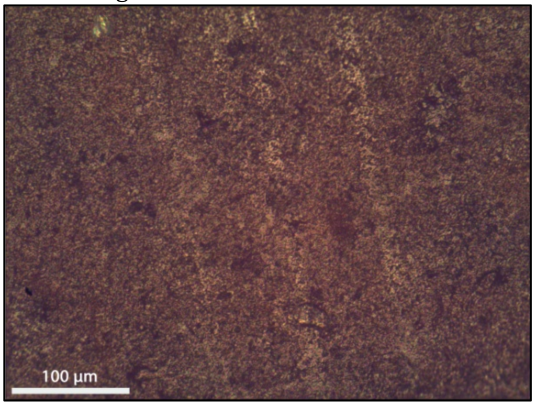

EX12-93B: Striation-like polishes perpendicular to the edge.

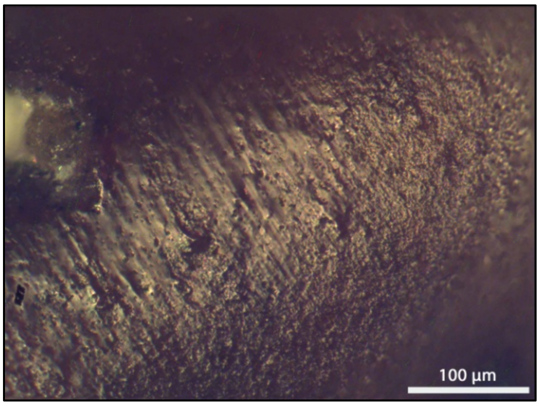

EX12-93C: Fluted polish, the undulations being perpendicular to the edge.

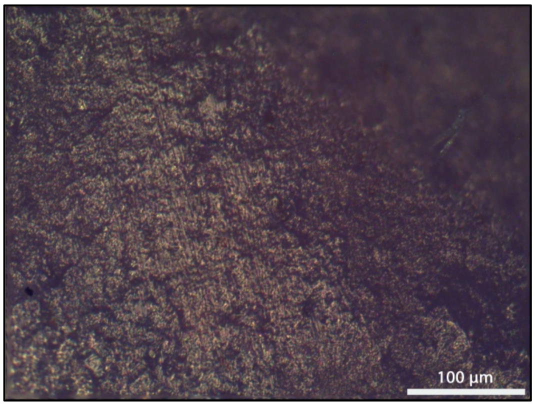

EX12-93D: Deep dark narrow striations perpendicular to the edge.

***Schizostachyum* cf. *lima* – SCRAPING THE EPIDERMIS** - See video EXP-17

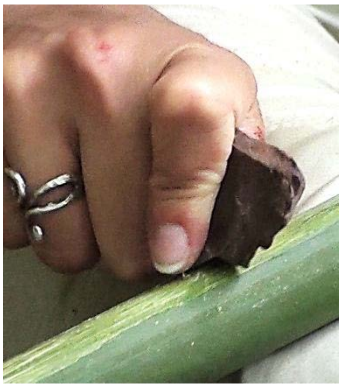

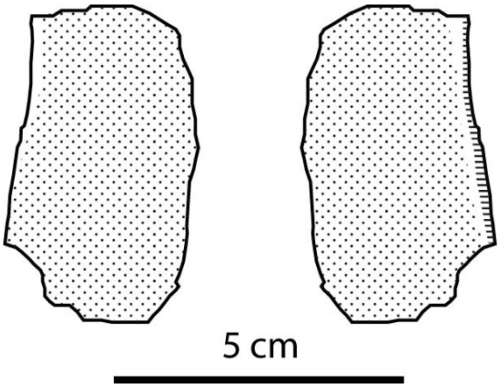

<table>
<tr><td colspan="3">Time: 10 minutes</td><td colspan="2">Date: 8-25-2012</td><td colspan="2">Weather: dry</td></tr>
<tr><td rowspan="3">Contact material</td><td colspan="3">Freshness: fresh</td><td colspan="3">Degree of moisture: rather dry</td></tr>
<tr><td colspan="3">Part of the plant: external part of the culm</td><td colspan="3">Dimension: 3-4 cm of diameter</td></tr>
<tr><td colspan="3">Degree of hardness: hard</td><td colspan="3">Degree of penetrability: -</td></tr>
<tr><td rowspan="5">Experimental tool</td><td>ID: EX12-14</td><td>Blank form: flake</td><td>Type of flake: long flake</td><td>Cortex: absent</td><td colspan="2">Fragmentation: incomplete width</td></tr>
<tr><td colspan="2">Technological length: 49</td><td>Technological width: 35</td><td>Maximum length: 49</td><td>Maximum width: 35</td><td>Maximum thickness: 10.8</td></tr>
<tr><td colspan="2">Location of used area: left edge</td><td>Type of used area: unretouched</td><td>Length of used area: 36,2mm</td><td colspan="2">Edge angle: 82°</td></tr>
<tr><td colspan="2">Edge profile: biplane</td><td>Edge form: straight</td><td>Edge torsion: slight</td><td colspan="2">Number of torsions: one</td></tr>
<tr><td colspan="6">Reason for this choice: Its straight edge with a high angle that makes it unsuitable for cutting but quite efficient for scraping</td></tr>
<tr><td rowspan="5">Motion(s)</td><td colspan="3">Part of the tool handled: right retouched edge</td><td colspan="3">Mode of prehensility: hands</td></tr>
<tr><td colspan="3">Type of motion: scraping</td><td colspan="3">Type of percussion: resting</td></tr>
<tr><td colspan="3">Contact face: ventral</td><td colspan="3">Contact angle: 30°-59°</td></tr>
<tr><td colspan="3">Direction of the motion to the active edge: transversal</td><td colspan="3">Direction of the motion to fibers of the plant: longitudinal</td></tr>
<tr><td colspan="3">Way of the motion: bidirectional</td><td colspan="3">Tool efficiency to achieve the task: medium</td></tr>
<tr><td>Comments</td><td colspan="6">This activity can only be practiced when the bamboo is fresh so there is no equivalent with dry bamboo.</td></tr>
</table>

MICRO-WEAR

EX12-14

Schizostachyum cf. *lima* (fresh)

Scraping the epidermis

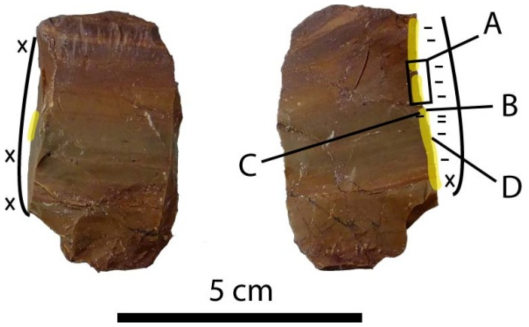

Gloss & Rounding: A strong and glossy rounding, visible to the naked eye, is affecting the very edge, oriented symmetrically between both faces. Its shape is very flat and it is slightly discontinue.

Micro-scars: Only a few micro-scars are present. Three on the dorsal face and one on the ventral. They are perpendicular to the edge, semi-circular and very small (<0,5mm in width, 0,1-0,3mm in depth). Their initiations are wide and shallow and their terminations are hinge and feathered.

Micro-polish: On the very edge (polish bevel), the polish is smooth, very developed and bright. Its topography is flat and at some places domed. Its linkage is covering to linked. Many micro-pits are visible and the limit between this polished bevel and the upper and lower faces is quite clear.

The dorsal face presents a few "tongues" (stretched out components) of very dull polish perpendicular to the rim.

On the ventral face, the polish is smooth, bright and domed along the edge and its linkage and reflexivity gradually diminish as we go further inside the face.

Striations: Numerous narrow, short and shallow dark striations are visible on the very edge (polish bevel), even to the naked eye. They are oriented perpendicularly to the edge.
On the ventral face, there is a medium amount of brush stroke striations made of alternating striation-like polishes and dark deep groves. They are perpendicular and cross-wise to the edge. Their length is medium and they form wide groups.

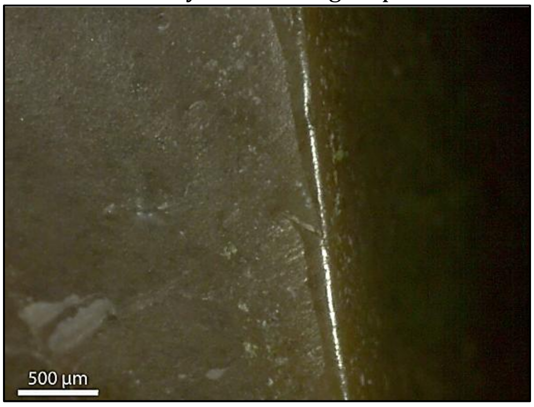

EX12-14A: Flat and glossy rounded edge. Striations perpendicular to it. (Fine dark grooves in the bright line)

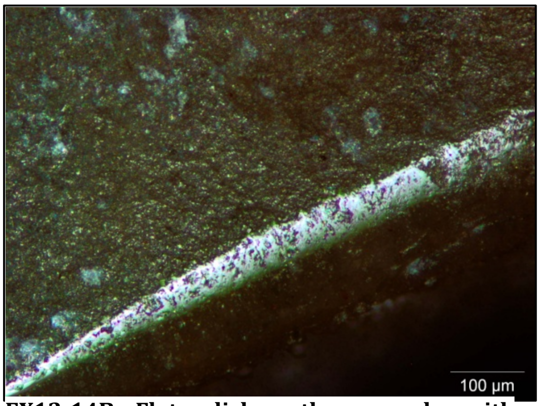

EX12-14B : Flat polish on the very edge with micro-pits and fine dark striations.

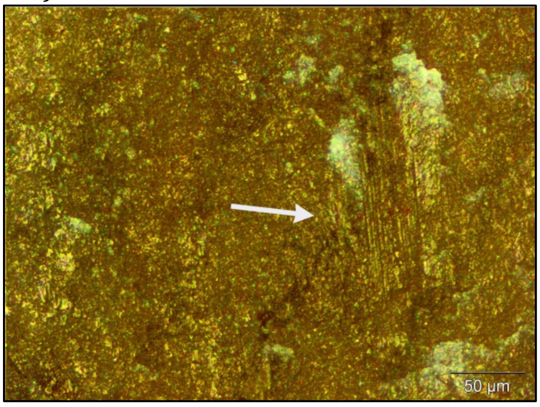

EX12-14C: Brush-stroke striations perpendicular to the edge (indicated by the arrow).

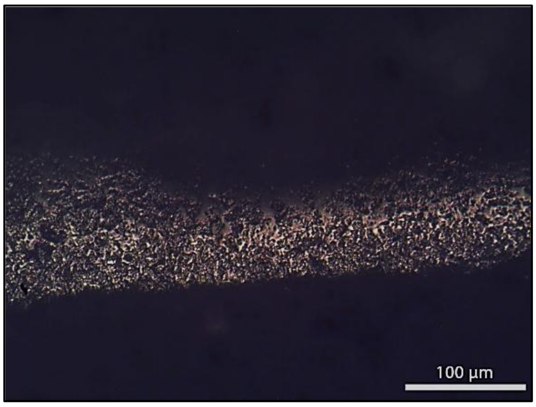

EX12-14D: Flat and domed polish components.

Schizostachyum cf. *lima* - SCRAPING THE EPIDERMIS - See video EXP-18

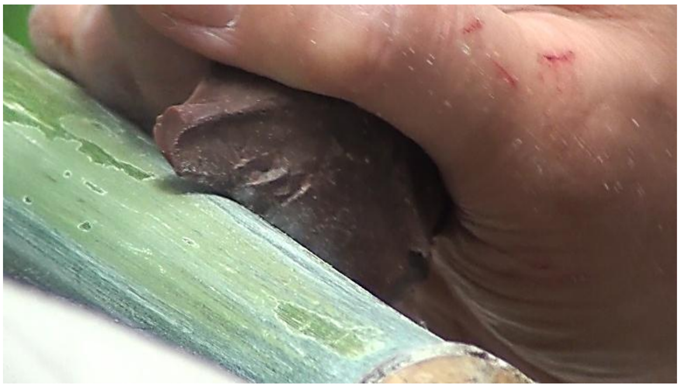

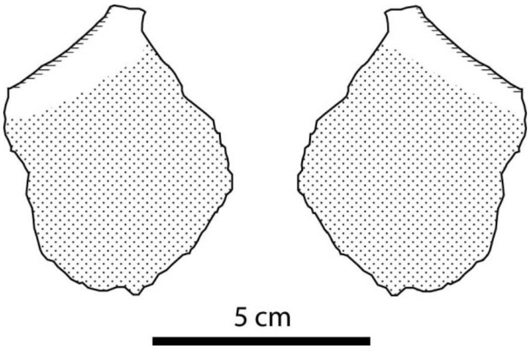

<table>
<tr><td colspan="4">Time: 30 minutes</td><td colspan="2">Date: 8-25-2012</td><td>Weather: dry</td></tr>
<tr><td rowspan="3">Contact material</td><td colspan="3">Freshness: fresh</td><td colspan="3">Degree of moisture: rather dry</td></tr>
<tr><td colspan="3">Part of the plant: external part of the culm</td><td colspan="3">Dimension: 3-4cm of diameter</td></tr>
<tr><td colspan="3">Degree of hardness: hard</td><td colspan="3">Degree of penetrability: -</td></tr>
<tr><td rowspan="5">Experimental tool</td><td>ID: EX12-13</td><td>Blank form: flake</td><td>Type of flake: long flake</td><td>Cortex: absent</td><td colspan="2">Fragmentation: non fragmented</td></tr>
<tr><td colspan="2">Technological length: 60 mm</td><td>Technological width: 46 mm</td><td>Maximum length: 53 mm</td><td>Maximum width: 42 mm</td><td>Maximum thickness: 13,2 mm</td></tr>
<tr><td colspan="2">Location of used area: distal edge</td><td>Type of used area: unretouched</td><td>Length of used area: 27,2mm</td><td colspan="2">Edge angle: 58°</td></tr>
<tr><td colspan="2">Edge profile: Concavo-plan</td><td>Edge form: concave</td><td>Edge torsion: minimal</td><td colspan="2">Number of torsions: one</td></tr>
<tr><td colspan="6">Reason for this choice: the concavity of the edge which makes it compatible with the convexity of the bamboo culm and the morphology of the proximal part that fits well in hands.</td></tr>
<tr><td rowspan="5">Motion(s)</td><td colspan="3">Part of the tool handled: all the tool except the working edge</td><td colspan="3">Mode of prehensility: hands</td></tr>
<tr><td colspan="3">Type of motion: scraping</td><td colspan="3">Type of percussion: resting</td></tr>
<tr><td colspan="3">Contact face: ventral</td><td colspan="3">Contact angle: 30-59°</td></tr>
<tr><td colspan="3">Direction of the motion to the active edge: transversal</td><td colspan="3">Direction of the motion to fibers of the plant: longitudinal</td></tr>
<tr><td colspan="3">Way of the motion: bidirectional</td><td colspan="3">Tool efficiency to achieve the task: medium</td></tr>
<tr><td>Comments</td><td colspan="6">This activity can only be practiced when the bamboo is fresh so there is no equivalent with dry bamboo.</td></tr>
</table>

MICRO-WEAR

EX12-13

Schizostachyum cf. *lima* (fresh)

Scraping the epidermis

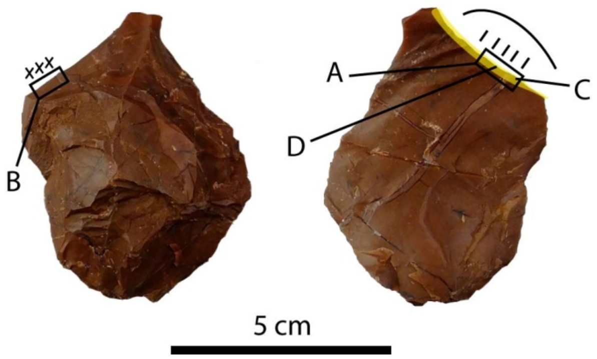

Gloss & Rounding: A strong and glossy continuous rounding, even visible to the naked eye, is affecting the very edge. It is oriented towards the ventral face and its shape is very flat.
Micro-scars: One patch of a few micro-scars occurs on the dorsal face. They are perpendicular to the edge and their shape is semi-circular. Their initiations are steep and their termination mostly feathered. Their size is medium (0,5-1mm in width and 0,4-0,6mm in depth).
Micro-polish: The rounded very edge presents a very bright and developed smooth polish. It is flat and covering, pitted with micro-pits, with a few domed components at some places.
The ventral face shows some greasy to dull polish of medium intrusion oriented perpendicularly to the edge. The dorsal face presents one spot of weakly developed polish.
Striations: The polished and rounded edge shows numerous narrow striations perpendicular to the edge. They are shallow and "filled" with polish (they reflect the light as well and appear as bright). On the ventral face, a few brush-stroke striations are visible, of medium length, perpendicular to the edge.

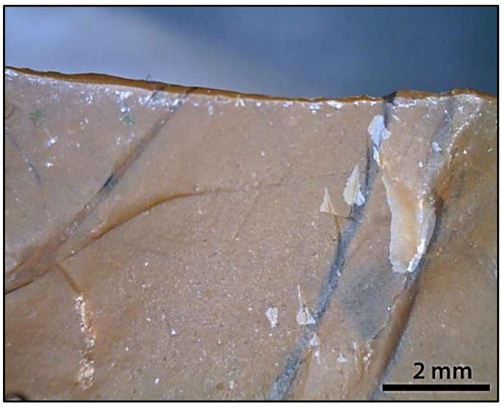

EX12-13A: Flat rounded edge.

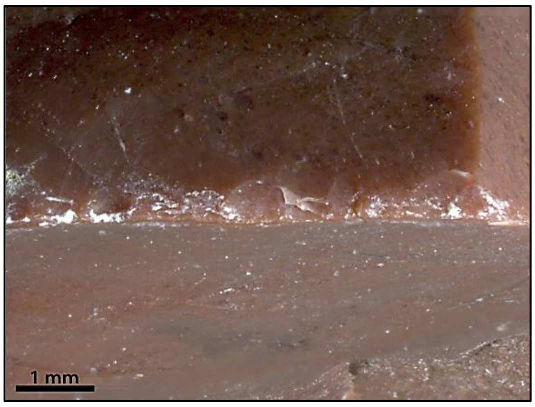

EX12-13B: Micro-scars on the dorsal face, perpendicular to the edge.

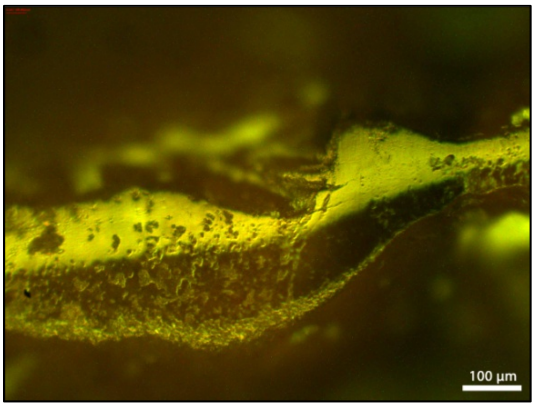

EX12-13C: On the very edge (polish bevel). Very developed flat covering polish with micro-pits and narrow striations perpendicular to the edge.

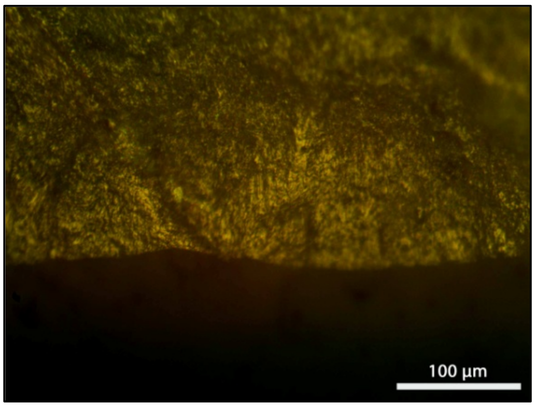

EX12-13D: Ventral face. Greasy polish developed perpendicularly to the edge and striations perpendicular as well.

Dinochloa luconiae – Scraping the epidermis - See video EXP-27

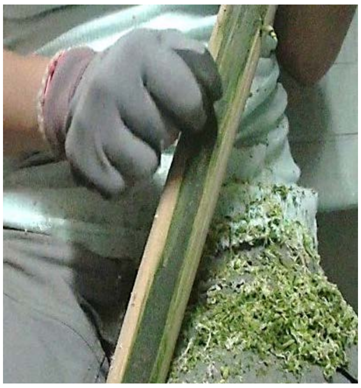

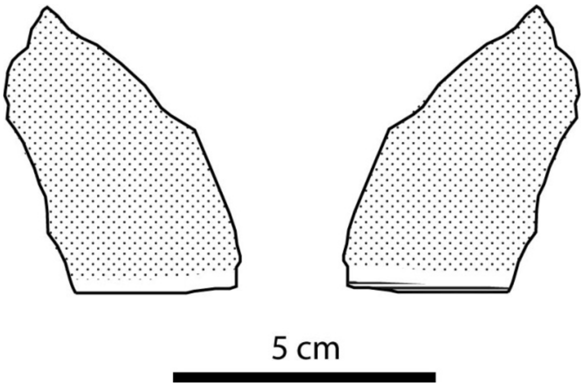

Time: **11 minutes 45 seconds**	Date: **8-26-2012**	Weather: **dry**

Contact material	Freshness: **fresh**		Degree of moisture: **rather dry**		
	Part of the plant: **external part of the stem**		Dimension: **L=45cm, diameter=3,5cm**		
	Degree of hardness: **hard**		Degree of penetrability: **difficult to penetrate** – easy to scrape off		
Experimental tool	ID: **EX12-67**	Blank form: **flake**	Type of flake: **short flake**	Cortex: **absent**	Fragmentation: **multiple fractures**[1]
	Technological length: **34mm**	Technological width: **57mm**	Maximum length: **49mm**	Maximum width: **35mm**	Maximum thickness: **11mm**
	Location of used area: **proximal edge**	Type of used area: **unretouched**	Length of used area: **29 mm**	Edge angle: **88°**	
	Edge profile: **biplane**	Edge form: **irregular**	Edge torsion: **minimal**	Number of torsions: **one**	
	Reason for this choice: **the fact that the edge is relatively straight and has a high angle**				
Motion(s)	Part of the tool handled: **all except the active edge**		Mode of prehensility: **hands protected with gloves**		
	Type of motion: **scraping**		Type of percussion: **resting**		
	Contact face: **ventral**		Contact angle: **30-59°**		
	Direction of the motion to the active edge: **transversal**		Direction of the motion to fibres of the plant: **longitudinal**		
	Way of the motion: **bidirectional**		Tool efficiency to achieve the task: **very good**		
Comments	Although *Dinochloa luconiae* is a very hard material, the external green part of the stem was surprisingly easy to remove. It was easier to perform this operation with this contact material than with *Schizostachyum sp.*				

[1] As this flake is very fragmented it is oriented according to its morphological axis.

MICRO-WEAR

EX12-67
Dinochloa luconiae (fresh)
Scraping the epidermis

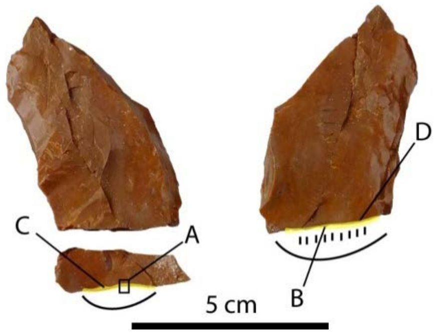

GLOSS & ROUNDING: A strong and glossy continuous rounding, even visible to the naked eye, is affecting the very edge. It is slightly oriented towards the ventral face and its shape is very flat.
MICRO-SCARS: No micro-scars were observed on this tool.
MICRO-POLISH: On the very edge (polish bevel), the polish is smooth, very developed and bright. Its topography is flat, its linkage is covering and it is dotted with many micro-pits.
This micro-polish is expanding on the dorsal face, its intrusion being medium on the meplat between the two faces. It is progressively less developed as further from the active ridge, the transition between the polished and the unaffected zone being progressive and irregular. Along the edge, its characteristics are the same than on the bevel. Further, its topography gradually becomes domed and its linkage less and less linked.
The polish is intruding marginally on the ventral face at one specific place. It is flat and covering along the very edge, then becomes domed and linked as further from the rim.
This repartition of the polish can be correlated with a high working angle.
STRIATIONS: Many striations are visible on the very rim (polish bevel). They are very narrow, shallow, short and dark. They are perpendicular to the edge.

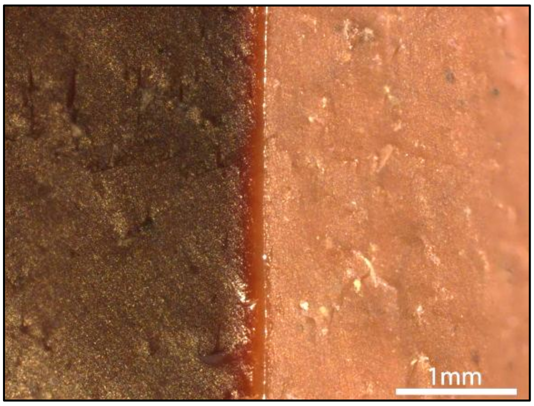

EX12-67A: Glossy flat shaped rounding on the very edge.

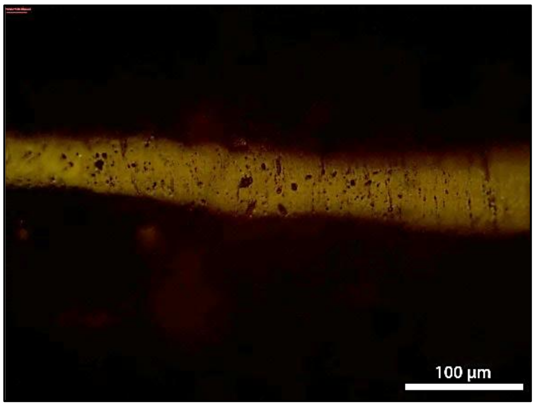

EX12-67B: Flat polish dotted with micro-pits and narrow shallow striations perpendicular to the edge.

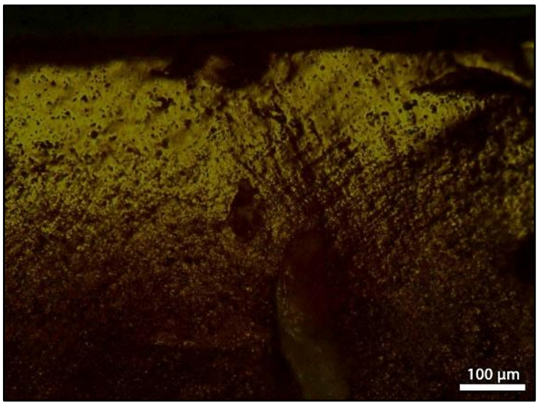

EX12-67C: Very developed polish on the meplat between ventral and dorsal face, expanding from the flat rounding (polish bevel) of the edge.

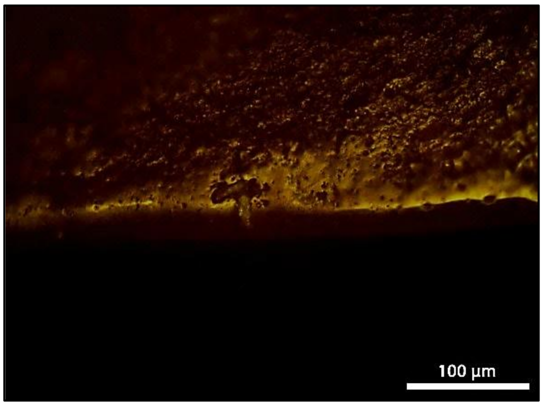

EX12-67D: Marginal polish on the ventral face, expanding from the flat rounding (polish bevel) of the active edge.

Dinochloa luconiae – Scraping the epidermis - See video EXP-28

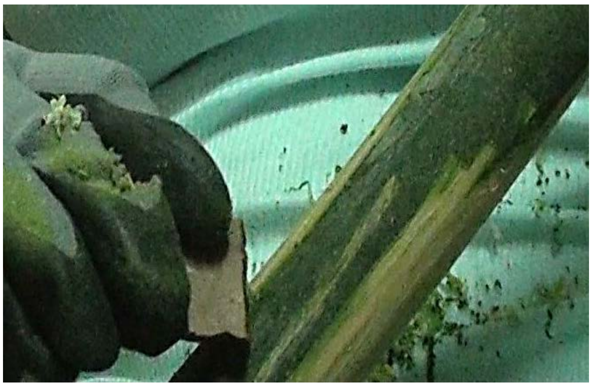

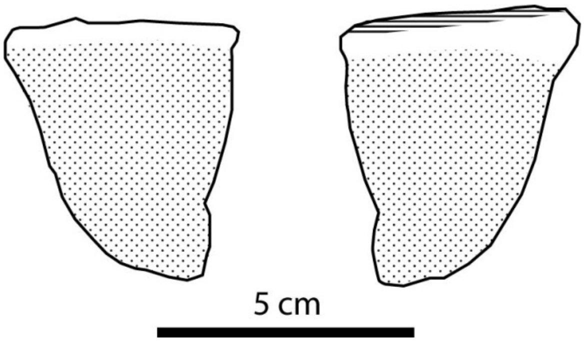

Time: **30min**			Date: **8-26-2012**		Weather: **dry**
Contact material	Freshness: **fresh**		Degree of moisture: **rather dry**		
	Part of the plant: **external part of the stem**		Dimension: **L=45cm, diameter=3,5cm**		
	Degree of hardness: **hard**		Degree of penetrability: **difficult to penetrate** – easy to scrape off		
Experimental tool	ID: **EX12-66**	Blank form: **flake**	Type of flake: **long flake**	Cortex: **<1/3**	Fragmentation: **incomplete width**
	Technological length: **48mm**	Technological width: **42mm**	Maximum length: **46mm**	Maximum width: **42mm**	Maximum thickness: **12mm**
	Location of used area: **distal edge**	Type of used area: **unretouched**	Length of used area: **39mm**	Edge angle: **81°**	
	Edge profile: **biplane**	Edge form: **straight**	Edge torsion: **slight**	Number of torsions: **one**	
	Reason for this choice: **Its open angled and straight edge which makes it able to scrape without scratching or cutting the bamboo.**				
Motion(s)	Part of the tool handled: **proximal**		Mode of prehensility: **hands protected with gloves**		
	Type of motion: **scraping**		Type of percussion: **resting**		
	Contact face: **ventral**		Contact angle: **30-59°**		
	Direction of the motion to the active edge: **transversal**		Direction of the motion to fibres of the plant: **longitudinal**		
	Way of the motion: **bidirectional**		Tool efficiency to achieve the task: **very good**		
Comments	Although *Dinochloa luconiae* is a very hard material, the external green part of the stem was surprisingly easy to remove. It was easier to perform this operation with this contact material than with *Schizostachyum sp.*				

MICRO-WEAR

EX12-66
Dinochloa luconiae (fresh)
Scraping the epidermis

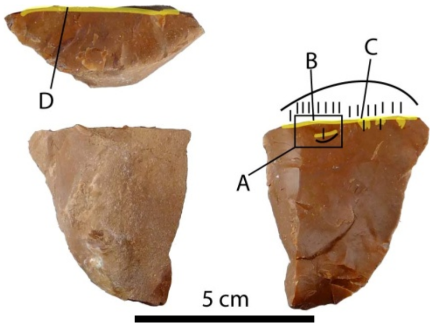

GLOSS & ROUNDING: A strong and glossy continuous rounding, even visible to the naked eye, is affecting the very edge. It is oriented towards the ventral face and its shape is very flat. On ventral face, a protuberance due to knapping (an undulation located 4mm from the edge) is also rounded and glossy.
MICRO-SCARS: No micro-scars were observed on this tool.
MICRO-POLISH: The very edge shows an extremely developed smooth polish. It is bright with a covering linkage and a flat topography, although some spots exhibit a fluted polish, its undulations being parallel to the edge. It is dotted with micro-pits.
This polish expands on both faces, perpendicularly to the edge, being more invasive on the ventral (medium intrusion against marginal on the dorsal). Along the edge it is flat, covering and dotted with micro-pits, then, it becomes progressively less developed as further away from the rim. In these areas of less intensive contact, the polish is domed and the linkage linked then scattered. The polish components are stretched out, forming lines perpendicular to the edge at some places. The transition between the polished and the unaffected zone is progressive and irregular.
The rounded and glossy protuberance of the ventral face shows a similar polish: flat, covering and dotted with micro-pits at its highest elevation and domed with a linked to scattered linkage at lower ones.
STRIATIONS: The ventral face and the rounded active ridge show numerous striations perpendicular to the edge. They are extremely narrow, shallow, straight and short and their color is bright, as the polish.

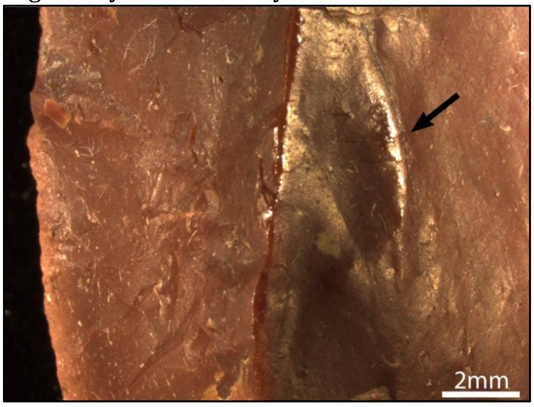

EX12-66A: The active edge is rounded and glossy, as well as a protuberance on the ventral face (arrow).

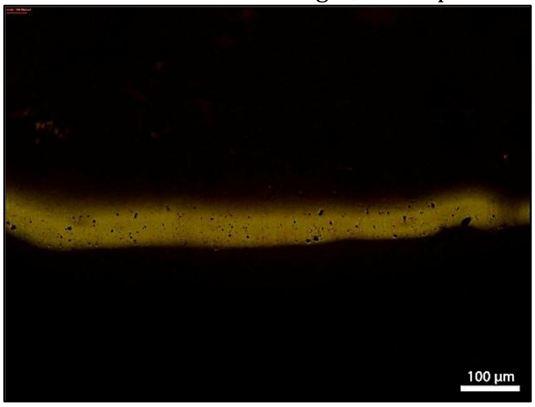

EX12-66B: Flat polish dotted with micro-pits and shallow and narrow striations on the very edge.

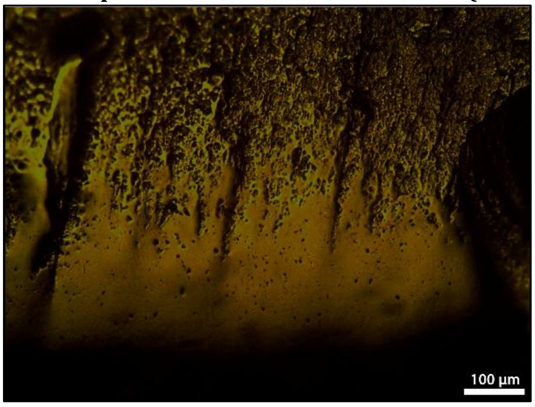

EX12-66C: Flat covering polish along the edge and stretched out domed components further inside.

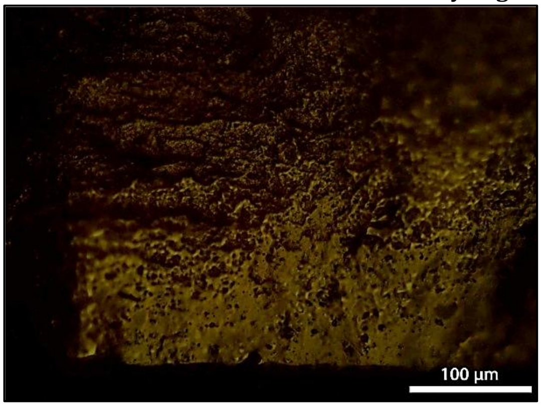

EX12-66D: On the meplat between ventral and dorsal face, a rather marginal polish expansion.

Arenga pinnata- Scraping the epidermis- See video EXP-78

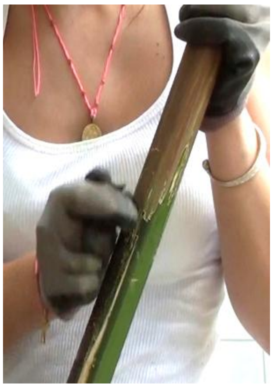

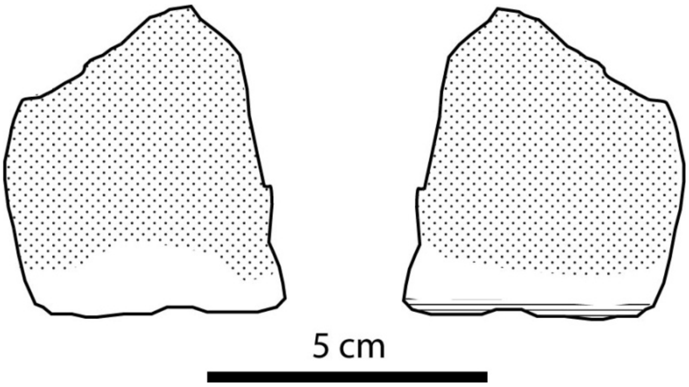

Time: **10 minutes**	Date: **8-31-2012**	Weather: **dry**

Contact material	Freshness: **fresh (cut 18h before)**		Degree of moisture: **rather dry**		
	Part of the plant: **outer part of leaf petiole**		Dimension: **length: 1,5-1,6m long; diameter: 3,5cm**		
	Degree of hardness: **semi-soft**		Degree of penetrability: **highly penetrable**		
Experimental tool	ID: **EX12-105**	Blank form: **core**	Type of flake: -	Cortex: **absent**	Fragmentation: **non fragmented**
	Technological length: **-**		Technological width: -	Maximum length: **49mm**	Maximum width: **43mm** / Maximum thickness: **25mm**
	Location of used area: **proximal edge**		Type of used area: **unretouched**	Length of used area: **38mm**	Edge angle: **60-69°**
	Edge profile: **biconcave**		Edge form: **irregular**	Edge torsion: **significant**	Number of torsions: **several**
	Reason for this choice: **The high angle of the active edge that makes it able to scrape without cutting.**				
Motion(s)	Part of the tool handled: **distal**		Mode of prehensility: **hands protected with gloves**		
	Type of motion: **scraping**		Type of percussion: **dynamic**		
	Contact face: **ventral**		Contact angle: **60-89°**		
	Direction of the motion to the active edge: **transversal**		Direction of the motion to fibers of the plant: **longitudinal**		
	Way of the motion: **bidirectional**		Tool efficiency to achieve the task: **very good**		
Comments	-				

MICRO-WEAR

EX12-105

Arenga pinnata (fresh)

Scraping the epidermis

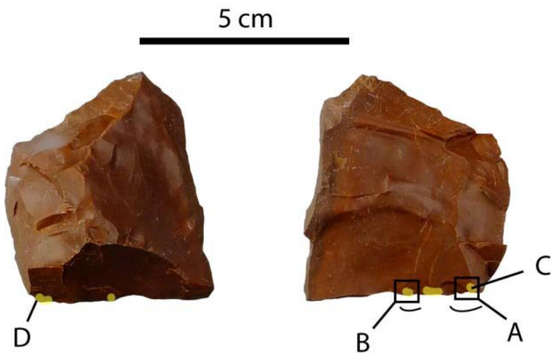

Gloss: Interrupted gloss is visible along the edge even to the naked eye.

Micro-scars: The only ones were already present before use and correspond to very small removals.

Rounding: A flat rounding is visible on ventral face. It is of medium intensity and discontinuous, being located on prominent parts.

Micro-polish: Ventral face shows a smooth and bright polish distributed in large isolated spots along the edge. This is due to the fact that the latter is very irregular so only its most prominent parts were in contact with the leaf petiole. This polish is of medium intrusion and along the edge, it is covering and flat, being dotted with micro-pits. Further, it turns slightly domed, its linkage becoming more scattered. The limit between the polished zone and the unaffected one is progressive (blur).
Dorsal face shows a few spots of greasy polish. It is marginal, its linkage is scattered and its topography domed. The limit between the unaffected surface and the polished one is irregular and progressive.

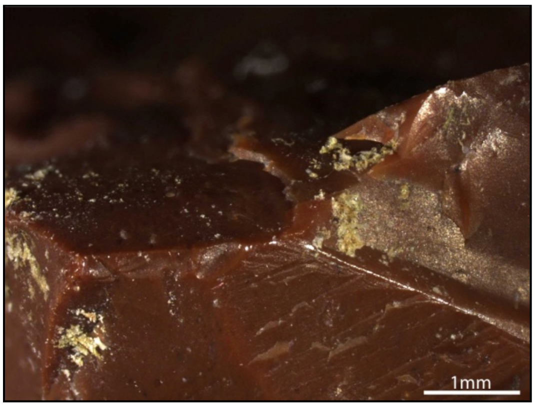

EX12-105A: Glossy rounding on ridges and prominent parts.

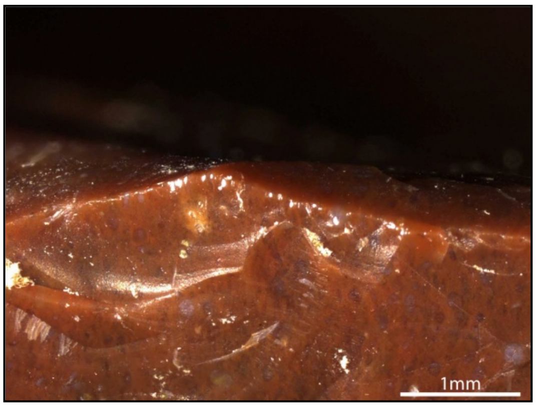

EX12-105B: Glossy rounding on the very edge.

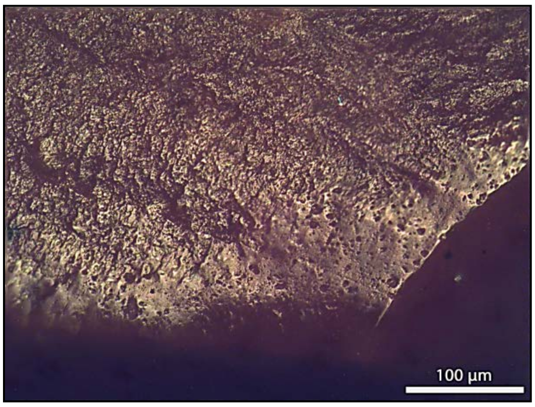

EX12-105C: Flat covering polish along the edge, dotted with micro-pits.

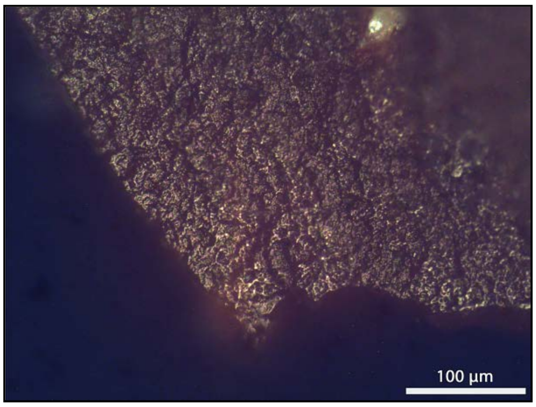

EX12-105D: Greasy and marginal domed polish

Arenga pinnata – Scraping the epidermis- See video EXP-79

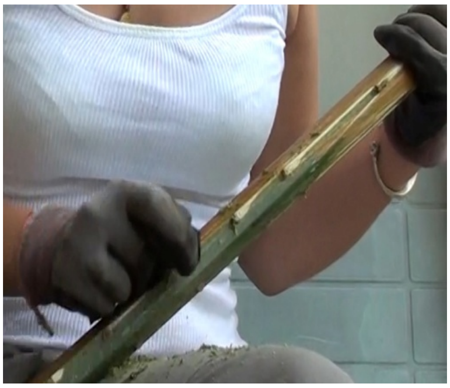

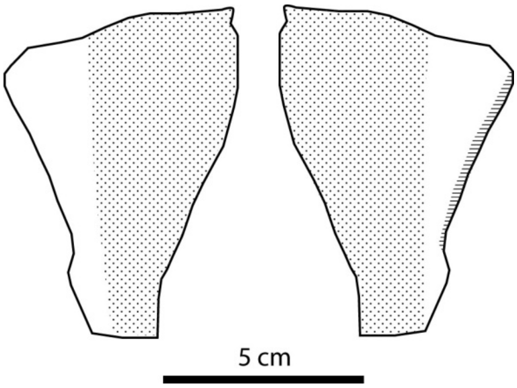

Time: **30 minutes**			Date: **8-31-2012**	Weather: **dry**	
Contact material	Freshness: **fresh (cut 18h before)**		Degree of moisture: **rather dry**		
	Part of the plant: **outer part of leaf petiole**		Dimension: **length: 1,5-1,6m long; diameter: 3,5cm**		
	Degree of hardness: **semi-soft**		Degree of penetrability: **highly penetrable**		
Experimental tool	ID: **EX12-106**	Blank form: **flake**	Type of flake: **long**	Cortex: **absent**	Fragmentation: **non fragmented**
	Technological length: **70mm**	Technological width: **54mm**	Maximum length: **70mm**	Maximum width: **54mm**	Maximum thickness: **21mm**
	Location of used area: **left edge on ventral face**	Type of used area: **unretouched**	Length of used area: **39mm**	Edge angle: **75°**	
	Edge profile: **biplane**	Edge form: **straight**	Edge torsion: **minimal**	Number of torsions: **one**	
	Reason for this choice: **The high angle of the active edge and its straight form that make it able to scrape without cutting.**				
Motion(s)	Part of the tool handled: **right part**		Mode of prehensility: **hands protected with gloves**		
	Type of motion: **scarping**		Type of percussion: **resting**		
	Contact face: **ventral**		Contact angle: **60-89°**		
	Direction of the motion to the active edge: **transversal**		Direction of the motion to fibers of the plant: **longitudinal**		
	Way of the motion: **bidirectional**		Tool efficiency to achieve the task: **very good**		
Comments	-				

MICRO-WEAR

EX12-106

Arenga pinnata (fresh)

Scraping the epidermis

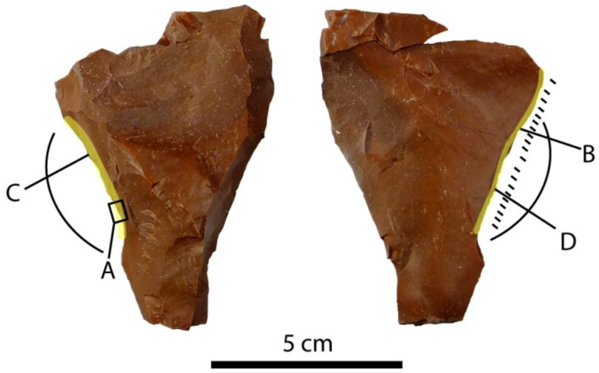

Gloss & Rounding: A flat glossy rounding is visible on the very edge even to the naked eye. Its intensity is strong and it is continuous.

Micro-polish: On the very edge, a very bright and smooth polish is present. It is covering, flat and dotted with micro-pits. It expands on both faces.

On dorsal, its intrusion is invasive and it is flat, covering and dotted with micro-pits along the very edge. Further, it is domed and well linked. The limit between the polish and the unaffected zone on ventral face is irregular and progressive (lace-like).

On ventral face, polish is marginal, domed and well linked. The limit between the unaffected zone and the polished one is regular but progressive (lace-like).

Striations: Under low power, macro-striations perpendicular to the edge are visible, as shown in picture A.

The very developed polish on the very edge shows micro-striations perpendicular to the edge. They are very narrow, short, shallow, straight, and slightly darker than the polish.

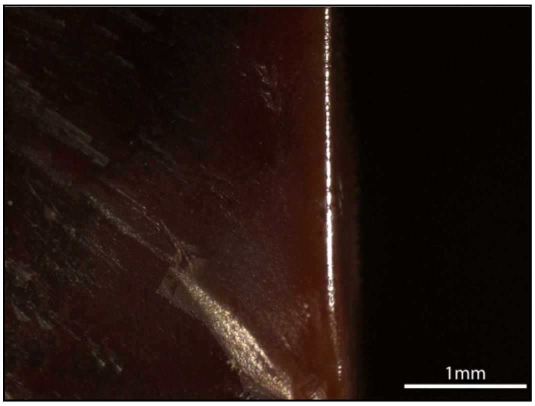

EX12-106A: Flat glossy rounding of the very edge that presents macro-striations perpendicular to the edge.

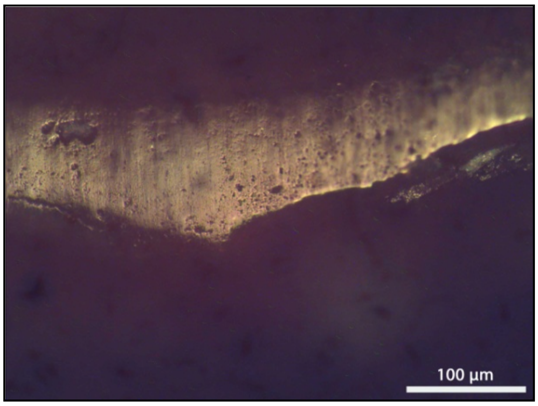

EX12-106B: Flat covering polish on the very edge. Micro-pits and narrow striations perpendicular to the edge.

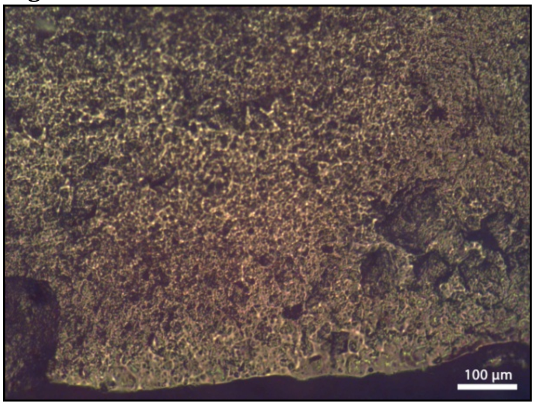

EX12-106C: Invasive polish, covering and flat along the very edge, then linked and domed further inside dorsal face.

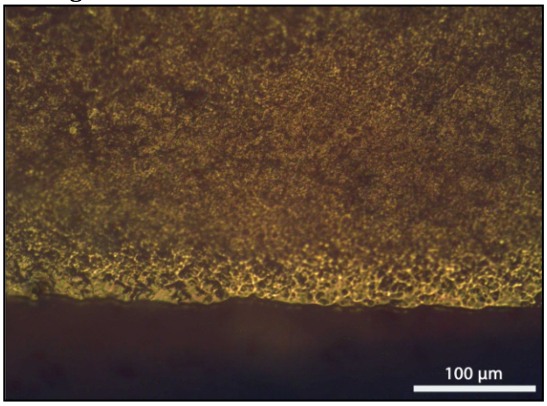

EX12-106D: Marginal polish on ventral face.

Lithic artefacts from Tabon Cave studied in the frame of this research:

All necessary permits were obtained for the described study, which complied with all relevant regulations. They were issued by the National Museum of the Philippines, located in Padre Burgos Avenue, Metro Manila, which is in charge of their curation.

Below are the accessioning numbers of the artefacts analysed. The manuscript focuses here on the use-wear and residues observed on three of them: P-XIII-T-299, 62-I-11821 and P-XIII-T-59.

62-I-10449
62-I-10563
62-I-11087
62-I-11303
62-I-11386
62-I-11539
62-I-11592
62-I-11670
62-I-11693
62-I-11708
62-I-11762
62-I-11763
62-I-11764
62-I-11815
62-I-11821
62-I-1960
62-I-7021
62-I-9897
P-XIII-T-1081
P-XIII-T-1124
P-XIII-T-1223
P-XIII-T-1248
P-XIII-T-1516
P-XIII-T-1686
P-XIII-T-1700
P-XIII-T-1829
P-XIII-T-1877
P-XIII-T-2178
P-XIII-T-2299
P-XIII-T-232
P-XIII-T-2328
P-XIII-T-2575
P-XIII-T-299
P-XIII-T-412

P-XIII-T-59
P-XIII-T-6067
P-XIII-T-640
P-XIII-T-7074
P-XIII-T-710
P-XIII-T-7634
Screening
Unaccessioned
Unreadable
