# Supplementary material for: The invisible plant technology of Prehistoric Southeast Asia: Indirect evidence for basket and rope making at Tabon Cave, Philippines, 39–33,000 years ago
Source: PLoS One. 2023 Jun 30;18(6):e0281415. doi: 10.1371/journal.pone.0281415 (PMC10313078; doi:10.1371/journal.pone.0281415)
Supplement: Attachment — Submitted filename: Xhauflair et al - Response to the Editor-23 dec 2022.docx [file pone.0281415.s004.docx]

Dear Dr Hardy,

Thank you very much for your comments which helped us to improve the style of the paper. The whole text was revised by a professional copy-editor and proof reader: Mr Robert Morgan. In addition, we addressed each of the specific points you raised.

Please find below our responses to your specific comments, in green.

Wishing you a Merry Christmas and a Happy New Year!

**Dr Hermine Xhauflair**

Associate Professor

Archaeological Studies Program

University of the Philippines Diliman

Lakandula Street, 1101 Quezon City, Manila

Philippines

First page Introduction – not a good sentence.   Do you mean –Raw materials extracted from both plants and animals constitute ….

- We changed into: “Plants constitute with animals the sources of raw materials of what Hurcombe calls “the missing majority”.”

Line 210 change ‘frame’ to ‘framework’ > Done

Line 227 - a 3 month fieldwork season, or 3 months of fieldwork

- We changed into “a 3 month fieldwork season”

Be consistent in the use of capital letters in your Tables > Each entry is now starting awith a capital letter.

Line 528: ‘Conclusion on the function of the analysed stone tools from Tabon Cave’  This is very confusing.  You should remove this heading, and simply move up the label Discusssion from below.

This is really part of the Results section:

1) The use-wear and residues observed on artefacts are presented,

2) We expose comparable use-wear on experimental tools

3) We show the ethnographic observations based on which the experiments were designed.

4) We get back to the archaeology artefacts showing what we can infer about their utilisation based on the experimental and ethnographic data.

We are offering to rename the last section :

“Understanding further the function of Tabon Cave artefacts based on experimental and ethnographic data”

Discussion.  We know you will be talking about your study so you do not need to state this. You will probably need to reorder the first part of this. .

- We changed the text into: “Diagnostic use-wear pattern characteristic of thinning plant fibres were determined on three stone tools from Tabon Cave dating back 39-33,000 years. We were able to identify this pattern because it is identical to the use-wear distribution observed”
- And into: For many decades, researchers working in Southeast Asia have formulated the hypothesis that simple and non-standardised lithic artefacts had been complemented by plant implements made of bamboo.[44,67–71] In contrast, our results constitute evidence for the existence of perishable plant-based technology in prehistoric Palawan, even though the number of archaeological artefacts displaying this thinning pattern is still limited. They show that early plant technology did indeed exist in tropical regions during the Pleistocene, but it was not focused simply on the manufacturing of bamboo tools. This complements our discovery that some of the denticulates from Tabon Cave had been used to split plants, an operation that is part of the manufacturing of many objects nowadays, including flooring, musical instruments, and darts.
- In fact, the human groups who inhabited Tabon Cave had developed a botanical knowledge deep enough to know which plants within their environment had fibrous, flexible, and solid properties, and could be turned into ropes, baskets, and other fibrecraft.
- As early as 39-33,000 years ago, the Late Pleistocene inhabitants of Palawan possessed an elaborate organic technology and were processing plant fibres to make cordage, baskets, traps, or other composite objects.

Line 645 ‘evidenced’ is incorrect. There is no verb ‘to evidence’. I suggest ‘indicated’

- Done

Line 682 It remains to explore and following…. Needs to be rewritten.

We rewrote this section into:

“More than 30 000 years later, these botanical knowledge and technological know-how are still alive and allow many communities all over Southeast Asia to produce objects necessary to answer their everyday needs in a sustainable way. It remains to be explored whether these contemporary practices are the fruits of a continuous tradition directly rooted into Late Pleistocene.”

Also, what has watch making in Switzerland to do with this paper? This needs to be removed. > We removed the reference to the tradition of watchmaking in Switzerland.

Line 698 - It had been hypothesised – by whom? Reference (even if it is a repeat)

Line 699 ‘and that the later ‘ this is unclear. Please rewrite.

What is the Bamboo Hypothesis? This is not specifically described in the ms-do you mean reference 66  If you are going to use it, you need to explain this more clearly and re-reference it here

- We rewrote the paragraph into:

“These results show that seemingly simple Southeast Asian artefacts are hiding testimonies of a behavioural complexity invisible to the naked eye but that we can reveal through use-wear analysis. It had been hypothesized by proponents of the Bamboo Hypothesis that Southeast Asian stone tools had been used mainly to manufacture bamboo tools and that the focus of prehistoric craft makers on this giant grass (Bamboo is a Poaceae) would explain the technological simplicity of lithic artefacts. [91,92,101,102] Our results add to the recent evidence showing that a plant-based perishable technology indeed existed in the region during Prehistory, but also add nuance to the Bamboo Hypothesis *sensu stricto*, showing that people invested on plant materials in a much broader sense and did not use their stone tools exclusively to make bamboo knives, arrows and darts.”

1.	Solheim WG. The “New look” of Southeast Asian prehistory. The Journal of the Siam Society. 1972;60: 1–20.

2.	Pope GG. Bamboo and human evolution. Natural history (USA). 1989 [cited 11 Apr 2014]. Available: http://agris.fao.org/agris-search/search.do?recordID=US9010157

3.	Forestier H. Des outils nés de la forêt. De l’importance du végétal en Asie du Sud-Est dans l’imagination et l’invention technique aux périodes préhistoriques. Acte du Séminaire-atelier Orléans, 15 et 16 octobre 1998. IRD Editions. Paris: Froment A. & Guffroy J.; 2003. pp. 315–337.

4.	Forestier H. La pierre et son ombre : épistémologie de la Préhistoire / Hubert Forestier (2020) - Société Préhistorique française. L’Harmattan. Paris; 2020. Available: http://www.prehistoire.org/offres/gestion/actus_515_40990-633/la-pierre-et-son-ombre-epistemologie-de-la-prehistoire-hubert-forestier-2020.html

Line 720 – ON other continents. Much longer time …. than what?  Rewrite. Do you mean a long time? Or longer than previously thought – in which case needs reference to the previous thought.

Line 713 ‘what was the’ – remove ‘what was’

- We rewrote the end of the Conclusion into:

Elsewhere in the world, a cord fragment made of twisted bark and dating to 46 ± 5 ka to 52 ± 2 ka was found at the Abris du Maras in France, and pierced shells from the Grotte des Pigeons, Morocco, and Blombos, South Africa constitute indirect evidence for the use of strings respectively 82 000 and 75 000 years ago.[9,10] Future analyses of lithic artefacts from Southeast Asia using the experimental reference we provided here can reveal if the antiquity of fibre technology was even greater than 39-33 000 BP in this region, what was the geographical extent of this craft, and if its contemporary practice is the result of an uninterrupted tradition.

Line 786 – correct the spelling of . Ibannez

- Corrected into: J. Ibañez

In addition, two references were added. We realised that we had forgotten to include them in the previous versions of the manuscript:

9.	Bouzouggar A, Barton N, Vanhaeren M, d’Errico F, Collcutt S, Higham T, et al. 82,000-year-old shell beads from North Africa and implications for the origins of modern human behavior. Proc Natl Acad Sci. 2007;104: 9964–9969. doi:10.1073/pnas.0703877104

10.	d’Errico F, Henshilwood C, Vanhaeren M, van Niekerk K. Nassarius kraussianus shell beads from Blombos Cave: evidence for symbolic behaviour in the Middle Stone Age. J Hum Evol. 2005;48: 3–24. doi:10.1016/j.jhevol.2004.09.002
