# Supplementary material for: The invisible plant technology of Prehistoric Southeast Asia: Indirect evidence for basket and rope making at Tabon Cave, Philippines, 39–33,000 years ago
Source: PLoS One. 2023 Jun 30;18(6):e0281415. doi: 10.1371/journal.pone.0281415 (PMC10313078; doi:10.1371/journal.pone.0281415)
Supplement: Attachment — Submitted filename: Xhauflair et al - response to the editor.docx [file pone.0281415.s005.docx]

**Response to the editor**

**Dear Dr Hardy,**

Thank you very much for taking the time to look in details into our manuscript, for helping us to improve it and for giving us an opportunity to learn during the process. Please find our responses to your proposed modifications below, in green.

There remain considerable challenges to understand parts of this ms.  Rather than send it back to you to ask again for corrections, I have made the suggestions myself.  I have only focused on places where the language needs to be corrected to ensure that the meaning is understood.

LINE 68,69. Quafzeh,  CORRECT SPELLING> We changed into **Qafzeh**

Line 156 and line 202. ‘frame’ should be ‘framework’ >done

Line 319 ‘On this ventral face, the polish is flat, shiny and covering, cut by dark striations’

You need to change this sentence to explain what you mean by ‘covering’.  What was covered?  Do you mean for example, that it covered the entire surface? If so, you need to explain this.  Please clarify and correct.

This is a description of the degree of linkage of the polish. The categories we use to describe the polishes are detailed in the Supporting information, so we also referred to it. We changed the sentence into the following:

“On this ventral face the polish is flat, shiny, **with a high degree of linkage (covering [59]-see the Supporting information p.11)** , cut by dark striations”

*Lines 423 ff Occasionally, we witnessed 424 this operation performed with a large machete (tukäw) when we were in the forest and under 425 circumstances in which someone wanted to show usindicate how this operation was done on 426 the spot, pointing out that the appropriate tool was normally the smaller knife called paqis 427 (that is usually kept at home and not carried along like thein the manner of a machete).*

This long sentence is very difficult to follow.

Suggest change to

Occasionally, in the forest we witnessed this operation performed with a large machete (tukäw). This was done when an individual summarily wanted to show us how this operation was done. However, on each occasion, they pointed out to us that the appropriate tool would normally be the smaller knife called *paqis* (that is usually kept at home and not carried along in the manner of a machete).

- Thank you very much! It is much clearer this way. We changed accordingly.

Line 462. ‘During our stay, weaving baskets was always performed by women.’

This should be ‘*During our stay, basket weaving was always performed by women*.’

- Thank you very much. We changed accordingly.

Line 467 ‘Even if making weaving strips is a female activity for the Pala’wan communities we stayed 468 with, It was men who also knew how to process the plant segments to make weaving 469 stripsfor this purpose because (the involving the same chaîne opératoire is the same than for 470 as making ties.) As a demonstration of this, and itthe person was a man, (Linggit Rilla,) who 471 showed us how to process the bamboo vine Dinochloa sp. and Donax canniformis was a man: 472 Linggit Rilla.’

*HOW CAN YOU BE SURE THAT THIS IS BECAUSE IT IS THE SAME CHAINE OPERATIORE? YOU NEED TO DEMONSTRATE THIS, OR REMOVE IT.*

This whole part is confusing and potentially problematic. Do you mean you had one person, a man, showing you how to make the weaving strips, yet you ‘know’ that in fact it was always women? How do you know? Why did only one person show you this and why, in this case, was it a man and not a woman, since this was a woman’s task?

This is very difficult to follow. Suggest the following

‘Though making weaving strips is a female activity for the Pala’wan communities we stayed 468 with, men also knew how to process the plant segments for weaving strips since the same chaîne opératoire was used to make ties. For example, the person who showed us how to process the bamboo vine Dinochloa sp. and Donax canniformis was a man: 472 Linggit Rilla.’

- Thank you very much again. This is a good formulation. We replaced the sentence accordingly.
- We know that weaving baskets is a female activity because that is what people told us repeatedly and because this is what we witnessed during a 3-month fireldwork and subsequent visits. This information was also published by Nicole Revel, an ethnographer who studied Pala’wan culture for more than 45 years. We added a reference to one of her books which mentions that fact as well.

Line 501. 3.4. Understanding further the function of Tabon Cave 502 artefacts based on experimental and ethnographic 503 data. SUGGEST AS FOLLOWS;

3.4. DEVELOPING AN UNDERSTANDING OF the function of Tabon Cave 502 artefacts based on experimental and ethnographic 503 data

> Ok. We changed accordingly.

Line 554 Mainly the contact face but sometimes also the non contact face exhibit REMOVE S HERE directional markers in perpendicular and diagonal orientation to the edge; THESE are often numerous and indicate a repeated transversal motion. These directional markers consist REMOVE S HERE of VARIABLE striations. THESE CAN BE EITHER long and parallel **and can form larger sets parallel between them**  **or long trails that materialise the contact with the processed plant strips;** **undulations of fluted 560 polish; , stretched-out polish components forming lines; , and/or polish expanding inside the 561 tool surface.** THE MEANING OF THE PART IN **BOLD** ESCAPES ME. PLEASE ADD IN AN EXPLANATORY DIAGRAM. PLEASE REMEMBER THAT THIS IS A BROAD JOURNAL, THE PEOPLE READING YOUR ARTICLE MAY NOT BE SPECIALISTS IN USE WEAR.

- There is already a visual explaining these features: Figure 6.
- We added a reference to Fig 6 at the end of the paragraph.

“[…] polish components forming lines, and/or polish expanding inside the tool surface. **(Fig 6)**”

LINE 584 from Tabon Cave had been used to split plants, an operation that is part of the 585 manufacturing of many objects nowadays, including flooring, musical instruments, and darts.

I assume here you mean that the split plants are part of the many objects.  This is currently unclear. Suggest as follows:

Tabon Cave had been used to split plants. Split plants form component parts NOTE I HAVE ADDED AN S HERE of many objects nowadays, including flooring, musical instruments, and darts.

- We modified accordingly

Line 592 ‘Establishing experimental reference collections of use-wear resulting from realistic activities 593 based on observations of skilled craft makers in ethnographic contexts is a powerful means 594 of recovering evidence of perishable material culture’

Clunky and hard to follow. Suggest as follows:

Establishing experimental USE WEAR reference collections CREATED AS A RESULT OF realistic activities 593 based on observations of skilled craft makers in ethnographic contexts, OFFERS A powerful TOOL TO  recover evidence of perishable material culture, AS WE DEMONSTRATE HERE, AND AS ADVOCATED ELSEWHERE … ’

- We replaced accordingly.

Line 627 Archaeological evidence for these fishing practices since the Late 628 Pleistocene has been found

Suggest as follows: Archaeological evidence for these fishing practices has been found from the Late Pleistocene onwards

- We changed accordingly.

Line 630.  ‘from the forest’, If not the forest, then where? Please clarify.

- We changed into:

“Kerfant [19] highlighted the interesting aspect that most of the plants used in seafaring come from forests **away from the coast**, implying the mastery of both marine and inland environments.”

Line 636 More generally, tying materials allowed the development of all a great many kinds of 637 composite technologies.

SUGGEST AS FOLLOWS:

More generally, tying materials ENABLED THE DVELOPMENT OF AN EXTENSIVE composite technology.

>We changed into:

” More generally, tying materials **enabled the development of composite technologies.”**

(We do not know yet how extensive it was)

LINE 638. WHAT IS ADDITIVE TECHNOLOGY? PLEASE CLARIFY AND CORRECT.

- This is a term used by André Leroi-Gourhan to qualify rope and basket technology (the idea is that matter is added and not removed). We changed into “**fibre technology”** for more clarity.

LINE 645 what was reported here. CHANGE TO ‘those we report here’ > Done

LINE 655 s had in fact the great technological expertise and the 656 advanced skills necessary for thinning plant fibres [

HERE AS YOU HAVE WRITTEN IT, IT IMPLIES THAT  THEIR GREAT TECHNOLOGICAL EXPERTISE IS *ONLY* THEIR advanced skills necessary for thinning plant fibres [75] and possibly for assembling strips to 657 manufacture three dimensional objects.. I DO NOT THINK YOU ACTUALLY MEAN THIS, BUT YOU NEED TO EXPLAIN WHAT YOU MEAN.

>We added some examples of other technological skills and innovation from the region :

“In line with other studies **showing for instance that the earliest representation of a scene is from Sulawesi, or that people had a mastery of navigation** (e.g. [18,85,86,101,102]), our results contribute to rehabilitate Southeast Asian prehistoric heritage by showing that prehistoric groups had in fact great technological expertise and advanced skills**, including the ones necessary for thinning plant fibres** [75] and possibly for assembling strips to manufacture three dimensional objects.”

We hope that it is clearer (and more correct) this way.

We added one new reference:

102.	Aubert M, Lebe R, Oktaviana AA, Tang M, Burhan B, Hamrullah, et al. Earliest hunting scene in prehistoric art. Nature. 2019 [cited 13 Dec 2019]. doi:10.1038/s41586-019-1806-y

LINE 674 into the Late Pleistocene – SHOULD BE IN NOT INTO

- We modified accordingly

LINE 689 It had been hypothesized by 690 proponents of the Bamboo Hypothesis that Southeast Asian stone tools had been used 691 mainly to manufacture bamboo tools a

SUGGEST AS FOLLOWS:  OUR RESUTS SUPPORT The Bamboo Hypothesis that Southeast Asian stone tools had been used 691 mainly to manufacture bamboo tools a

- We cannot say that because our results **nuance** the Bamboo Hypothesis *sensu stricto*. Here it is really important to keep our argumentation as it is.

LINE 703 FF. THIS IS A REPETITION YOU DO NOT NEED TO REPEAT IT HERE. elsewhere in the world, a cord fragment made of twisted bark and dating to 704 46 ± 5 ka to 52 ± 2 ka was found at the Abris du Maras in France, and pierced shells from the 705 Grotte des Pigeons, Morocco, and Blombos, South Africa constitute indirect evidence for the 706 use of strings respectively 82 000,000 and 75 000,000 years ago.[9,10]

- We removed this sentence.

LINE 709 we provided – remove the d. should be provide

Done.

**ADDED REFERENCE:**

102.	Aubert M, Lebe R, Oktaviana AA, Tang M, Burhan B, Hamrullah, et al. Earliest hunting scene in prehistoric art. Nature. 2019 [cited 13 Dec 2019]. doi:10.1038/s41586-019-1806-y
